# Synthesis, Cytotoxicity and Anti-Proliferative Activity against AGS Cells of New 3(2*H*)-Pyridazinone Derivatives Endowed with a Piperazinyl Linker

**DOI:** 10.3390/ph14030183

**Published:** 2021-02-25

**Authors:** Mehmet Abdullah Alagöz, Zeynep Özdemir, Mehtap Uysal, Simone Carradori, Marialucia Gallorini, Alessia Ricci, Susi Zara, Bijo Mathew

**Affiliations:** 1 Department of Pharmaceutical Chemistry, Faculty of Pharmacy, İnönü University, Malatya 44280, Turkey; mehmet.alagoz@inonu.edu.tr (M.A.A.); zeynep.bulut@inonu.edu.tr (Z.Ö.); 2 Department of Pharmaceutical Chemistry, Faculty of Pharmacy, Gazi University, Ankara 06010, Turkey; mgokce99@gmail.com; 3 Department of Pharmacy, “G. d’Annunzio” University of Chieti-Pescara, via dei Vestini 31, 66100 Chieti, Italy; marialucia.gallorini@unich.it (M.G.); alessia.ricci@unich.it (A.R.); susi.zara@unich.it (S.Z.); 4 Department of Pharmaceutical Chemistry, Amrita School of Pharmacy, Amrita Vishwa Vidyapeetham, AIMS Health Sciences Campus, Kochi 682 041, India

**Keywords:** 3(2*H*)-pyridazinone, hydrazone, fibroblasts, gastric cancer, piperazine, Bax, H_2_O_2_ release

## Abstract

Novel twenty-three 3(2*H*)-pyridazinone derivatives were designed and synthesized based on the chemical requirements related to the anti-proliferative effects previously demonstrated within this scaffold. The introduction of a piperazinyl linker between the pyridazinone nucleus and the additional (un)substituted phenyl group led to some compounds endowed with a limited cytotoxicity against human gingival fibroblasts (HGFs) and good anti-proliferative effects against gastric adenocarcinoma cells (AGS) as evaluated by MTT and LDH assays, using doxorubicin as a positive control. Successive analyses revealed that the two most promising representative compounds (**12** and **22**) could exert their effects by inducing oxidative stress as demonstrated by the hydrogen peroxide release and the morphological changes (cell blebbing) revealed by light microscopy analysis after the haematoxylin-eosin staining. Moreover, to further assess the apoptotic process induced by compounds **12** and **22**, Bax expression was measured by flow cytometry. These findings enlarged our knowledge of the structural requirements in this scaffold to display valuable biological effects against cancerous cell lines.

## 1. Introduction

Gastric cancer (GC) is the third cause of cancer death in the world, despite a declining incidence due to improvements in screening methods and *Helicobacter pylori* eradication. Its symptomatology (early satiety, nausea, vomiting, asthenia, anaemia and weight loss) can be appreciated only when it has grown in size interfering with the nutritional process [1]. Complete surgical resection of the whole tumoral mass remains the first-line protocol for GC treatment, but being often diagnosed at an advanced stage, this malignant tumor can frequently be not operable [2]. Individualised treatments, involving radiotherapy and chemotherapy, only provide modest results especially in advanced or metastatic GC and due to the existence of four molecular subtypes of GC some chemotherapeutics may be unsuccessful. Moreover, trials with molecular-targeted therapy have provided contrasting results suggesting an urgent need to propose the development of different therapeutic protocols or new drugs for prolonging the survival of the fragile patients with GC [3]. Among the classical anti-GC drugs, 5-fluorouracil and doxorubicin display a potent anti-proliferative activity, while exerting important side effects and inducing the development of drug-resistant cancer cells [4].

With the aim of proposing an alternative therapeutic arsenal, our research group explored innovative chemical scaffolds [5,6], confirming how the introduction of nitrogen-containing heterocycles could be the driving force to achieve promising preliminary results [7]. Among the large plethora of nitrogenous heterocycles, pyridazinone compounds are of great interest to researchers because of their various biological effects [8,9]. These activities include anti-inflammatory, analgesic, anti-viral, anti-parasitic, anti-fungal, anti-bacterial anti-diabetic, anti-tubercular, anti-depressant, anti-hypertensive, anti-thrombotic, anti-feedant, and diuretic effects [10,11,12,13,14,15,16,17,18,19,20]. In addition, pyridazinone compounds have been also reported to have anticancer activity [21,22], and an interesting group of 3(2*H*)-pyridazinone derivatives have been synthesized and evaluated for their anti-proliferative effects against human colon carcinoma HCT116 cells [23]. These results suggest that pyridazinone compounds may be useful in cancer chemotherapy and that, according to the proposed SARs [23], pyridazinone derivatives bearing different substituents may exhibit varying degrees of cytotoxic effect.

In the present study, 23 new 3(2*H*)-pyridazinone-based compounds, which are thought to be effective against gastric adenocarcinoma (AGS cells) and have low toxicity, were synthesized and their biological activities were evaluated. In the design of these compounds, we kept into consideration the role exerted by the piperazine linker introduced between the pyridazinone core nucleus and the additional phenyl ring to display anti-proliferative effects against liver (HEP3B), colon (HCT116) and neuroblastoma (SHSY5Y) cell lines as previously reported [24,25].

These compounds were first submitted to a general screening for their toxicity against human gingival fibroblasts and AGS cells at established concentrations (10 and 50 µM) to select the most promising derivatives for further biological assessment of their possible antineoplastic activity evaluated by means of MTT test, lactate dehydrogenase (LDH) assay, light microscopy analysis, hydrogen peroxide (H_2_O_2_) release and Bax immunostaining by flow cytometry. All the results have been compared to those of doxorubicin as a positive control drug for gastric adenocarcinoma [26].

## 2. Results and Discussion

**Scheme 1:**
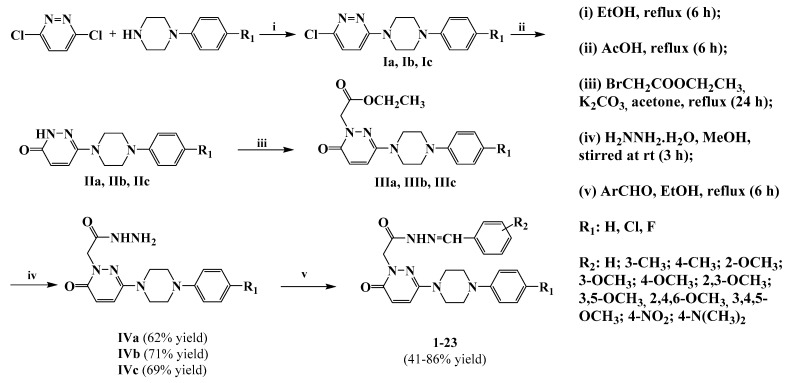
Synthesis of compounds **1**–**23**.

The novel compounds **1**–**23** were obtained by reacting different benzaldehyde derivatives with intermediates **4a**–**c**, which were in turn prepared according to the literature [12,27,28], as shown in Scheme 1.

As a result of the nucleophilic substitution reaction of 3,6-dichloropyridazine and the appropriate substituted phenylpiperazine, compounds **Ia**–**c** were obtained. Subsequently, by heating these compounds in glacial acetic acid, the pyridazine ring turned into a pyridazinone ring by hydrolysis. Compounds **IIIa**–**c** were synthesized by the reaction of ethyl bromoacetate with **IIa**–**c** in acetone in the presence of potassium carbonate. Substituted pyridazinone-2-yl acetohydrazide compounds **IVa**–**c** were obtained by the condensation reactions of hydrazine hydrate (99%) with **IIIa–c**. The new title compounds **1–23** were synthesized by the reaction of **IVa**–**c** with some benzaldehyde derivatives in ethanol and listed in Table 1. The reaction yields for the last step of the synthesis, after recrystallization from methanol/water, of the compounds have a wide range (41 to 86%). ^1^H-NMR, ^13^C-NMR, and mass spectra of compounds **1**–**23** are given as Supplementary Data.

**Figure 1:**
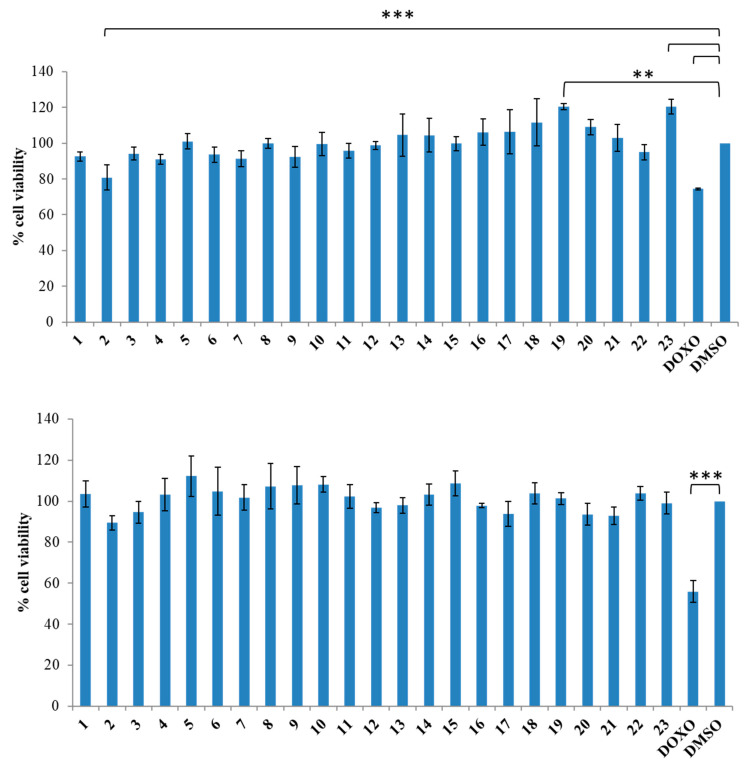
MTT test on HGFs treated with compounds **1**–**23** and with doxorubicin (DOXO) at 10 µM for 48 (**upper graph**) and 72 h (**lower graph**). *** *p* < 0.0002, ** *p* < 0.0005. DMSO: control vehicle.

The capability of compounds **1**–**23** to affect cell viability was evaluated by means of the MTT test which measures the metabolic activity of viable cells. Firstly, the MTT test was carried out on primary HGFs, non-pathological cells, extracted from gingiva withdrawal of healthy donors. The effect of the compounds **1**–**23** was compared with the effect of doxorubicin, an antitumoral drug commonly used in the therapeutic protocols. Our control was represented by HGFs only treated with the vehicle DMSO. Compounds **1**–**23** were administered to HGFs up to 72 h at 10 µM evaluating the cell viability after both 48 and 72 h of treatment (Figure 1, upper and lower graphs). After 48 h of treatment only compound **2** and the doxorubicin are able to significantly reduce cell viability respect to DMSO-treated cells. Conversely, compounds **19** and **23** significantly increase this parameter with respect to DMSO-treated cells (upper graph). After 72 h of treatment only doxorubicin discloses a significant reduction in cell viability respect to control (lower graph).

**Figure 2:**
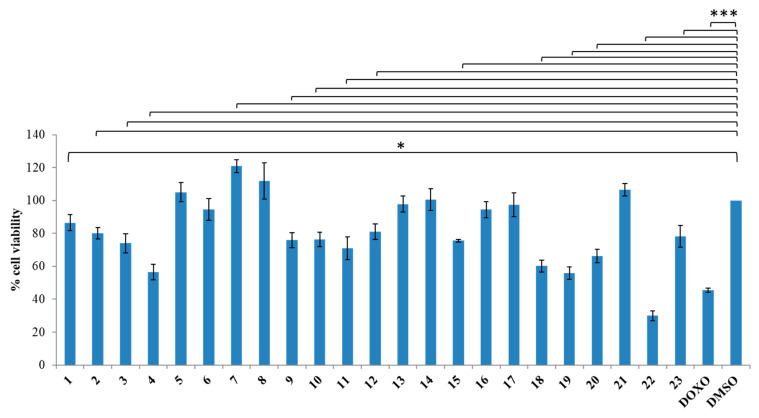

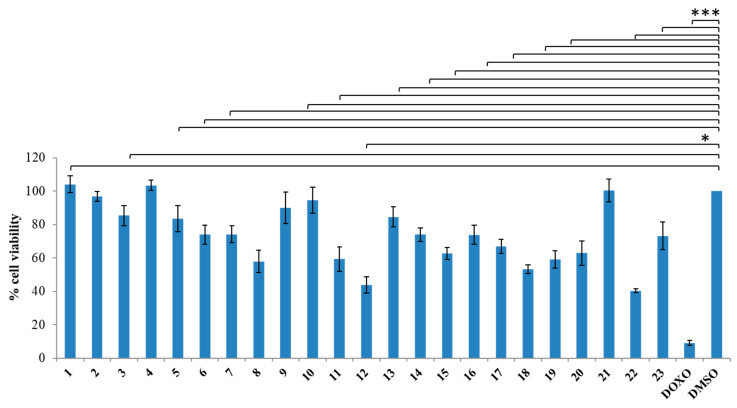
MTT test on AGS treated with compounds **1**–**23** and with doxorubicin (DOXO) at 50 µM for 48 (upper graph) and 72 h (lower graph). *** *p* < 0.0002, * *p* < 0.005. DMSO: control vehicle.

Secondly, the administration of compounds **1**–**23** and doxorubicin for 48 and 72 h was performed on a tumoral cell line of gastric adenocarcinoma (AGS). The newly synthetized molecules and doxorubicin were tested at 50 and 10 µM. When cells are treated with the higher dose of compounds **1**–**23** and doxorubicin (50 µM), most of them are able to significantly reduce cell viability, both after 48 and 72 h (Figure 2, upper and lower graphs).

**Figure 3:**
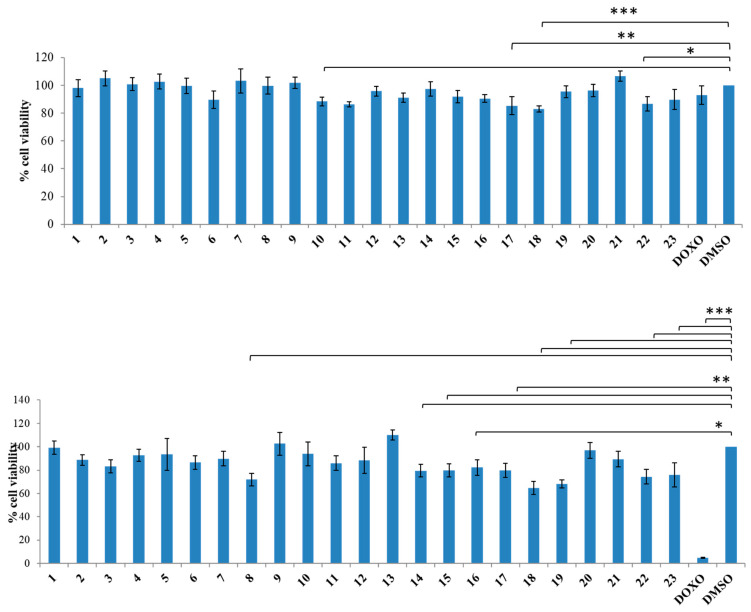
MTT test on AGS treated with compounds **1**–**23** and with doxorubicin (DOXO) at 10 µM for 48 (**upper graph**) and 72 h (**lower graph**). *** *p* < 0.0002, ** *p* < 0.0005 * *p* < 0.005. DMSO: control vehicle.

When AGS are treated with the lower dose (10 µM) after 48 h of treatment only the molecules **11**, **17**, **18** and **22** show a statistically significant decrease of AGS metabolic activity (Figure 3, upper graph), whereas, after 72 h of treatment several compounds (**8**, **14**–**19**, **22**, **23** and doxorubicin) show the capability to negatively affect the cell viability respect to DMSO treated AGS (Figure 3, lower graph).

These preliminary data appear very promising considering that, except for a few of them and doxorubicin, they show an appreciable capability not to interfere with the proliferation of non-tumoral cells thus protecting them from any harmful effects. At the same time, compounds **17**, **18** and **22** at 50 µM concentration revealed an appreciable capability to strongly counteract gastric adenocarcinoma cell proliferation after both 48 and 72 h of administration, whereas compounds **8** and **12** were effective only at 50 µM concentration after 72 h. Collectively, these results suggested that the aryl ring attached to the piperazine linker must be *para*-halogen (chloro or fluoro)-substituted, whereas the aryl ring on the hydrazido chain must have electron-donating or electron-withdrawing groups at the *meta* or *para* position, respectively, to display anti-proliferative effects against AGS cells.

**Figure 4:**
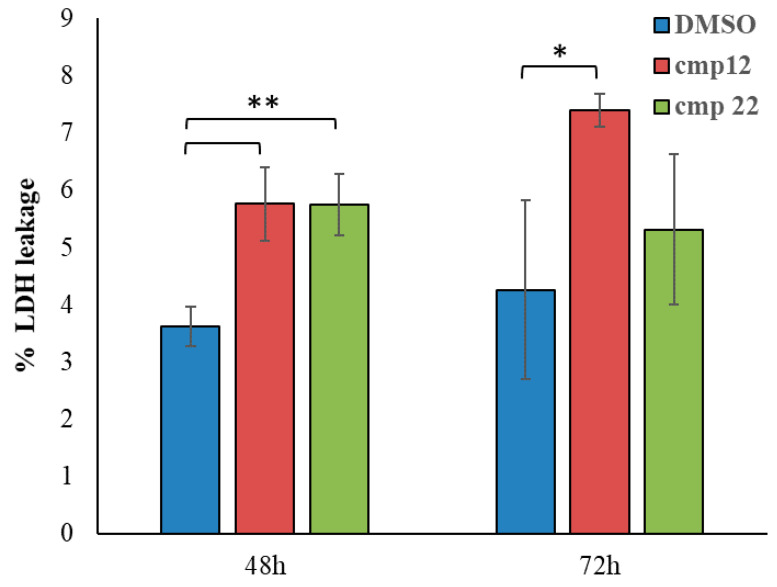
Cytotoxicity assay of AGS gastric adenocarcinoma cells treated with molecules vehicle (DMSO), **12** and **22** for 48 and 72 h. LDH leakage is reported as the percentage of LDH leakage compared with vehicle treated cells (DMSO). Data are presented as the mean ± standard deviation of three separate experiments. ** *p* < 0.01, * *p* <0.05.

Basing on these screening results, compounds **12** and **22** were chosen to perform specific assays with gastric adenocarcinoma cells (AGS) in order to better understand the molecular mechanisms underlying the biological effect of these molecules. In particular, **12** and **22** were selected because of their better ability to reduce the proliferation of AGS cells at 50 µM after 72 h, whereas they were safe and tolerated at 10 µM against both HGFs and AGS cells. Firstly, a cytotoxicity assay which measures the lactate dehydrogenase (LDH) released within the culture medium, was carried out after 48 and 72 h of treatment with **12** and **22** on AGS cells (Figure 4).

**Figure 5:**
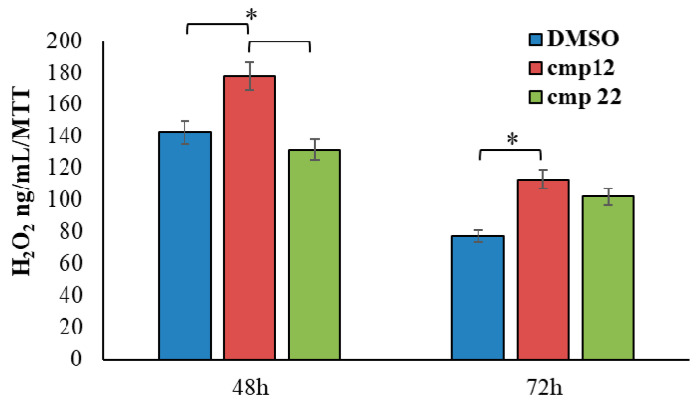
Quantitative determination of the H_2_O_2_ released within the culture medium by AGS treated with vehicle (DMSO), **12** and **22**, for 48 and 72 h. Values represent the means ± SD of three separate experiments. The bar graph shows the H_2_O_2_ release (ng/mL) in cell supernatants; * *p* < 0.05.

After 48 h of culture, both **12** and **22** treatments caused a statistically significant increase (*p* < 0.01) in LDH release compared to LDH release measured in DMSO sample. After 72 h of treatment this trend is maintained for AGS treated with **12** (*p* < 0.05), whereas a partial recovery for AGS treated with **22** could be hypothesized. Secondly, in order to evaluate the oxidative stress occurrence in AGS after administration of the two selected molecules, the H_2_O_2_ release within the culture medium was performed by an ELISA assay (Figure 5). The H_2_O_2_ release follows a similar trend to LDH release.

**Figure 6:**
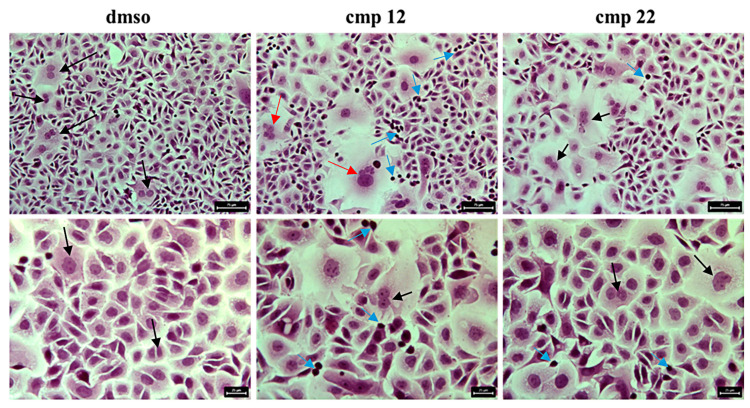
Haematoxylin-eosin staining of AGS treated with molecules vehicle (DMSO), **12** and **22**, for 48 h; upper line magnification 20×, lower line magnification 40×. Black arrows indicate mitotic cells, blue arrows indicate suffering or dead cells, red arrows indicate multinucleated cells.

**Figure 7:**
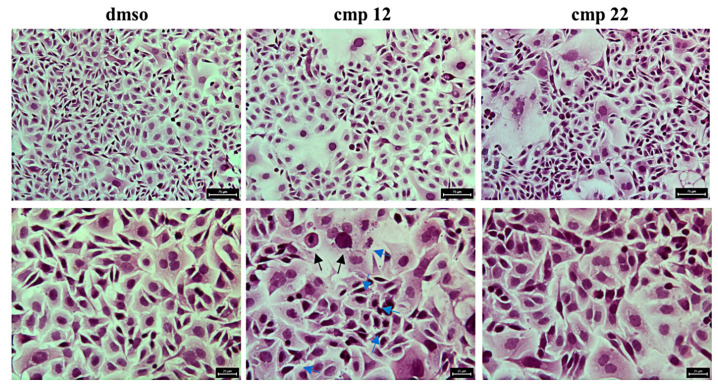
Haematoxylin-eosin staining of AGS treated with molecules vehicle (DMSO), **12** and **22**, for 72 h; upper line magnification 20×, lower line magnification 40×. Black arrows indicate cells with modified chromatin, blue arrows indicate cells surrounded by little vesicles.

After 48 h of treatment a statistically significant increase in H_2_O_2_ levels of AGS treated with **12**, compared to H_2_O_2_ levels of both vehicle-treated and **22**-treated AGS, can be detected (*p* < 0.05). Again, after 72 h of administration, H_2_O_2_ release within the culture medium appears significantly augmented only when AGS are exposed to **12** (*p* < 0.05). Thirdly, a morphological analysis of AGS treated for 48 and 72 h with DMSO and with the two selected molecules, was carried out by means of haematoxylin-eosin staining (Figure 6 and Figure 7).

The morphological analysis put in evidence, after 48 h of treatment (Figure 6) in DMSO-treated AGS, an appreciable amount of cell mitosis (black arrows) along with a higher cell density respect to both **12** and **22**-treated AGS. In **12**-treated AGS and, to a lower extent, in **22**-treated AGS, the occurrence of mitotic events appears negatively affected by the molecules administration as already evidenced by MTT test. Moreover, in **12** and **22**-treated cells, several round shaped and darker cells, close to detachment and cell death are clearly detectable (blue arrows). In AGS treated with **12**, some larger and multinucleated cells are also recognizable (red arrows).

After 72 h of treatment the previously described trend is maintained (Figure 7), however, in **12**-treated sample, some cells with modified chromatin distribution (black arrows) start to appear along with several cells surrounded by numerous little vesicles (blue arrows), a morphological condition resembling to blebbing phenomenon.

**Figure 8:**
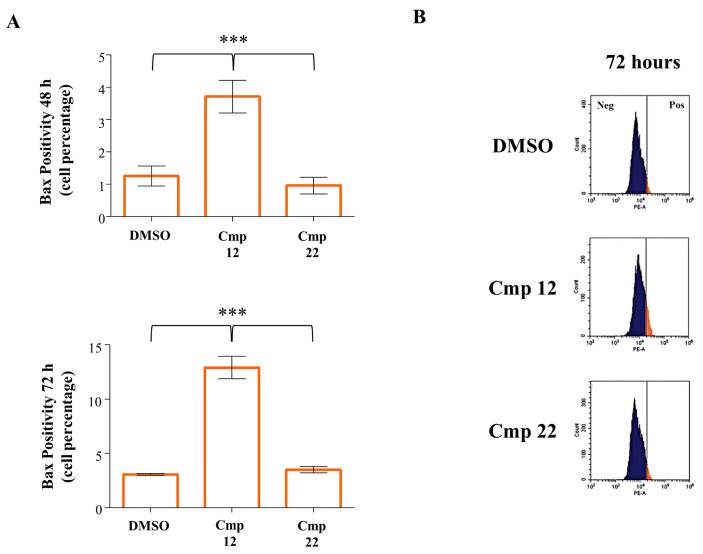
Bax expression in AGS cells in the presence of **12** and **22** after 48 and 72 h. (**A**) Bar graphs represent cell percentage of positive cells for Bax after 48 (upper histogram) and 72 h (lower histogram). (**B**) Representative peaks of treatment from the three independent experiments of the fluorescence emissions in the FL-2/PE channel. *** *p* < 0.0001 between DMSO and compounds.

Blebbing is often a typical feature of cells close to the apoptotic event [29,30]. Basing on this, to establish if **12** and **22** are able to trigger the apoptotic cascade thus explaining the rate of dead cells revealed by the viability test, Bax pro-apoptotic factor expression was evaluated through flow cytometry. Bax expression was measured in AGS cells after a 48 and 72 h of exposition to compounds **12** and **22** (Figure 8). The expression of the pro-apoptotic protein increases both at 48 and 72 h in the presence of compound **12** with respect to DMSO alone, while exposure to compound **22** is not effective as regard Bax levels of expression. In details, the percentage of Bax-positive cells significantly raises in a time-dependent manner, being assessed at 3.71% after 48 h (1.25% for DMSO alone) and 12.9% after 72 h (3.07% for DMSO alone).

### In Silico Pharmacokinetic Parameters and Target Prediction of the Most Promising Compounds ***12*** and ***22***

In Table 1 and Table 2 we have reported the medicinal chemistry parameters obtained from the web tool SwissADME [31], describing some properties of the tested compounds that are crucial for the future drug development. Collectively, the two compounds fully comply with the limits of the Lipinski’s rule (no violation), supporting their feasible oral use and drug-likeness, as also further corroborated by their high passive absorption in the gastrointestinal tract. Finally, they were not categorized as Pan Assay Interference Compounds (PAINS substructures able to elicit promiscuous pharmacological behaviour.

The major drawback of these compounds, and likely of the entire scaffold proposed in this paper, is the propensity to be substrate of the permeability glycoprotein (P-gp), thus promoting the energy-dependent efflux out of the cytosol and reducing their antiproliferative efficacy. This behaviour is also the limiting drawback of doxorubicin.

**Figure 9:**
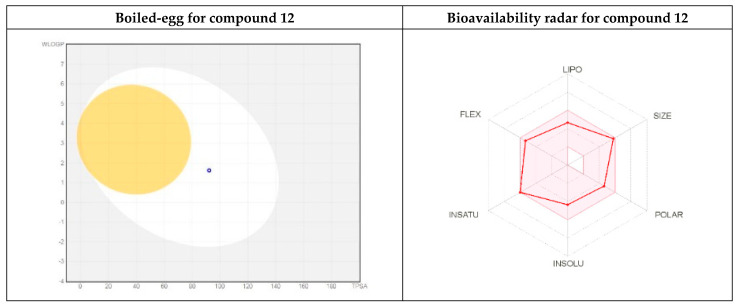

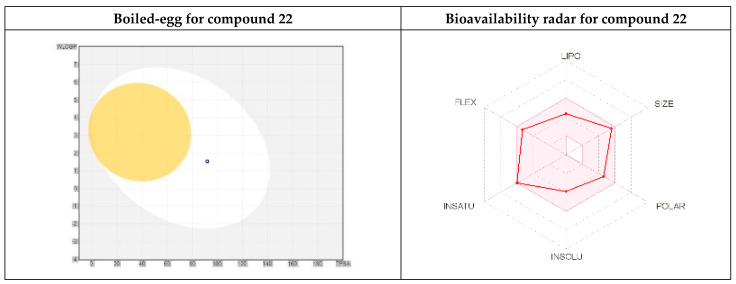
Representation of the boiled-egg graph and bioavailability radar calculated by SwissADME web-tool for compounds **12** and **22**.

Moreover, we also reported in Figure 9 the boiled-egg and bioavailability radar pictures as the two graphical outputs of the SwissADME tool, which considers several important parameters for determining the drug-likeness of a molecule. It allowed us to predict simultaneously two ADME characteristics: the passive absorption at the gastrointestinal tract (white area) and the ability to permeate the blood brain barrier (BBB, yellow area). Both the compounds are located in the yellow area implying that they are not likely able to cross the BBB, but can be passively absorbed at the GI level. Furthermore, the bioavailability radars provides the drug-likeness representation of the two compounds within the desirable pink area indicative for the optimal range of each physicochemical properties (lipophilicity, size, polarity, solubility, unsaturation, flexibility) for the oral administration.

With the aim to identify the putative target(s) of our two compounds, we also applied the SwissTargetPrediction web-tool, performing the analysis on the two compounds. In Table 3 and Table 4 we reported the data concerning the most probable targets found, i.e., the ones exhibiting the highest values of probability to be a target. Unfortunately, both compounds did not display a main preference for one target, nor with a high probability value, despite some of those can be related to cancer cell growth and survival.

## 3. Material and Methods

### 3.1. Chemical Studies

All chemicals used in the synthesis of compounds are products supplied by Sigma Aldrich (Merck, Milan, Italy). The synthesis of compounds in the structure of *N*′-benzylidene-2-(substituted)pyridazine acetohydrazide, as seen in Scheme 1, consists of five steps and each synthesis was performed according to the literature [32,33]. Impurity monitoring at each stage of the syntheses was performed by TLC (Kieselgel F_254_ plates, Merck, Milan, Italy) method. Melting points of the compounds were measured with a IA9000 Melting Point Apparatus (Cole-Parmer, Staffordshire, UK) and checked with the literature data. IR spectra were taken by the ATR technique (Spectrum 3 FT-IR Spectrometer, Perkin Elmer, Milan, Italy). ^1^H-NMR and ^13^C-NMR spectra were obtained in DMSO-*d*_6_ at 600 MHz and 150 MHz, respectively. An Avance Ultrashield FT-NMR spectrometer (Bruker, Billerica, MA, USA) was used for these measurements. Mass spectra were recorded using an Agilent 6400. B.08.00 (B8023.0) system (Agilent Technology, Santa Clara, CA, USA). Elemental analyses of the compounds were performed with a CHNS932 ibstrument (Leco, St. Joseph, MI, USA). All analyses of the compounds were done at the Scientific and Technological Research Center of Inonu University.

#### 3.1.1. Synthesis of Intermediate Compounds

*Synthesis of 6-(4-(4-chlorophenyl/4-fluorophenyl/phenyl)piperazine-1-yl)-pyridazines***Ia**–**c**. 4-Chlorophenyl/4-fluorophenyl/phenyl piperazine (0.01 mol), 3,6-dichloropyridazine (0.01 mol) and ethanol (15 mL) were added to a flask. The mixture was stirred under reflux for 6 h. The reaction mixture was poured into ice water. The solid phase was separated by filtration and dried. For purification, the residue was crystallized from ethanol. The melting points of 6-(4-(4-chlorophenyl/4-fluorophenyl/phenyl)piperazin-1-yl)-pyridazines **Ia–c** were compatible with the literature data [34].

*Synthesis of 6-(4-(4-chlorophenyl/4-fluorophenyl/phenyl)piperazin-1-yl)-3(2H)-pyridazinones***IIa**–**c**. Glacial acetic acid (30 mL) was added to the appropriate 6-substituted-3-chloropyridazine (0.05 mol). The mixture was stirred under reflux for 6 h. Glacial acetic acid was removed from the reaction medium with a rotary evaporator. The residue was extracted by adding water and chloroform. The organic phase was separated, sodium sulphate was added to dry, and then filtered. The chloroform phase was evaporated under reduced pressure. The residue was crystallized from ethanol. The melting points of 6-(4-(4-chlorophenyl/4-fluorophenyl/phenyl)piperazin-1-yl)-3(2*H*)-pyridazinones **IIa**–**c** were in accordance with the literature [12,27].

*Synthesis of ethyl 6-(4-(4-chlorophenyl/4-fluorophenyl/phenyl)piperazin-1-yl)-3(2H)-pyridazinone-2-ylacetates***IIIa**–**c**. 6-(4-(4-Chlorophenyl/4-fluorophenyl/phenyl)piperazin-1-yl)-3(*2H*)-pyridazinone (0.01 mol), potassium carbonate (0.02 mol), ethyl bromoacetate (0.02 mol), and acetone (40 mL) were added to a flask. The mixture was stirred under reflux overnight. The mixture was cooled and then filtered. Acetone was evaporated under reduced pressure. For purification, the residue was crystallized from methanol. The melting points of 6-(4-(4-chlorophenyl/4-fluorophenyl/phenyl)piperazin-1-yl)-3(2*H*)-pyridazinone-2-ylacetates **IIIa–c** were compatible with the literature data [12,27,28].

*Synthesis of ethyl 6-(4-(4-chlorophenyl/4-fluorophenyl/phenyl)piperazin-1-yl)-3(2H)-pyridazinone-2-ylacetohydrazides***IVa**–**c**. Ethyl 6-(4-(4-chlorophenyl/4-fluorophenyl/phenyl)piperazin-1-yl)-3(*2H*)-pyridazinone-2-ylacetate (0.01 mol), hydrazine hydrate (3 mL, 99%) and methanol (25 mL) were added to a flask. The reaction mixture was stirred at 25 °C for 3 h. The product was filtered, washed with distilled water, dried, and crystallized from ethanol. The melting points of 6-(4-(4-chlorophenyl/4-fluorophenyl/phenyl)piperazine-1-yl)-3(2*H*)-pyridazinone-2-yl acetohydrazides (**4a** obtained in 62% yield, **4b** in 71% yield, **4c** in 69% yield) are 228-229, 231-232 and 251-252 °C, respectively. Characterization data of the compounds were compatible with the literature data [12,27,28].

#### 3.1.2. General Synthesis of 6-(4-(4-Chlorophenyl/4-fluorophenyl/phenyl)piperazin-1-yl)pyridazin-3(2*H*)-one-2-acetyl-2-(substituted/nonsubstituted benzal)hydrazones **1**–**23**

A suitable benzaldehyde derivative (0.01 mol) and the appropriate 6-(4-(4-chlorophenyl/4-fluorophenyl/phenyl)piperazin-1-yl]-3(2*H*)-pyridazinone-2-yl acetohydrazide **IVa**–**c** (0.01 mol) were added to ethanol (15 mL). The mixture was stirred under reflux for 6 h. The reaction mixture was transferred into ice water, filtered and dried. The residue was crystallized from methanol:water mixtures. The structures of the compounds were determined by ^1^H-NMR, ^13^C-NMR, IR spectroscopy and elemental analysis. All spectral data of the compounds were in accordance with the assigned structures as given below.

*N′-Benzylidene-2-(6-oxo-3-(4-phenylpiperazin-1-yl)pyridazin-1(6H)-yl)acetohydrazide* (**1**). White crystals (methanol/water); yield 83%; m.p. 236–237 °C; IR (ν cm^−1^, ATR): 3055 (C–H aromatic), 2947 (C–H aliphatic), 1652, 1629 (C=O), 1561 (C=N), 1231 (C–N). ^1^H-NMR (DMSO-*d_6_*, 600 MHz): *δ* 3.22 (4H; t; CH_2_N; Hb + Hb’), 3.38 (4H; t; CH_2_N; Ha + Ha’), 5.07 (2H; s; NCH_2_CO), 6.92 (1H; d; *J* = 4.2 Hz, pyridazinone H^5^), 6.94 (1H; d; *J* = 4.2 Hz, pyridazinone H^4^), 6.98–7.72 (10H; m; phenyl protons), 8.02 (1H; s; –N=CH–), 11.70 (1H; s; -NH–N); ^13^C–NMR (DMSO-*d_6_*, 150 MHz): *δ* 46.5 (2C, CH_2_–N; b + b’), 48.4 (2C, CH_2_–N; a + a’), 53.0 (1C; –N–CH_2_–C=O), 116.2 (1C; =CH), 119.7 (2C; phenyl C^2,6^), 127.6 (1C; phenyl C^4^), 129.4 (1C; =CH-phenyl C^6^), 130.4 (1C; =CH-phenyl C^2^), 130.6 (2C; phenyl C^3,5^), 131.1 (1C; =CH–phenyl C^1^), 134.4 (1C; =CH–phenyl C^3^), 144.3 (2C; =CH–phenyl C^4,5^), 147.4 (1C; pyridazinone C^5^), 149.1 (1C; phenyl C^1^), 151.3 (1C; pyridazinone C^6^), 158.2 (1C; pyridazinone C^4^), 163.8 (1C; CH_2_–N–C=O), 168.5 (1C; pyridazinone C^3^); LC/API-ESMS *m/z* 439 [M + Na]^+^; Anal. Calcd. for C_23_H_24_N_6_O_3_**^.^**1/4 H_2_O: C, 65.62; H, 5.87; N, 19.96. Found: C, 65.96; H, 6.103; N, 20.12%.

*N’-(3-Methylbenzylidene)-2-(6-oxo-3-(4-phenylpiperazin-1-yl)pyridazin-1(6H)-yl)aceto-hydrazide* (**2**). White crystals (methanol/water); yield 80%; m.p. 224–225 °C; IR (ν cm^−1^, ATR): 3052 (C–H aromatic), 2949 (C–H aliphatic), 1652 (C=O), 1581 (C=N), 1267 (C–N). ^1^H-NMR (DMSO-*d_6_*, 600 MHz): *δ* 2.34 (3H; s; CH_3_), 3.28 (4H; t; CH_2_N; Hb + Hb’), 3.38 (4H; t; CH_2_N; Ha + Ha’), 5.07 (2H; s; NCH_2_CO), 6.92 (1H; d; *J* = 4.1 Hz, pyridazinone H^5^), 6.93 (1H; d; *J* = 4.2 Hz, pyridazinone H^4^), 7.22–7.65 (9H; m; phenyl protons), 8.19 (1H; s; –N=CH–), 11.64 (1H; s; –NH–N); ^13^C-NMR (DMSO-*d_6_*, 150 MHz): *δ* 21.4 (1C; CH_3_), 39.6 (2C, CH_2_–N; b + b’), 39.7 (2C, CH_2_–N; a + a’), 53.5 (1C; –N–CH_2_–C=O), 116.2 (1C; =CH), 119.7 (2C; phenyl C^2,6^), 124.9 (1C; phenyl C^4^), 127.7 (1C; 3-methylphenyl C^6^), 129.4 (1C; 3-methylphenyl C^2^), 131.0 (2C; phenyl C^3,5^), 134.4 (1C; 3-methylphenyl C^1^), 134.6 (1C; 3-methylphenyl C^3^), 138.5 (2C; 3-methylphenyl C^4,5^), 138.5 (1C; pyridazinone C^5^), 144.4 (1C; phenyl C^1^), 149.0 (1C; pyridazinone C^6^), 149.1 (1C; pyridazinone C^4^), 163.7 (1C; CH_2_–N–C=O), 168.4 (1C; pyridazinone C^3^); LC/API-ESMS *m/z* 453 [M + Na]^+^; Anal. Calcd. for C_24_H_26_N_6_O_2_**^.^**1/3 H_2_O: C, 66.04; H, 6.16; N, 19.25. Found: C, 66.09; H, 6.10; N, 19.63%.

*N’-(4-Methylbenzylidene)-2-(6-oxo-3-(4-phenylpiperazin-1-yl)pyridazin-1(6H)-yl)aceto-hydrazide* (**3**). White crystals (methanol/water); yield 74%; m.p. 210–212 °C; IR (ν cm^−1^, ATR): 3400 (N–H), 3052 (C–H aromatic), 2949 (C–H aliphatic), 1652 (C=O), 1581 (C=N), 1267 (C–N). ^1^H-NMR (DMSO-*d_6_*, 600 MHz): *δ* 2.34 (3H; s; CH_3_), 3.22 (4H; t; CH_2_N; Hb + Hb’), 3.38 (4H; t; CH_2_N; Ha + Ha’), 5.05 (2H; s; NCH_2_CO), 6.90 (1H; d; *J* = 4.1 Hz, pyridazinone H^5^), 6.93 (1H; d; *J* = 4.2 Hz, pyridazinone H^4^), 7.22–7.65 (9H; m; phenyl protons), 8.18 (1H; s; –N=CH–), 11.59 (1H; s; –NH–N); ^13^C-NMR (DMSO-*d_6_*, 150 MHz): *δ* 21.5 (1C; CH_3_), 46.4 (2C, CH_2_-N; b+b’), 48.4 (2C, CH_2_-N; a+a’), 53.1 (1C; -N-CH_2_-C=O), 116.2 (1C; =CH), 119.8 (2C; phenyl C^2,6^), 126.9 (1C; phenyl C^4^), 127.6 (1C; 4-methylphenyl C^6^), 129.8 (1C; 4-methylphenyl C^2^), 129.9 (2C; phenyl C^3,5^), 131.0 (1C; 4-methylphenyl C^1^), 131.9 (1C; 4-methylphenyl C^3^), 140.2 (2C; 4-methylphenyl C^4,5^), 140.4 (1C; pyridazinone C^5^), 144.3 (1C; phenyl C^1^), 144.4 (1C; pyridazinone C^6^), 149.1 (1C; pyridazinone C^4^), 158.2 (1C; CH_2_–N–C=O), 168.3 (1C; pyridazinone C^3^); LC/API-ESMS *m/z* 453 [M + Na]^+^; Anal. Calcd. for C_24_H_26_N_6_O_2_**^.^**2/3 H_2_O: C, 65.14; H, 6.23; N, 18.99. Found: C, 64.86; H, 5.99; N, 19.30%.

*N’-(3-Methoxybenzylidene)-2-(6-oxo-3-(4-phenylpiperazin-1-yl)pyridazin-1(6H)-yl)acetohydrazide* (**4**). White crystals (methanol/water); yield 68%; m.p. 205–206 °C; IR (ν cm^−1^, ATR): 3389 (N–H), 3094 (C–H aromatic), 2971 (C–H aliphatic), 1681, 1652 (C=O), 1575 (C=N), 1243 (C–N), 1174 (C–O). ^1^H-NMR (DMSO-*d_6_*, 600 MHz): *δ* 3.22 (4H; t; CH_2_N; b + b’), 3.38 (4H; t; CH_2_N; a + a’), 3.80 (3H; s; OCH_3_), 5.04 (2H; s; CH_2_CO), 6.93 (1H; d; *J* = 4.2 Hz, pyridazinone H^5^), 6.98 (1H; d; *J* = 4.2 Hz, pyridazinone H^4^), 7.01–7.96 (9H; m; phenyl protons), 8.16 (1H; s; –N=CH–), 11.51 (1H; s; –NH–N); ^13^C-NMR (DMSO-*d_6_*, 150 MHz): *δ* 46.5 (2C, CH_2_–N; b + b’), 48.4 (2C, CH_2_–N; a + a’), 52.9 (1C; –N–CH_2_-C=O), 55.8 (1C, O–CH_3_), 114.8 (1C; =CH), 116.2 (2C; phenyl C^2,6^), 127.1 (1C; phenyl C^4^), 128.9 (1C; 3-methoxyphenyl C^5,6^), 129.4 (1C; 3-methoxyphenyl C^1^), 131.0 (1C; phenyl C^3,5^), 131.1 (1C; 3-methoxyphenyl C^4^), 144.2 (1C; pyridazinone C^4^), 147.3 (1C; 3-methoxyphenyl C^2^), 149.0 (1C; pyridazinone C^5^), 151.3 (1C; phenyl C^1^), 158.1 (1C; pyridazinone C^6^), 158.2 (1C; 3-methoxyphenyl C^3^), 163.5 (1C; CH_2_–N–C=O), 168.2 (1C; pyridazinone C^3^); LC/API-ESMS *m/z* 469.2 [M + Na]^+^; Anal. Calcd. for C_24_H_26_N_6_O_3_.3/2 H_2_O: C, 60.87; H, 6.17; N, 18.01. Found: C, 61.70; H, 5.74; N, 18.01%.

*N’-(2,3-Dimethoxybenzylidene)-2-(6-oxo-3-(4-phenylpiperazin-1-yl)pyridazin-1(6H)-yl)aceto-hydrazide* (**5**). White crystals (methanol/water); yield 86%; m.p. 249–250 °C; IR (ν cm^−1^, ATR): 3105 (C–H aromatic), 2966 (C–H aliphatic), 1695, 1659 (C=O), 1571 (C=N), 1232 (C–N), 1070 (C–O). ^1^H-NMR (DMSO-*d_6_*, 600 MHz): *δ* 3.22 (4H; t; CH_2_N; Hb + Hb’), 3.38 (4H; t; CH_2_N; Ha + Ha’), 3.78 (3H; s; OCH_3_), 3.84 (3H; s; OCH_3_), 5.05 (2H; s; NCH_2_CO), 6.99 (1H; d; *J* = 4.2 Hz, pyridazinone H^5^), 7.10 (1H; d; *J* = 4.2 Hz, pyridazinone H^4^), 7.12–7.65 (8H; m; phenyl protons), 8.31 (1H; s; –N=CH–), 11.63 (1H; s; –NH–N); ^13^C-NMR (DMSO-*d_6_*, 150 MHz): *δ* 46.5 (2C, CH_2_–N; b + b’), 48.4 (2C, CH_2_–N; a + a’), 52.9 (1C; –N–CH_2_–C=O), 56.2 (1C; –O–CH_3_), 61.7 (1C; –O–CH_3_), 114.6 (1C; =CH), 116.2 (2C; phenyl C^2,6^), 119.7 (1C; phenyl C^4^), 124.8 (1C; 2,3-dimethoxyphenyl C^6^), 127.9 (1C; 2,3-dimethoxyphenyl C^1^), 130.9 (2C; phenyl C^3,5^), 131.1 (2C; 2,3-dimethoxyphenyl C^4,5^), 140.1 (2C; 2,3-dimethoxyphenyl C^3^), 142.9 (1C; 2,3-dimethoxyphenyl C^2^), 148.4 (1C; pyridazinone C^5^), 149.0 (1C; phenyl C^1^), 151.3 (1C; pyridazinone C^6^), 158.2 (1C; pyridazinone C^4^), 163.7 (1C; CH_2_–N–C=O), 168.4 (1C; pyridazinone C^3^); LC/API-ESMS *m/z* 496 [M]^+^; Anal. Calcd. for C_25_H_28_N_6_O_4_**^.^**1/3 H_2_O: C, 62.23; H, 5.99; N, 17.42. Found: C, 62.59; H, 5.77; N, 17.62%.

*N’-(3,5-Dimethoxybenzylidene)-2-(6-oxo-3-(4-phenylpiperazin-1-yl)pyridazin-1(6H)-yl)aceto-hydrazide* (**6**). White crystals (methanol/water); yield 57%; m.p. 232–233 °C; IR (ν cm^−1^, ATR): 3094 (C–H aromatic), 2960 (C–H aliphatic), 1685, 1657 (C=O), 1574 (C=N), 1262 (C–N), 1150 (C–O). ^1^H–NMR (DMSO-*d_6_*, 600 MHz): *δ* 3.22 (4H; t; CH_2_N; Hb + Hb’), 3.38 (4H; t; CH_2_N; Ha + Ha’), 3.78 (6H; s; OCH_3_), 5.10 (2H; s; NCH_2_CO), 6.87 (1H; d; *J* = 4.1 Hz, pyridazinone H^5^), 6.91 (1H; d; *J* = 4.2 Hz, pyridazinone H^4^), 6.99–7.93 (8H; m; phenyl protons), 8.14 (1H; s; –N=CH–), 11.68 (1H; s; –NH–N); ^13^C-NMR (DMSO-*d_6_*, 150 MHz): *δ* 46.5 (2C, CH_2_–N; b + b’), 48.4 (2C, CH_2_–N; a + a’), 53.0 (1C; –N–CH_2_–C=O), 55.8 (2C; –OCH_3_), 105.1 (1C; =CH), 116.2 (2C; phenyl C^2,6^), 119.7 (1C; phenyl C^4^), 127.1 (1C; 3,5-dimethoxyphenyl C^6^), 129.3 (1C; 3,5-dimethoxyphenyl C^2^), 131.0 (1C; phenyl C^1^), 136.5 (1C; 3,5-dimethoxyphenyl C^1^), 136.6 (1C; 3,5-dimethoxyphenyl C^4^), 144.1 (2C; 3,5- dimethoxyphenyl C^3,5^), 147.3 (1C; pyridazinone C^5^), 149.0 (1C; phenyl C^1^), 151.3 (1C; pyridazinone C^6^), 158.2 (1C; pyridazinone C^4^), 163.8 (1C; CH_2_–N–C=O), 168.5 (1C; pyridazinone C^3^); LC/API-ESMS *m/z* 468.8 [M]^+^; Anal. Calcd. for C_25_H_28_N_6_O_4_**^.^**1/3 H_2_O: C, 62.23; H, 5.99; N, 17.42. Found: C, 62.35; H, 5.71; N, 17.57%.

*N’-(2,4,6-Trimethoxybenzylidene)-2-(6-oxo-3-(4-phenylpiperazin-1-yl)pyridazin-1(6H)-yl)aceto-hydrazide* (**7**). White crystals (methanol/water); yield 82%; m.p. 254–255 °C; IR (ν cm^−1^, ATR): 3232 (N–H), 3086 (C–H aromatic), 2933 (C–H aliphatic), 1697, 1670 (C=O), 1595 (C=N), 1239 (C–N), 1126, 1057 (C–O). ^1^H-NMR (DMSO-*d_6_*, 600 MHz): *δ* 3.22 (4H; t; CH_2_N; Hb + Hb’), 3.38 (4H; t; CH_2_N; Ha + Ha’), 3.81 (6H; s; OCH_3_), 3.83 (3H; s; OCH_3_), 4.94 (2H; s; NCH_2_CO), 6.29 (1H; d; *J* = 4.2 Hz, pyridazinone H^5^), 6.94 (1H; d; *J* = 4.2 Hz, pyridazinone H^4^), 6.80–7.64 (8H; m; pyridazinone H^4^ and phenyl protons), 8.19 (1H; s; –N=CH–), 11.26 (1H; s; –NH–N); ^13^C-NMR (DMSO-*d_6_*, 150 MHz): *δ* 46.5 (2C, CH_2_–N; b + b’), 48.4 (2C, CH_2_–N; a + a’), 53.4 (1C; –N–CH_2_–C=O), 55.89 (1C; –OCH_3_), 56.53 (2C; –OCH_3_), 104.3 (1C; =CH), 116.3 (2C; phenyl C^2,6^), 119.7 (1C; phenyl C^4^), 126.9 (1C; 2,4,6-trimethoxyphenyl C^5^), 129.4 (1C; 2,4,6-trimethoxyphenyl C^3^), 131.0 (2C; phenyl C^3,5^), 139.6 (1C; 2,4,6-trimethoxyphenyl C^1^), 143.2 (1C; 2,4,6-trimethoxyphenyl C^4^), 148.9 (2C; 2,4,6-trimethoxyphenyl C^2,6^), 151.3 (1C; pyridazinone C^5^), 158.2 (1C; phenyl C^1^), 158.3 (1C; pyridazinone C^6^), 160.3 (1C; pyridazinone C^4^), 162.7 (1C; CH_2_–N–C=O), 167.9 (1C; pyridazinone C^3^); LC/API-ESMS *m/z* 437 [M-2(OCH_3_)]^+^; Anal. Calcd. for C_26_H_30_N_6_O_5_: C, 61.65; H, 5.97; N, 16.59. Found: C, 61.28; H, 5.82; N, 16.67%.

*N’-(3,4,5-Trimethoxybenzylidene)-2-(6-oxo-3-(4-phenylpiperazin-1-yl)pyridazin-1(6H)-yl)aceto-hydrazide* (**8**). White crystals (methanol/water); yield 83%; m.p. 238–239 °C; IR (ν cm^−1^, ATR): 3216 (N–H), 3065 (C–H aromatic), 2943 (C–H aliphatic), 1669 (C=O), 1578 (C=N), 1231 (C–N), 1124 (C–O). ^1^H-NMR (DMSO-*d_6_*, 600 MHz): *δ* 3.22 (4H; t; CH_2_N; Hb + Hb’), 3.38 (4H; t; CH_2_N; Ha + Ha’), 3.83 (9H; s; OCH_3_), 5.08 (2H; s; NCH_2_CO), 6.82 (1H; d; *J* = 4.1 Hz, pyridazinone H^5^), 6.92 (1H; d; *J* = 4.2 Hz, pyridazinone H^4^), 6.98–7.92 (7H; m; phenyl protons), 8.14 (1H; s; –N=CH–), 11.68 (1H; s; –NH–N); ^13^C-NMR (DMSO-*d_6_*, 150 MHz): *δ* 46.5 (2C, CH_2_–N; b + b’), 48.4 (2C, CH_2_–N; a + a’), 53.5 (1C; –N–CH_2_–C=O), 56.4 (1C; –OCH_3_), 60.6 (2C; –OCH_3_), 104.6 (1C; =CH), 116.3 (2C; phenyl C^2,6^), 119.8 (1C; phenyl C^4^), 127.0 (1C; 3,4,5-trimethoxyphenyl C^6^), 129.4 (1C; 3,4,5-trimethoxyphenyl C^2^), 129.9 (2C; phenyl C^3,5^), 131.0 (1C; 3,4,5-trimethoxyphenyl C^1^), 139.5 (1C; 3,4,5-trimethoxyphenyl C^3^), 144.1 (2C; 3,4,5-trimethoxyphenyl C^4,5^), 147.4 (1C; pyridazinone C^5^), 149.1 (1C; phenyl C^1^), 151.3 (1C; pyridazinone C^6^), 153.6 (1C; pyridazinone C^4^), 163.7 (1C; CH_2_–N–C=O), 168.5 (1C; pyridazinone C^3^); LC/API-ESMS *m/z* 529.2 [M + Na]^+^; Anal. Calcd. for C_26_H_30_N_6_O_5_**^.^**1/2 H_2_O: C, 60.57; H, 6.06; N, 16.30. Found: C, 60.92; H, 5.72; N, 16.55%.

*N’-(4-Nitrobenzylidene)-2-(6-oxo-3-(4-phenylpiperazin-1-yl)pyridazin-1(6H)-yl)acetohydrazide* (**9**). White crystals (methanol/water); yield 54%; m.p. 239–240 °C; IR (ν cm^−1^, ATR): 3089 (C–H aromatic), 2967 (C–H aliphatic), 1692, 1660 (C=O), 1574 (C=N), 1339 (aromatic-NO_2_), 1235 (C–N). ^1^H-NMR (DMSO-*d_6_*, 600 MHz): *δ* 3.23 (4H; t; CH_2_N; Hb + Hb’), 3.39 (4H; t; CH_2_N; Ha + Ha’), 5.11 (2H; s; NCH_2_CO), 6.82 (1H; d; *J* = 4.0 Hz, pyridazinone H^5^), 6.92 (1H; d; *J* = 4.2 Hz, pyridazinone H^4^), 7.22–8.33 (9H; m; phenyl protons), 8.30 (1H; s; –N=CH–), 11.96 (1H; s; –NH–N); ^13^C-NMR (DMSO-*d_6_*, 150 MHz): *δ* 46.5 (2C, CH_2_–N; b + b’), 48.4 (2C, CH_2_–N; a+a’), 53.0 (1C; –N–CH_2_–C=O), 105.1 (1C; =CH), 116.2 (2C; phenyl C^2,6^), 124.4 (1C; phenyl C^4^), 124.5 (1C; 4-nitrophenyl C^6^), 128.3 (1C; 4-nitrophenyl C^2^), 128.4 (2C; phenyl C^3,5^), 129.4 (1C; 4-nitrophenyl C^1^), 140.8 (2C; 4-nitrophenyl C^3,5^), 141.9 (2C; =CH–phenyl C^4^), 142.1 (1C; pyridazinone C^5^), 148.2 (1C; phenyl C^1^), 149.1 (1C; pyridazinone C^6^), 151.3 (1C; pyridazinone C^4^), 158.2 (1C; CH_2_–N–C=O), 168.9 (1C; pyridazinone C^3^); LC/API-ESMS *m/z* 413 [M - NO_2_]^+^; Anal. Calcd. for C_23_H_23_N_7_O_4_**^.^**3/2 H_2_O: C, 56.55; H, 5.36; N, 20.07. Found: C, 56.71; H, 4.99; N, 19.97%.

*N’-(2-Methoxybenzylidene)-2-(6-oxo-3-(4-(4-chlorophenyl)piperazin-1-yl)pyridazin-1(6H)-yl)-acetohydrazide* (**10**). White crystals (methanol/water); yield 80%; m.p. 241–242 °C; IR (ν cm^−1^, ATR): 3236 (N–H), 3074 (C–H aromatic), 2970 (C–H aliphatic), 1683, 1668 (C=O), 1573 (C=N), 1231 (C–N), 1122 (C–O), 837 (C–Cl). ^1^H-NMR (DMSO-*d_6_*, 600 MHz): *δ* 3.23 (4H; t; CH_2_N; b + b’), 3.38 (4H; t; CH_2_N; a + a’), 3.86 (3H; s; OCH_3_), 5.04 (2H; s; CH_2_CO), 7.24 (1H; d; *J* = 4.2 Hz, pyridazinone H^5^), 7.25 (1H; d; *J* = 4.2 Hz, pyridazinone H^4^), 7.26–7.86 (8H; m; phenyl protons), 8.36 (1H; s; –N=CH–), 11.61 (1H; s; –NH–N); ^13^C-NMR (DMSO-*d_6_*, 600 MHz): *δ* 46.3 (2C, CH_2_–N; b + b’), 48.2 (2C, CH_2_–N; a + a’), 53.0 (1C; –N–CH_2_–C=O), 56.1 (1C; –OCH_3_), 112.3 (1C; =CH), 117.7 (2C; 4-chlorophenyl C^2,6^), 121.2 (1C; 2-methoxyphenyl C^6^), 122.4 (1C; 2-methoxyphenyl C^5^), 123.2 (2C; 4-chlorophenyl C^3,5^), 125.9 (2C; 2-methoxyphenyl C^3,4^), 127.1 (1C; pyridazinone C^5^), 129.1 (1C; 4-chlorophenyl C^1^), 131.1 (1C; 2-methoxyphenyl C^1^), 139.9 (1C; 2-methoxyphenyl C^2^), 142.9 (1C; 4-chlorophenyl C^4^), 148.9 (1C; pyridazinone C^4^), 150.1 (1C; pyridazinone C^6^), 163.6 (1C; CH_2_–N–C=O), 166.3 (1C; pyridazinone C^3^); LC/MSMS (ESI+) *m/z* 503 [M + Na]^+^; Anal. Calcd for C_24_H_25_ClN_6_O_3_**^.^**1/5H_2_O: C, 59.49; H, 5.28; N, 17.34. Found: C, 59.13; H, 5.21; N, 17.93%.

*N’-(3-Methoxybenzylidene)-2-(6-oxo-3-(4-(4-chlorophenyl)piperazin-1-yl)pyridazin-1(6H)-yl)-acetohydrazide* (**11**). White crystals (methanol/water); yield 82%; m.p. 214–215 °C; IR (ν cm^−1^, ATR): 3065 (C–H aromatic), 2943 (C–H aliphatic), 1696, 1656 (C=O), 1567 (C=N), 1231 (C–N), 1124 (C–O), 836 (C–Cl). ^1^H-NMR (DMSO-*d_6_*, 600 MHz): *δ* 3.23 (4H; t; CH_2_N; b + b’), 3.38 (4H; t; CH_2_N; a + a’), 3.80 (3H; s; OCH_3_), 5.06 (2H; s; CH_2_CO), 6.99 (1H; d; *J* = 4.2 Hz, pyridazinone H^5^), 7.00–7.98 (9H; m; phenyl protons and pyridazinone H^4^), 8.18 (1H; s; –N=CH–), 11.67 (1H; s; –NH–N); ^13^C-NMR (DMSO-*d_6_*, 150 MHz): *δ* 46.3 (2C, CH_2_–N; b + b’), 48.1 (2C, CH_2_–N; a + a’), 53.0 (1C; –N–CH_2_–C=O), 55.6 (1C; –OCH_3_), 111.6 (1C; =CH), 116.6 (2C; 4-chlorophenyl C^2,6^), 117.7 (1C; 3-methoxyphenyl C^6^), 123.2 (1C; 3-methoxyphenyl C^2^), 127.1 (2C; 4-chlorophenyl C^3,5^), 129.1 (2C; 3-methoxyphenyl C^4,5^), 130.4 (1C; pyridazinone C^5^), 131.0 (1C; 4-chlorophenyl C^1^), 135.9 (1C; 3-methoxyphenyl C^1^), 144.1 (1C; 3-methoxyphenyl C^3^), 148.9 (1C; 4-chlorophenyl C^4^), 150.1 (1C; pyridazinone C^4^), 158.2 (1C; pyridazinone C^6^), 160.0 (1C; CH_2_–N–C=O), 168.5 (1C; pyridazinone C^3^); LC/MSMS (ESI+) *m/z* 414 [M-(Cl + OCH_3_)]^+^; Anal. Calcd for C_24_H_25_ClN_6_O_3_**^.^**1/9H_2_O: C, 59.69; H, 5.26; N, 17.40. Found: C, 59.48; H, 5.12; N, 18.23%.

*N’-(2,3-Dimethoxybenzylidene)-2-(6-oxo-3-(4-(4-chlorophenyl)piperazin-1-yl)pyridazin-1(6H)-yl)acetohydrazide* (**12**). White crystals (methanol/water); yield 80%; m.p. 252–253 °C; IR (ν cm^−1^, ATR): 3196 (N–H), 3045 (C–H aromatic), 2968 (C–H aliphatic), 1690, 1667 (C=O), 1561 (C=N), 1230 (C–N), 1065 (C–O), 843 (C–Cl). ^1^H-NMR (DMSO-*d_6_*, 600 MHz): *δ* 3.23 (4H; t; CH_2_N; Hb + Hb’), 3.37 (4H; t; CH_2_N; Ha + Ha’), 3.78 (3H; s; OCH_3_), 3.84 (3H; s; OCH_3_), 5.04 (2H; s; NCH_2_CO), 6.92 (1H; d; *J* = 4.2 Hz, pyridazinone H^5^), 6.99 (1H; d; *J* = 4.2 Hz, pyridazinone H^4^), 7.10–7.65 (7H; m; phenyl protons), 8.30 (1H; s; –N=CH–), 11.62 (1H; s; –NH–N); ^13^C-NMR (DMSO-*d_6_*, 150 MHz): *δ* 46.3 (2C, CH_2_–N; b + b’), 48.1 (2C, CH_2_–N; a + a’), 52.9 (1C; –N–CH_2_–C=O), 56.2 (1C; –OCH_3_), 61.7 (1C; –OCH_3_), 114.6 (1C; =CH), 117.4 (2C; 4-chlorophenyl C^2,6^), 123.2 (2C; 4-chlorophenyl C^3,5^), 127.9 (1C; 2,3-dimethoxyphenyl C^6^), 129.1 (1C; 2,3-dimethoxyphenyl C^1^), 131.0 (1C; 4-chlorophenyl C^1^), 140.1 (2C; 2,3-dimethoxyphenyl C^4,5^), 148.3 (1C; 2,3-dimethoxyphenyl C^2^), 148.4 (1C; 2,3-dimethoxyphenyl C^3^), 148.9 (1C; pyridazinone C^5^), 150.1 (1C; 4-chlorophenyl C^4^), 153.1 (1C; pyridazinone C^6^), 158.2 (1C; pyridazinone C^4^), 163.7 (1C; CH_2_–N–C=O), 168.4 (1C; pyridazinone C^3^); LC/API-ESMS *m/z* 533 [M + Na]^+^; Anal. Calcd. for C_25_H_27_ClN_6_O_4_**^.^**1/9 H_2_O: C, 58.53; H, 5.35; N, 16.38. Found: C, 58.15; H, 5.16; N, 17.30%.

*N’-(2,4,6-Trimethoxybenzylidene)-2-(6-oxo-3-(4-(4-chlorophenyl)piperazin-1-yl)pyridazin-1(6H)-yl)acetohydrazide* (**13**). White crystals (methanol/water); yield 86%; m.p. 281–282 °C; IR (ν cm^−1^, ATR): 3231 (N–H), 3086 (C–H aromatic), 2933 (C–H aliphatic), 1697, 1668 (C=O), 1592 (C=N), 1236 (C–N), 1156 (C–O), 851 (C–Cl). ^1^H-NMR (DMSO-*d_6_*, 600 MHz): *δ* 3.22 (4H; t; CH_2_N; Hb + Hb’), 3.38 (4H; t; CH_2_N; Ha + Ha’), 3.81 (6H; s; OCH_3_), 3.83 (3H; s; OCH_3_), 4.93 (2H; s; NCH_2_CO), 6.29 (1H; d; *J* = 4.1 Hz, pyridazinone H^5^), 6.89 (1H; d; *J* = 4.2 Hz, pyridazinone H^4^), 6.91–7.64 (6H; m; phenyl protons), 8.18 (1H; s; –N=CH–), 11.25 (1H; s; –NH–N); ^13^C-NMR (DMSO-*d_6_*, 150 MHz): *δ* 46.4 (2C, CH_2_–N; b + b’), 48.1 (2C, CH_2_–N; a + a’), 52.9 (1C; –N–CH_2_–C=O), 55.9 (1C; –OCH_3_), 56.5 (2C; –OCH_3_), 104.3 (1C; =CH), 117.7 (2C; 4-chlorophenyl C^2,6^), 123.2 (2C; 2,4,6-trimethoxyphenyl C^3,5^), 126.9 (1C; 4-chlorophenyl C^3^), 129.1 (1C; 4-chlorophenyl C^5^), 131.0 (1C; 4-chlorophenyl C^1^), 139.6 (1C; 2,4,6-trimethoxyphenyl C^1^), 143.2 (1C; 2,4,6-trimethoxyphenyl C^2,4^), 148.9 (2C; 2,4,6-trimethoxyphenyl C^6^), 150.1 (1C; pyridazinone C^5^), 158.3 (1C; 4-chlorophenyl C^4^), 160.3 (1C; pyridazinone C^6^), 160.4 (1C; pyridazinone C^4^), 162.7 (1C; CH_2_–N–C=O), 167.9 (1C; pyridazinone C^3^); LC/API-ESMS *m/z* 563 [M + Na]^+^; Anal. Calcd. for C_26_H_29_ClN_6_O_5_**^.^**1/6 H_2_O: C, 57.40; H, 5.43; N, 15.45. Found: C, 57.01; H, 5.23; N, 16.47%.

*N’-(3,4,5-Trimethoxybenzylidene)-2-(6-oxo-3-(4-(4-chlorophenyl)piperazin-1-yl)pyridazin-1(6H)-yl)acetohydrazide* (**14**). White crystals (methanol/water); yield 77%; m.p. 285–286 °C; IR (ν cm^−1^, ATR): 3213 (N–H), 3062 (C–H aromatic), 2938 (C–H aliphatic), 1681, 1663 (C=O), 1595 (C=N), 1232 (C–N), 1157 (C–O), 851 (C–Cl). ^1^H-NMR (DMSO-*d_6_*, 600 MHz): *δ* 3.23 (4H; t; CH_2_N; Hb + Hb’), 3.36 (4H; t; CH_2_N; Ha + Ha’), 3.70 (6H; s; OCH_3_), 3.83 (3H; s; OCH_3_), 5.07 (2H; s; NCH_2_CO), 6.91 (1H; d; *J* = 4.2 Hz, pyridazinone H^5^), 6.93-7.92 (7H; m; phenyl protons and pyridazinone H^4^), 8.14 (1H; s; –N=CH–), 11.67 (1H; s; –NH–N); ^13^C-NMR (DMSO-*d_6_*, 150 MHz): *δ* 46.4 (2C, CH_2_–N; b + b’), 48.1 (2C, CH_2_–N; a + a’), 53.1 (1C; –N–CH_2_–C=O), 56.5 (1C; –OCH_3_), 60.5 (2C; –OCH_3_), 104.6 (1C; =CH), 117.7 (2C; 4-chlorophenyl C^2,6^), 119.8 (2C; 2,4,6-trimethoxyphenyl C^2,6^), 123.2 (1C; 4-chlorophenyl C^3^), 129.1 (1C; 4-chlorophenyl C^5^), 129.9 (1C; 4-chlorophenyl C^1^), 131.1 (1C; 2,4,6-trimethoxyphenyl C^1^), 139.5 (1C; 2,4,6-trimethoxyphenyl C^3,5^), 144.0 (2C; 2,4,6-trimethoxyphenyl C^4^), 148.9 (1C; pyridazinone C^5^), 150.1 (1C; 4-chlorophenyl C^4^), 153.6 (1C; pyridazinone C^6^), 158.2 (1C; pyridazinone C^4^), 163.7 (1C; CH_2_–N–C=O), 168.5 (1C; pyridazinone C^3^); LC/API-ESMS *m/z* 560 [M]^+^; Anal. Calcd. for C_26_H_29_ClN_6_O_5_**^.^**1/4 H_2_O: C, 57.25; H, 5.45; N, 15.41. Found: C, 56.86; H, 5.20; N, 16.63%.

*N’-(4-Nitrobenzylidene)-2-(6-oxo-3-(4-(4-chlorophenyl)piperazin-1-yl)pyridazin-1(6H)-yl)acetohydrazide* (**15**). White crystals (methanol/water); yield 86%; m.p. 238–239 °C; IR (ν cm^−1^, ATR): 3077 (C–H aromatic), 2964 (C–H aliphatic), 1689, 1654 (C=O), 1580 (C=N), 1340 (aromatic NO_2_), 1236 (C–N), 836 (C–Cl). ^1^H-NMR (DMSO-*d_6_*, 600 MHz): *δ* 3.23 (4H; t; CH_2_N; b + b’), 3.38 (4H; t; CH_2_N; a + a’), 5.10 (2H; s; CH_2_CO), 6.92 (1H; d; *J* = 4.2 Hz, pyridazinone H^5^), 6.99 (1H; d; *J* = 4.2 Hz, pyridazinone H^5^), 7.24–8.29 (8H; m; phenyl protons), 8.33 (1H; s; –N=CH–), 11.96 (1H; s; –NH–N); ^13^C-NMR (DMSO-*d_6_*, 150 MHz): *δ* 46.3 (2C, CH_2_–N; b + b’), 48.1 (2C, CH_2_-N; a + a’), 53.0 (1C; –N–CH_2_–C=O), 105.1 (1C; =CH), 117.7 (2C; 4-chlorophenyl C^2,6^), 124.5 (1C; 4-nitrophenyl C^6^), 127.1 (1C; 4-nitrophenyl C^2^), 128.3 (2C; 4-chlorophenyl C^3,5^), 129.1 (2C; 4-nitrophenyl C^3,5^), 131.0 (1C; pyridazinone C^5^), 141.9 (1C; 4-chlorophenyl C^1^), 145.0 (1C; 4-nitrophenyl C^1^), 148.2 (1C; 4-nitrophenyl C^4^), 149.0 (1C; 4-chlorophenyl C^4^), 150.1 (1C; pyridazinone C^4^), 158.2 (1C; pyridazinone C^6^), 164.3 (1C; CH_2_–N–C=O), 168.9 (1C; pyridazinone C^3^); LC/MSMS (ESI+) *m/z* 414 [M-(Cl + NO_2_)]^+^; Anal. Calcd for C_23_H_22_ClN_7_O_4_**^.^**1/9H_2_O: C, 55.48; H, 4.50; N, 19.69. Found: C, 55.19; H, 4.42; N, 20.76%.

*N’-(4-Dimethylaminobenzylidene)-2-(6-oxo-3-(4-(4-chlorophenyl)piperazin-1-yl)pyridazin-1(6H)-yl)acetohydrazide* (**16**). White crystals (methanol/water); yield 68%; m.p. 243–244 °C; IR (ν cm^−1^, ATR): 3082 (C–H aromatic), 2962 (C–H aliphatic), 1678, 1665 (C=O), 1589 (C=N), 1227 (C–N), 836 (C–Cl). ^1^H-NMR (DMSO-*d_6_*, 600 MHz): *δ* 2.96 (6H; s; –N(CH_3_)_2_), 3.23 (4H; t; CH_2_N; b + b’), 3.37 (4H; t; CH_2_N; a + a’), 5.01 (2H; s; CH_2_CO), 6.72 (1H; d; *J* = 4.1 Hz, pyridazinone H^5^), 6.74 (1H; d; *J* = 4.2 Hz, pyridazinone H^5^), 6.91–7.88 (8H; m; phenyl protons), 8.06 (1H; s; –N=CH–), 11.36 (1H; s; –NH–N); ^13^C-NMR (DMSO-*d_6_*, 150 MHz): *δ* 46.4 (2C, CH_2_–N; b + b’), 46.5 (2C; –N(CH_3_)_2_), 48.1 (2C, CH_2_–N; a+a’), 52.9 (1C; –N–CH_2_–C=O), 112.3 (1C; =CH), 117.7 (2C; 4-chlorophenyl C^2,6^), 121.8 (1C; 4-dimethylaminophenyl C^6^), 127.1 (1C; 4-dimethylaminophenyl C^2^), 128.9 (2C; 4-chlorophenyl C^3,5^), 129.1 (2C; 4-dimethylaminophenyl C^3,5^), 131.1 (1C; pyridazinone C^5^), 145.1 (1C; 4-chlorophenyl C^1^), 148.2 (1C; 4-dimethylphenyl C^1^), 149.0 (1C; 4-dimethylphenyl C^4^), 150.1 (1C; 4-chlorophenyl C^4^), 151.9 (1C; pyridazinone C^4^), 158.2 (1C; pyridazinone C^6^), 163.1 (1C; CH_2_–N–C=O), 167.8 (1C; pyridazinone C^3^); LC/MSMS (ESI+) *m/z* 516 [M + Na]^+^; Anal. Calcd for C_25_H_28_ClN_7_O_2_**^.^**1/6 H_2_O: C, 60.42; H, 5.75; N, 19.73. Found: C, 60.03; H, 5.55; N, 20.48%.

*N’-Benzylidene-2-(6-oxo-3-(4-(4-fluorophenyl)piperazin-1-yl)pyridazin-1(6H)-yl)acetohydrazide* (**17**). White crystals (methanol/water); yield 47%; m.p. 207–208 °C; IR (ν cm^−1^, ATR): 3178 (N–H), 3056 (C–H aromatic), 2960 (C–H aliphatic), 1652 (C=O), 1585 (C=N), 1222 (C–N), 837 (C–F). ^1^H-NMR (DMSO-*d_6_*, 600 MHz): *δ* 3.17 (4H; t; CH_2_N; b + b’), 3.38 (4H; t; CH_2_N; a + a’), 5.06 (2H; s; CH_2_CO), 7.01 (1H; d; *J* = 4.2 Hz, pyridazinone H^5^), 7.02 (1H; d; *J* = 4.2 Hz, pyridazinone H^4^), 7.06-7.72 (9H; m; phenyl protons), 8.26 (1H; s; –N=CH–), 11.66 (1H; s; –NH–N); ^13^C-NMR (DMSO-*d_6_*, 150 MHz): *δ* 46.5 (2C, CH_2_–N; b + b’), 49.1 (2C, CH_2_–N; a + a’), 52.9 (1C; –N–CH_2_-C=O), 115.7 (1C; =CH), 118.1 (2C; phenyl C^4^), 127.2 (1C; phenyl C^3,5^), 129.3 (1C; phenyl C^2,6^), 130.4 (2C; 4-fluorophenyl C^3,5^), 131.0 (2C; 4-fluorophenyl C^2,6^), 134.4 (1C; 4-fluorophenyl C^3,5^), 144.3 (1C; 4-fluorophenyl C^1^), 147.4 (1C; phenyl C^1^), 148.2 (1C; pyridazinone C^5^), 149.1 (1C; 4-fluorophenyl C^4^), 155.9 (1C; pyridazinone C^4^), 158.2 (1C; pyridazinone C^6^), 163.8 (1C; CH_2_–N–C=O), 168.5 (1C; pyridazinone C^3^); LC/MSMS (ESI+) *m/z* 457 [M + Na]^+^; Anal. Calcd for C_23_H_23_FN_6_O_2_**^.^**1/2H_2_O: C, 62.29; H, 5.45; N, 18.95. Found: C, 62.21; H, 5.32; N, 18.81%.

*N’-(3-M-ethylbenzylidene)-2-(6-oxo-3-(4-(4-fluorophenyl)piperazin-1-yl)pyridazin-1(6H)-yl)acetohydrazide* (**18**). White crystals (methanol/water); yield 51%; m.p. 201–202 °C; IR (ν cm^−1^, ATR): 3170 (N–H), 3045 (C–H aromatic), 2988 (C–H aliphatic), 1652 (C=O), 1576 (C=N), 1264 (C–N). ^1^H-NMR (DMSO-*d_6_*, 600 MHz): *δ* 2.51 (3H; s; CH_3_), 3.17 (4H; t; CH_2_N; Hb+Hb’), 3.38 (4H; t; CH_2_N; Ha + Ha’), 5.07 (2H; s; NCH_2_CO), 7.00 (1H; d; *J* = 4.2 Hz, pyridazinone H^5^), 7.02–7.98 (9H; m; phenyl protons and pyridazinone H^5^), 8.19 (1H; s; –N=CH–), 11.67 (1H; s; –NH–N); ^13^C-NMR (DMSO-*d_6_*, 150 MHz): *δ* 21.4 (1C; CH_3_), 46.5 (2C, CH_2_–N; b + b’), 49.1 (2C, CH_2_–N; a + a’), 55.6 (1C; –N–CH_2_–C=O), 111.6 (1C; =CH), 115.9 (2C; 4-fluorophenyl C^2,6^), 118.0 (2C; 4-fluorophenyl C^3,5^), 120.2 (1C; 3-methylphenyl C^6^), 130.4 (1C; 3-methylphenyl C^2^), 131.0 (1C; 4-fluorophenyl C^1^), 135.9 (1C; 3-methylphenyl C^1^), 144.1 (1C; 3-methylphenyl C^3^), 147.3 (2C; 3-methylphenyl C^4,5^), 148.2 (1C; pyridazinone C^5^), 149.0 (1C; 4-fluorophenyl C^4^), 155.9 (1C; pyridazinone C^6^), 158.2 (1C; pyridazinone C^4^), 163.8 (1C; CH_2_–N–C=O), 168.5 (1C; pyridazinone C^3^); LC/API-ESMS *m/z* 414 [M-(F + CH_3_)]^+^; Anal. Calcd. for C_24_H_25_FN_6_O_2_**^.^**4/5 H_2_O: C, 61.20; H, 5.80; N, 17.84. Found: C, 60.85; H, 5.40; N, 17.84%.

*N’-(3-Methoxybenzylidene)-2-(6-oxo-3-(4-(4-fluorophenyl)piperazin-1-yl)pyridazin-1(6H)-yl)acetohydrazide* (**19**). White crystals (methanol/water); yield 48%; m.p. 202–203 °C; IR (ν cm^−1^, ATR): 3069 (C–H aromatic), 2960 (C–H aliphatic), 1698, 1656 (C=O), 1569 (C=N), 1234 (C–N), 1158 (C–O), 836 (C–F). ^1^H-NMR (DMSO-*d_6_*, 600 MHz): *δ* 3.17 (4H; t; CH_2_N; b + b’), 3.38 (4H; t; CH_2_N; a + a’), 3.80 (3H; s; OCH_3_), 5.06 (2H; s; CH_2_CO), 7.00 (1H; d; *J* = 4.2 Hz, pyridazinone H^5^), 7.01–7.98 (9H; m; phenyl protons and pyridazinone H^4^), 8.19 (1H; s; –N=CH–), 11.67 (1H; s; –NH–N); ^13^C-NMR (DMSO-*d_6_*, 150 MHz): *δ* 46.5 (2C, CH_2_–N; b + b’), 49.1 (2C, CH_2_–N; a + a’), 53.0 (1C; –N–CH_2_–C=O), 55.6 (1C; –OCH_3_), 111.6 (1C; =CH), 115.6 (2C; 4-fluorophenyl C^2,6^), 118.1 (1C; 3-methoxyphenyl C^6^), 120.2 (1C; 3-methoxyphenyl C^2^), 130.4 (2C; 4-fluorophenyl C^3,5^), 131.0 (2C; 3-methoxyphenyl C^4,5^), 135.9 (1C; pyridazinone C^5^), 144.1 (1C; 4-fluorophenyl C^1^), 147.3 (1C; 3-methoxyphenyl C^1^), 149.0 (1C; 3-methoxyphenyl C^3^), 155.9 (1C; 4-fluorophenyl C^4^), 157.5 (1C; pyridazinone C^4^), 158.2 (1C; pyridazinone C^6^), 160.0 (1C; CH_2_–N–C=O), 168.5 (1C; pyridazinone C^3^); LC/MSMS (ESI+) *m/z* 487 [M + Na]^+^; Anal. Calcd for C_24_H_25_FN_6_O_3_**^.^**2/3H_2_O: C, 60.49; H, 5.57; N, 17.64. Found: C, 60.06; H, 5.13; N, 17.43%.

*N’-(4-Methoxybenzylidene)-2-(6-oxo-3-(4-(4-chlorophenyl)piperazin-1-yl)pyridazin-1(6H)-yl)acetohydrazide* (**20**). White crystals (methanol/water); yield 41%; m.p. 247–248 °C; IR (ν cm^−1^, ATR): 3065 (C–H aromatic), 2966 (C–H aliphatic), 1686 (C=O), 1570 (C=N), 1247 (C–N), 1024 (C–O), 836 (C–F). ^1^H-NMR (DMSO-*d_6_*, 600 MHz): *δ* 3.17 (4H; t; CH_2_N; b + b’), 3.38 (4H; t; CH_2_N; a + a’), 3.80 (3H; s; OCH_3_), 5.04 (2H; s; CH_2_CO), 6.91 (1H; d; *J* = 4.1 Hz, pyridazinone H^5^), 6.92-7.96 (9H; m; phenyl protons and pyridazinone H^4^), 8.16 (1H; s; –N=CH–), 11.52 (1H; s; –NH–N); ^13^C-NMR (DMSO-*d_6_*, 150 MHz): *δ* 46.5 (2C, CH_2_–N; b + b’), 49.1 (2C, CH_2_–N; a + a’), 52.9 (1C; –N–CH_2_–C=O), 55.8 (1C; –OCH_3_), 114.8 (1C; =CH), 115.9 (2C; 4-fluorophenyl C^2,6^), 118.1 (1C; 4-methoxyphenyl C^6^), 127.0 (1C; 4-methoxyphenyl C^2^), 128.9 (2C; 4-fluorophenyl C^3,5^), 129.2 (2C; 4-methoxyphenyl C^3,5^), 131.0 (1C; pyridazinone C^5^), 144.1 (1C; 4-fluorophenyl C^1^), 147.3 (1C; 4-methoxyphenyl C^1^), 148.2 (1C; 4-methoxyphenyl C^4^), 149.1 (1C; 4-fluorophenyl C^4^), 155.9 (1C; pyridazinone C^4^), 158.2 (1C; pyridazinone C^6^), 163.5 (1C; CH_2_–N–C=O), 168.2 (1C; pyridazinone C^3^); LC/MSMS (ESI+) *m/z* 487 [M + Na]^+^; Anal. Calcd for C_24_H_25_FN_6_O_3_**^.^**2/3H_2_O: C, 60.49; H, 5.57; N, 17.64. Found: C, 60.05; H, 5.24; N, 17.50%.

*N’-(2,4,6-Trimethoxybenzylidene)-2-(6-oxo-3-(4-(4-fluorophenyl)piperazin-1-yl)pyridazin-1(6H)-yl)acetohydrazide* (**21**). White crystals (methanol/water); yield 44%; m.p. 275–276 ^°^C; IR (ν cm^−1^, ATR): 3231 (N–H), 3085 (C–H aromatic), 2963 (C–H aliphatic), 1673, 1668 (C=O), 1595 (C=N), 1240 (C–N), 1128 (C–O), 851 (C–F). ^1^H-NMR (DMSO-*d_6_*, 600 MHz): *δ* 3.18 (4H; t; CH_2_N; Hb + Hb’), 3.37 (4H; t; CH_2_N; Ha + Ha’), 3.82 (6H; s; OCH_3_), 3.83 (3H; s; OCH_3_), 4.94 (2H; s; NCH_2_CO), 6.29 (1H; d; *J* = 4.2 Hz, pyridazinone H^5^), 6.90 (1H; d; *J* = 4.2 Hz, pyridazinone H^4^), 6.91–7.64 (6H; m; phenyl protons), 8.19 (1H; s; –N=CH–), 11.26 (1H; s; –NH–N); ^13^C-NMR (DMSO-*d_6_*, 150 MHz): *δ* 46.5 (2C, CH_2_–N; b + b’), 49.2 (2C, CH_2_–N; a + a’), 52.9 (1C; –N–CH_2_–C=O), 55.9 (2C; –OCH_3_), 56.5 (2C; –OCH_3_), 104.2 (1C; =CH), 115.9 (2C; 4-fluorophenyl C^2,6^), 118.1 (2C; 2,4,6-trimethoxyphenyl C^3,5^), 126.9 (1C; 4-fluorophenyl C^3^), 127.0 (1C; 4-fluorophenyl C^5^), 131.0 (1C; 4-fluorophenyl C^1^), 139.6 (1C; 2,4,6-trimethoxyphenyl C^1^), 148.2 (3C; 2,4,6-trimethoxyphenyl C^2,4,6^), 148.9 (1C; pyridazinone C^5^), 155.9(1C; 4-fluorophenyl C^4^), 158.3 (1C; pyridazinone C^6^), 160.4 (1C; pyridazinone C^4^), 162.6 (1C; CH_2_–N–C=O) and 167.9 (1C; pyridazinone C^3^); LC/API-ESMS *m/z* 524 [M]^+^; Anal. Calcd. for C_26_H_29_FN_6_O_5_.H_2_O: C, 57.56; H, 5.76; N, 15.49. Found: C, 57.75; H, 5.30; N, 15.64%.

*N’-(4-Nitrobenzylidene)-2-(6-oxo-3-(4-(4-fluorophenyl)piperazin-1-yl)pyridazin-1(6H)-yl)acetohydrazide* (**22**). White crystals (methanol/water); yield 49%; m.p. 243–244 °C; IR (ν cm^−1^, ATR): 3077 (C–H aromatic), 2963 (C–H aliphatic), 1690, 1659 (C=O), 1580 (C=N), 1339 (aromatic NO_2_), 1236 (C–N), 836 (C–F). ^1^H-NMR (DMSO-*d_6_*, 600 MHz): *δ* 3.17 (4H; t; CH_2_N; b + b’), 3.39 (4H; t; CH_2_N; a + a’), 5.11 (2H; s; NCH_2_CO), 6.94 (1H; d; *J* = 4.2 Hz, pyridazinone H^5^), 6.99 (1H; d; *J* = 4.2 Hz, pyridazinone H^5^), 7.00-8.39 (8H; m; phenyl protons), 8.88 (1H; s; –N=CH–), 11.96 (1H; s; –NH–N); ^13^C-NMR (DMSO-*d_6_*, 150 MHz): *δ* 46.5 (2C, CH_2_–N; b + b’), 49.1 (2C, CH_2_–N; a+a’), 53.0 (1C; –N–CH_2_–C=O), 115.9 (1C; =CH), 118.0 (2C; 4-fluorophenyl C^2,6^), 124.5 (1C; 4-nitrophenyl C^6^), 127.1 (1C; 4-nitrophenyl C^2^), 128.4 (2C; 4-fluorophenyl C^3,5^), 128.5 (2C; 4-nitrophenyl C^3,5^), 131.0 (1C; pyridazinone C^5^), 140.8 (1C; 4-fluorophenyl C^1^), 141.9 (1C; 4-nitrophenyl C^1^), 148.2 (1C; 4-nitrophenyl C^4^), 149.1 (1C; 4-fluorophenyl C^4^), 155.9 (1C; pyridazinone C^4^), 157.5 (1C; pyridazinone C^6^), 158.2 (1C; CH_2_–N–C=O), 168.9 (1C; pyridazinone C^3^); LC/MSMS (ESI+) *m/z* 414 [M-(F + NO_2_)]^+^; Anal. Calcd for C_23_H_22_FN_7_O_4_.2/3H_2_O: C, 56.21; H, 4.79; N, 19.95. Found: C, 56.07; H, 4.89; N, 19.63%.

*N’-(4-dimethylaminobenzylidene)-2-(6-oxo-3-(4-(4-fluorophenyl)piperazin-1-yl)pyridazin-1(6H)-yl)acetohydrazide* (**23**). White crystals (methanol/water); yield 45%; m.p. 248–249 °C; IR (ν cm^−1^, ATR): 3046 (C–H aromatic), 2994 (C–H aliphatic), 1646, 1612 (C=O), 1584 (C=N), 1265 (C–N), 846 (C–F). ^1^H-NMR (DMSO-*d_6_*, 600 MHz): *δ* 2.97 (6H; s; –N(CH_3_)_2_), 3.17 (4H; t; CH_2_N; b + b’), 3.38 (4H; t; CH_2_N; a + a’), 5.01 (2H; s; CH_2_CO), 6.75 (1H; d; *J* = 4.1 Hz, pyridazinone H^5^), 6.77 (1H; d; *J* = 4.2 Hz, pyridazinone H^5^), 6.91–7.88 (8H; m; phenyl protons), 8.06 (1H; s; –N=CH–), 11.35 (1H; s; –NH–N); ^13^C-NMR (DMSO-*d_6_*, 150 MHz): *δ* 46.5 (2C, CH_2_–N; b + b’), 48.4 (2C; –N(CH_3_)_2_), 49.1 (2C, CH_2_–N; a+a’), 52.9 (1C; –N–CH_2_–C=O), 112.3 (1C; =CH), 115.9 (2C; 4-fluorophenyl C^2,6^), 118.1 (1C; 4-dimethylaminophenyl C^6^), 121.8 (1C; 4-dimethylaminophenyl C^2^), 126.9 (2C; 4-fluorophenyl C^3,5^), 128.9 (2C; 4-dimethylaminophenyl C^3,5^), 129.9 (1C; pyridazinone C^5^), 145.1 (1C; 4-fluorophenyl C^1^), 148.2 (1C; 4-dimethylphenyl C^1^), 151.9 (1C; 4-dimethylphenyl C^4^), 155.9 (1C; 4-fluorophenyl C^4^), 157.5 (1C; pyridazinone C^4^), 158.2 (1C; pyridazinone C^6^), 163.1 (1C; CH_2_–N–C=O), 167.8 (1C; pyridazinone C^3^); LC/MSMS (ESI+) *m/z* 501 [M + Na]^+^; Anal. Calcd for C_25_H_28_FN_7_O_2_.1/3 H_2_O: C, 62.10; H, 5.98; N, 20.28. Found: C, 62.24; H, 5.80; N, 20.37%.

### 3.2. Cell Culture

#### 3.2.1. Human Gingival Fibroblasts (HGFs) Culture

A total of 10 donors, periodontally and systemically healthy, subjected to the extraction of the third molar, signed the informed consent according to the Italian Legislation and in accordance with the code of Ethical Principles for Medical Research comprising Human Subjects of the World Medical Association (Declaration of Helsinki). The project obtained the approval of the Local Ethical Committee of the University of Chieti (Chieti, Italy; approval number. 1173, approved on 31 March 2016). The tissue samples were immediately placed in Dulbecco’s modified Eagle’s medium (DMEM) then rinsed in phosphate-buffered saline buffer (PBS), cut into smaller fragments and cultured in DMEM, supplemented with 10% foetal bovine serum (FBS), 1% penicillin/ streptomycin and 1% fungizone (all purchased from EuroClone, Milan, Italy). Cells were kept at 37 °C in a humidified atmosphere of 5% (*v*/*v*) CO_2_. After 10 days of culture fungizone was removed and cells used after 7–14 passages, as previously reported [35].

#### 3.2.2. AGS Culture

AGS human gastric adenocarcinoma cell line (ECACC 89090402, Sigma Aldrich, Milan, Italy) was cultured in Ham’s F12 medium supplemented with 10% of FBS, 1% of penicillin/streptomycin, and 1% of L-glutamine (all purchased from EuroClone). Cell culture was kept at 37 °C in a humidified atmosphere with 5% CO_2_.

### 3.3. HGF and AGS Treatment

For each tested compound a stock solution 0.1 M in DMSO was prepared. The stock solution was then diluted in DMEM or Ham’s F12 medium (for HGFs and AGS, respectively) to obtain final solutions of 10 µM for HGFs and of 10 µM and 50 µM for AGS cells. Doxorubicin was used as a reference drug. To exclude DMSO cytotoxicity, in all the incubation media, the final concentration of DMSO was maintained at 0.2%.

The HGFs and AGS cells were seeded at 8000 cells/well in 96 well plate and the day after cell seeding the medium (DMEM and Ham’s for HGFs and AGS, respectively) was replaced by a fresh one containing compounds **1**–**23** at 10 µM for HGFs and at 10 µM and 50 µM for AGS. Cells were incubated up to 72 h in a humidified atmosphere of 5% (*v*/*v*) CO_2_ at 37 °C.

### 3.4. MTT Metabolic Activity Assay

After 48 and 72 h of culture of HGFs and AGS in presence of compounds **1**–**23** and of doxorubicin, an MTT (3-(4,5-dimethylthiazol-2-yl)-2,5-diphenyltetrazolium bromide) test was carried out. MTT test. The MTT test is based on the viable cells capability to reduce MTT into a violet formazan salt. At the established experimental times, the medium was replaced by a fresh one containing 10% of MTT (Sigma Aldrich, Milan, Italy) and probed with cells at 37 °C for 5 h. The plate was then incubated in DMSO for 30 min at 37 °C to allow formazan salts dissolution. The final violet solution was read at 540 nm by means of a microplate reader (Multiskan GO, Thermo Scientific, Waltham, MA, USA). Data obtained without cells were established as background. Viability level was normalized with values derived from cells treated with DMSO.

### 3.5. Cytotoxicity Assay (LDH Assay)

To assess membrane integrity of AGS cells, lactate dehydrogenase (LDH) leakage into the culture medium was measured by means of “CytoTox 96 nonradioactive cytotoxicity assay” (Promega, Madison, WI, USA), following the manufacturer’s instructions, after 48 and 72 of culture in presence of compounds **12** and **22**. In each well, the measured LDH leakage in the supernatant was normalized to the MTT optical density values obtained from MTT test.

### 3.6. Light Microscopy Analysis

AGS cells cultured on Thermanox were fixed in 4% paraformaldehyde for 50 min, washed in PBS and counterstained with haematoxylin-eosin solution. Slides were washed in PBS, dehydrated through the alcohol ascending series and mounted with a permanent xylene-based balsam. The coloured slides were examined by means of Leica DM 4000 light microscope (Leica Cambridge Ltd., Cambridge, UK) equipped with a Leica DFC 320 camera (Leica Cambridge Ltd., Cambridge, UK). Computerized images were acquired with LASX image software (Leica Cambridge Ltd., Cambridge, UK).

### 3.7. Hydrogen Peroxide (H_2_O_2_) Release

At the established experimental times cell supernatants were collected. The quantitative measurement of H_2_O_2_ released in supernatant was carried out through the Hydrogen Peroxide colorimetric detection kit (Enzo Life Sciences, Inc., Farmingdale, NY, USA). For each sample 50 µL of supernatant/well were loaded, in duplicate, in a Half-Area Microtiter Plate, then 100 µL of Color Reagent were added in each well. The multiwell plate was shacked for 10 s and then incubated for 30 min at room temperature. The optical density (OD) was measured at 550 nm by means of a spectrophotometer (Multiskan GO; Thermo Fisher Scientific, Inc., Waltham, MA USA). The results were calculated by subtracting the average blank OD from the average OD for each sample.

### 3.8. Bax Immunostaining by Flow Cytometry

At the established exposure times, the incubation medium was discarded and cells were incubated with the Accutase^®^ solution (Sigma-Aldrich, St. Louis, MO, USA) for 5 min at 37 °C and 5% CO_2_ for cell detachment. After that, cells were collected, centrifuged at 1200 rpm for 10 min and the obtained pellets were afterwards washed with PBS without calcium and magnesium. Next, supernatants were discarded and cell pellets were fixed for 15 min at room temperature with the PerFix-nc Buffer 1 from the commercial PerFix-nc Kit (no-centrifuge assay) (Beckman Coulter, Indianapolis, IN, USA). Fixed sample were gently vortexed after having added 100 µL/sample of the permeabilizing reagent PerFix-nc Buffer 2 (Beckman Coulter) containing 5% of FBS, and incubated for additional 20 min at room temperature. After the permeabilization, the mouse anti-Bax monoclonal antibody (sc-7480, Santa Cruz Biotechnology, Santa Cruz, CA, USA) was added in a dilution of 1:50 and samples were incubated for 1 h at 4 °C. Next, cells were centrifuged for 10 min at 4 °C, the supernatant was discarded and the secondary phycoerythrin (PE) horse anti-mouse IgG (H+L) antibody (Vector Laboratories, Inc., Burlingame, CA, USA) was added (1:50) and incubated at 4 °C in the dark for 45 min. Secondary antibody overage was removed by centrifugation and cells were suspended in 300 µL of PerFix-nc Buffer 3 1X (Beckman Coulter). Finally, 10,000 cells for each sample were run in a CytoFlex Flow cytometer (Beckman Coulter) equipped with a 488 nm laser using the FL-2 (PE) channel in a linear mode. Relative fluorescence emissions of gated cells by means of their forward and side scatter properties (FSC/SSC) were analyzed with the CytExpert Software (Beckman Coulter) and they were expressed as the percentage of positive cells for Bax-PE conjugated. Individual values obtained from three independent experiments (*n* = 3) were summarized as means and standard deviations.

### 3.9. Statistical Analysis

Statistical analysis was performed using the GraphPad 7 software (GraphPad Software, San Diego, CA, USA) by means of *t*-test and Ordinary One-Way ANOVA followed by post-hoc Tukey’s multiple comparisons tests. Values of *p* < 0.05 were considered statistically significant.

## 4. Conclusions

**Figure 10:**
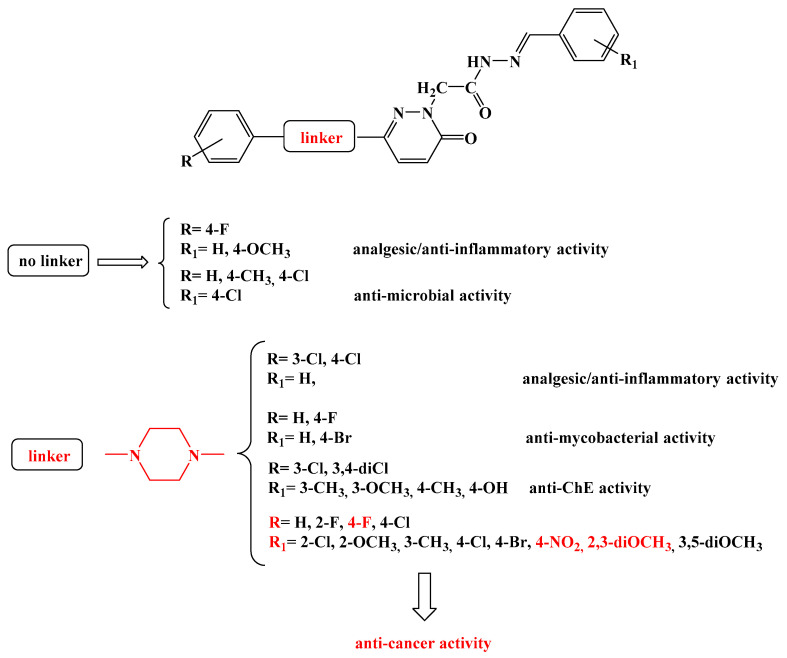
Update of the SARs within the pyridazinone scaffold for the design of biologically active compounds. The structural information gained in this work have been highlighted as red text.

We previously investigated the anti-proliferative effects of a large number of pyridazinones against HCT116 cells [36]. To further expand the knowledge of the SARs within this scaffold, we introduced a piperazinyl linker between the pyridazinone core nucleus and the additional phenyl ring aiming at potentiating the biological activity and reducing the cytotoxicity against a non-cancerous cell line. The results of the MTT tests have been compared to those of doxorubicin as a reference drug. We selected the two best-in-class compounds **12** and **22** and better explored their mechanism of action in terms of pro-apoptotic ability. These compounds induced a change in the cell morphology, a release of oxidant hydrogen peroxide and the expression of Bax, thus confirming their anti-proliferative role against AGS cells. On the basis of these results, we were also able to update the SAR studies within this scaffold as reported in Figure 10.

## Figures and Tables

**Table 1:**
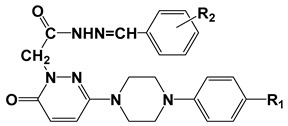
Structures, yields and chemical-physical data of title compounds.

Compd	R_1_	R_2_	Log P	Log S	tPSA	M.W.	Molecular Formula
**1**	H	H	3.00	−6.19	80.61	416.48	C_23_H_24_N_6_O_2_
**2**	H	3002DCH_3_	3.49	−6.54	80.61	430.50	C_24_H_26_N_6_O_2_
**3**	H	4-CH_3_	3.49	−6.55	80.61	430.50	C_24_H_26_N_6_O_2_
**4**	H	4-OCH_3_	2.87	−6.25	89.84	446.50	C_24_H_26_N_6_O_3_
**5**	H	2,3-OCH_3_	2.75	−6.07	99.07	476.53	C_25_H_28_N_6_O_4_
**6**	H	3,5-OCH_3_	2.75	−6.29	99.07	476.53	C_25_H_28_N_6_O_4_
**7**	H	2,4,6-OCH_3_	2.62	−6.34	108.30	506.55	C_26_H_30_N_6_O_5_
**8**	H	3,4,5-OCH_3_	2.62	−6.10	108.30	506.55	C_26_H_30_N_6_O_5_
**9**	H	4-NO_2_	2.48	−6.58	132.42	461.47	C_23_H_23_N_7_O_4_
**10**	Cl	2-OCH_3_	3.43	−6.95	89.84	480.95	C_24_H_25_ClN_6_O_3_
**11**	Cl	3-OCH_3_	3.43	−6.95	89.84	480.95	C_24_H_25_ClN_6_O_3_
**12**	Cl	2,3-OCH_3_	3.31	−6.77	99.07	510.97	C_25_H_27_ClN_6_O_4_
**13**	Cl	2,4,6-OCH_3_	3.18	−7.05	108.30	541.00	C_26_H_29_ClN_6_O_5_
**14**	Cl	3,4,5-OCH_3_	3.18	−6.80	108.30	541.00	C_26_H_29_ClN_6_O_5_
**15**	Cl	4-NO_2_	3.10	−7.29	132.42	495.92	C_23_H_22_ClN_7_O_4_
**16**	Cl	4-N(CH_3_)_2_	3.84	−7.29	83.85	493.99	C_25_H_28_ClN_7_O_2_
**17**	F	H	3.16	−6.46	80.61	434.47	C_23_H_23_FN_6_O_2_
**18**	F	3-CH_3_	3.65	−6.82	80.61	448.49	C_24_H_25_FN_6_O_2_
**19**	F	3-OCH_3_	3.03	−6.51	89.84	464.49	C_24_H_25_FN_6_O_3_
**20**	F	4-OCH_3_	3.03	−6.52	89.84	464.49	C_24_H_25_FN_6_O_3_
**21**	F	2,4,6-OCH_3_	2.78	−6.62	108.30	524.54	C_26_H_29_FN_6_O_5_
**22**	F	4-NO_2_	2.62	−6.86	132.42	479.46	C_23_H_22_FN_7_O_4_
**23**	F	4-N(CH_3_)_2_	3.44	−6.86	83.85	477.53	C_25_H_28_FN_7_O_2_

Log P: partition coefficient; Log S: intrinsic solubility; tPSA: total polar surface area.

**Table 2:** In silico physical-chemical properties the most potent compounds **12** and **22**.

Compound	12	22
**H-bond acceptors (HBA)**	5	6
**H-bond donators (HBD)**	1	1
**Consensus Log P**	2.59	2.41
**Lipinski violations**	0	0
**GI absorption**	high	high
**P-gp substrate**	yes	yes
**PAINS alerts**	0	0

**Table 3:** Protein target prediction for compound **12**.

Target	Common Name	Uniprot ID	ChEMBL ID	Target Class	Probability
Neuropeptide Y receptor type 5	NPY5R	Q15761	CHEMBL4561	Family A G protein-coupled receptor	0.12
P2X purinoceptor 7	P2RX7	Q99572	CHEMBL4805	Ligand-gated ion channel	0.12
Adenosine A2a receptor	ADORA2A	P29274	CHEMBL251	Family A G protein-coupled receptor	0.12
Inhibitor of nuclear factor kappa B kinase beta subunit	IKBKB	O14920	CHEMBL1991	Kinase	0.12
Toll-like receptor 4	TLR4	O00206	CHEMBL5255	Toll-like and Il-1 receptors	0.12

**Table 4:** Protein target prediction for compound **22**.

Target	Common Name	Uniprot ID	ChEMBL ID	Target Class	Probability
Probable protein-cysteine *N*-palmitoyltransferase porcupine	PORCN	Q9H237	CHEMBL1255163	Enzyme	0.12
P2X purinoceptor 7	P2RX7	Q99572	CHEMBL4805	Ligand-gated ion channel	0.12
Dual specificity mitogen-activated protein kinase kinase 2	MAP2K2	P36507	CHEMBL2964	Kinase	0.12
Dual specificity mitogen-activated protein kinase kinase 1	MAP2K1	Q02750	CHEMBL3587	Kinase	0.12
Inhibitor of nuclear factor kappa B kinase beta subunit	IKBKB	O14920	CHEMBL1991	Kinase	0.12

## Data Availability

Data are contained within the article.

## Supplementary Materials

The following are available online at https://www.mdpi.com/1424-8247/14/3/183/s1, ^1^H-NMR, ^13^C-NMR, and mass spectra for each compound.
