# Supplementary material for: Synthesis, Cytotoxicity and Anti-Proliferative Activity against AGS Cells of New 3(2H)-Pyridazinone Derivatives Endowed with a Piperazinyl Linker
Source: Pharmaceuticals (Basel). 2021 Feb 25;14(3):183. doi: 10.3390/ph14030183 (PMC7996573; doi:10.3390/ph14030183)
Supplement: Supplementary file 1 [file pharmaceuticals-14-00183-s001.zip › pharmaceuticals-1106113-supplementary.pdf]

Data 1. ^{1}H-NMR spectral analysis of compound **1**

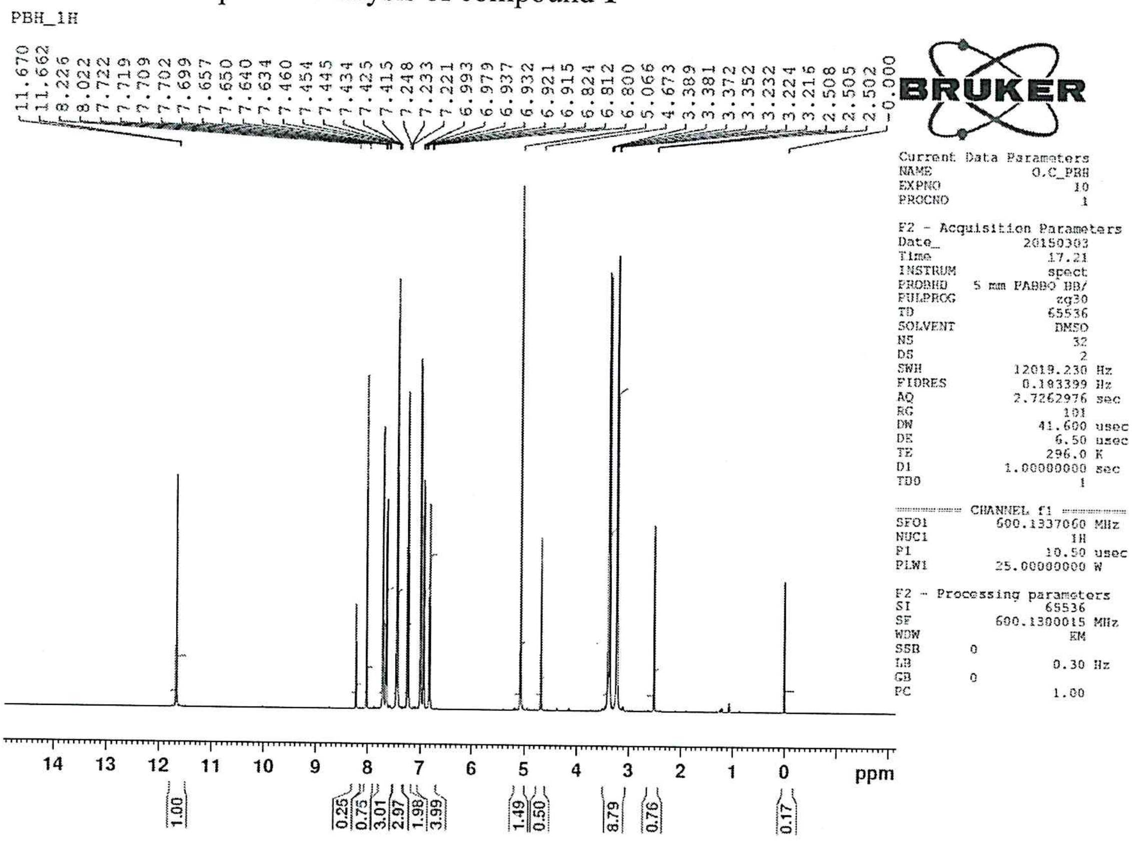

Data 2. ^{13}C-NMR spectral analysys of compound **1**

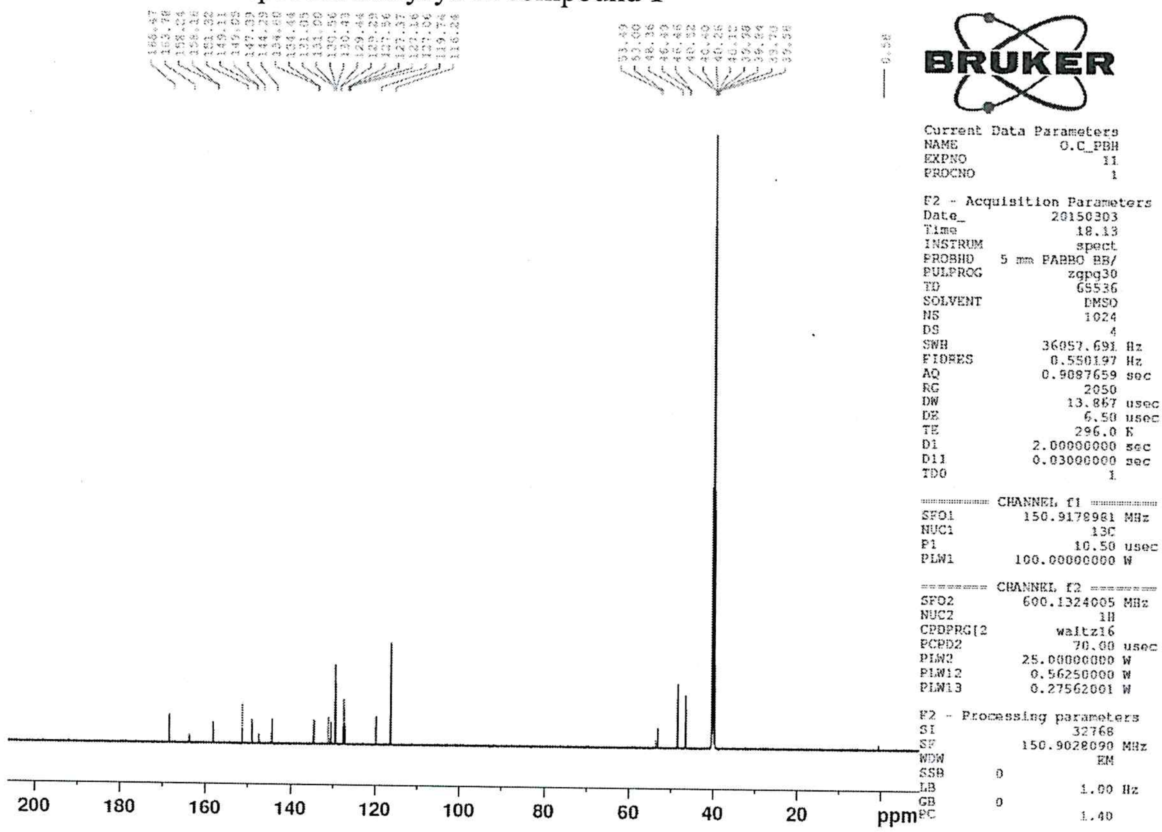

Data 3. Mass spectral analysis of compound **1**

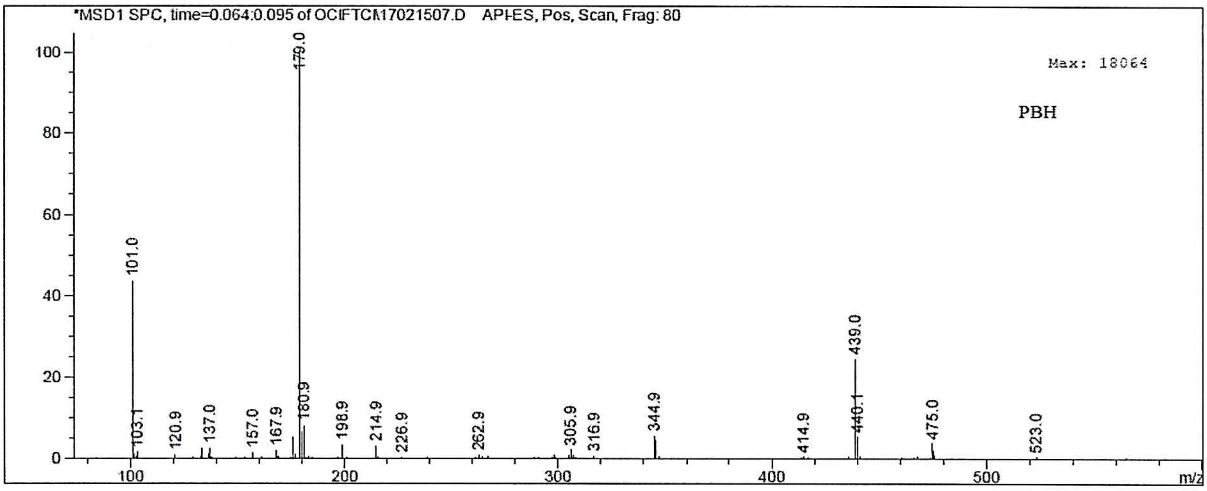

Data 4. ¹H-NMR spectral analysis of compound 2

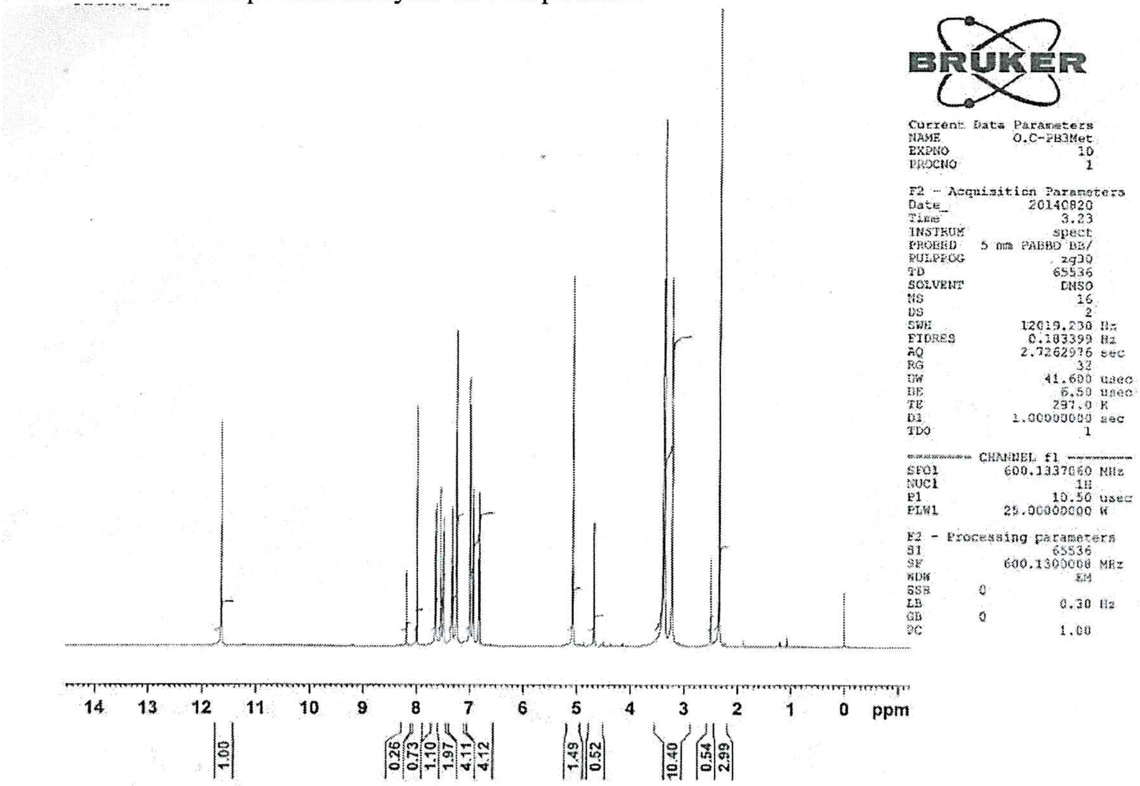

Data 5. ^{13}C-NMR spectral analysis of compound **2**

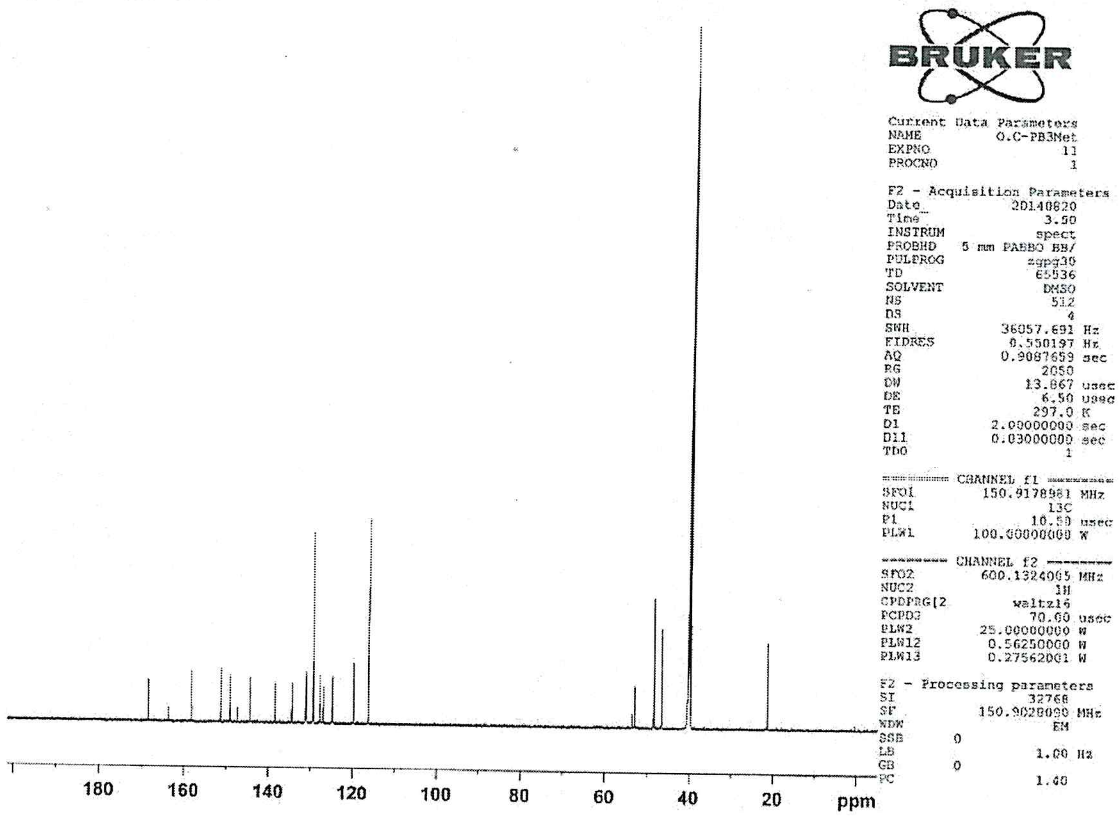

Data 6. Mass spectral analysis of compound **2**

Qualitative Analysis Report

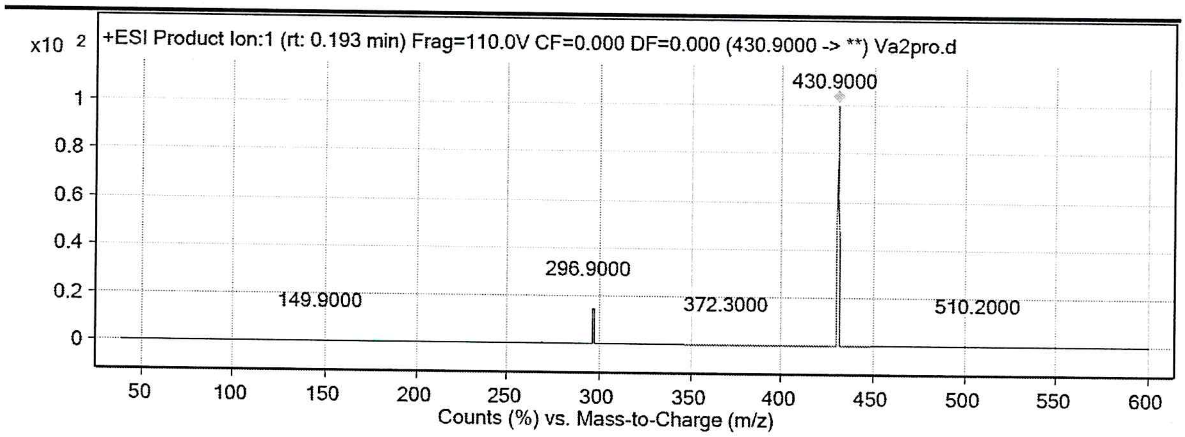

Peak List

m/z	z	Abund
92.5	2	55.68
118		59.1
149.9		64.42
241.4	2	66.78
242.2		79.38
252.5	2	56.42
268.8		1107.38
296.9		25721.48
429.7		423.1
430.9		172665.95

Data 7. ^{1}H-NMR spectral analysis of compound **3**

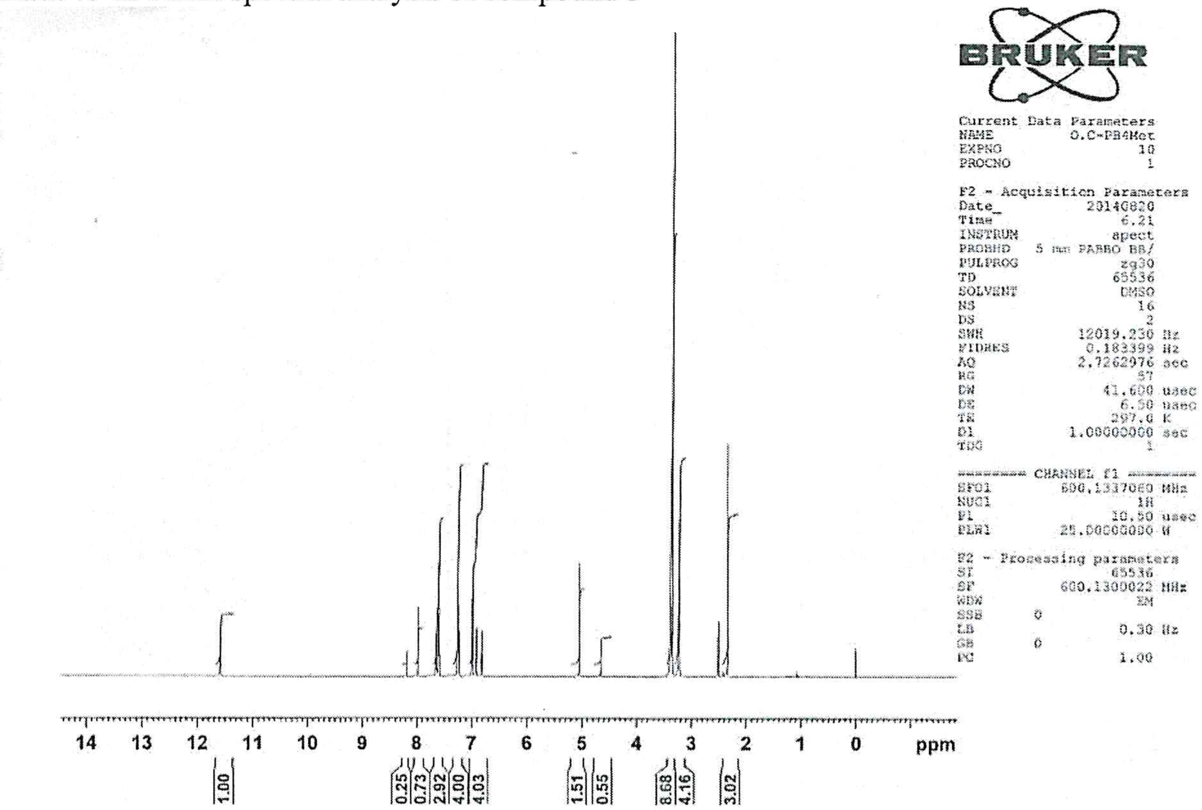

Data 8. ^{13}C-NMR spectral analysis of compound **3**

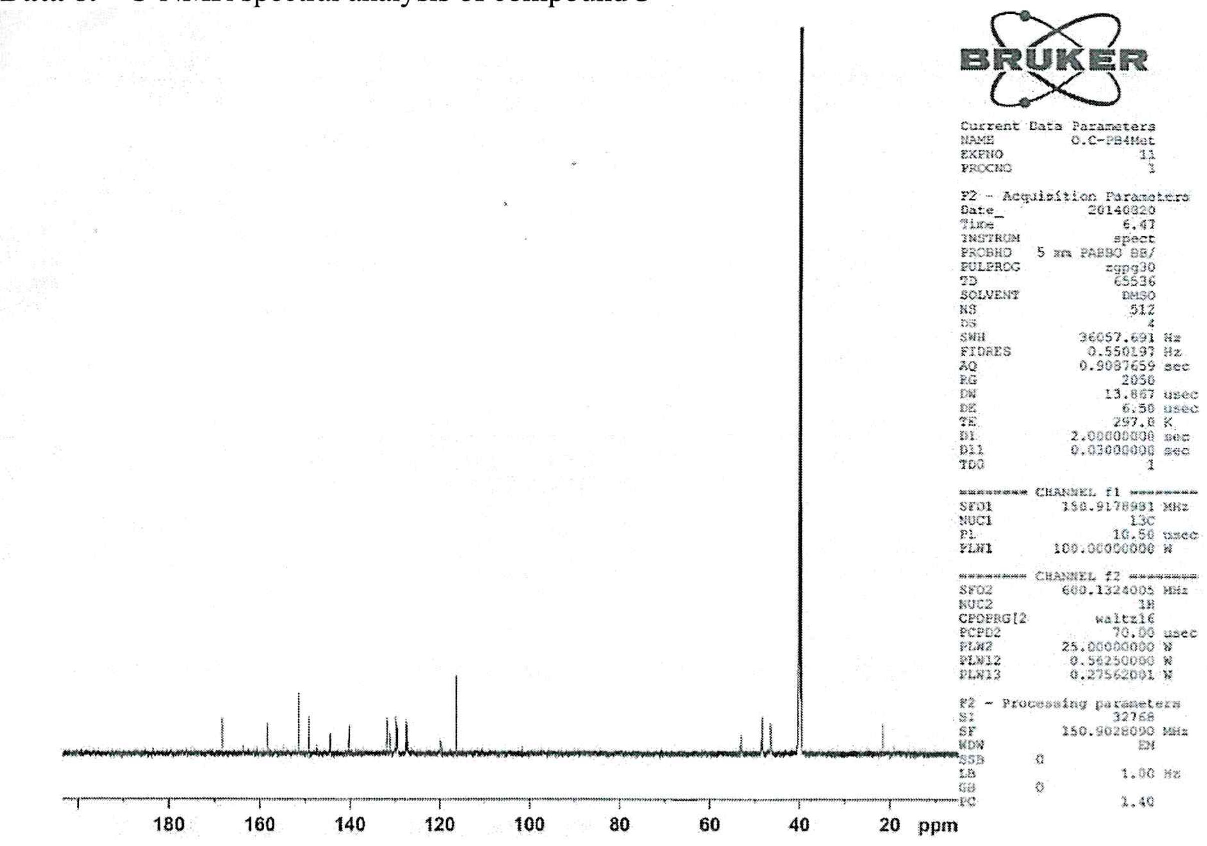

Data 9. Mass spectral analysis of compound **3**

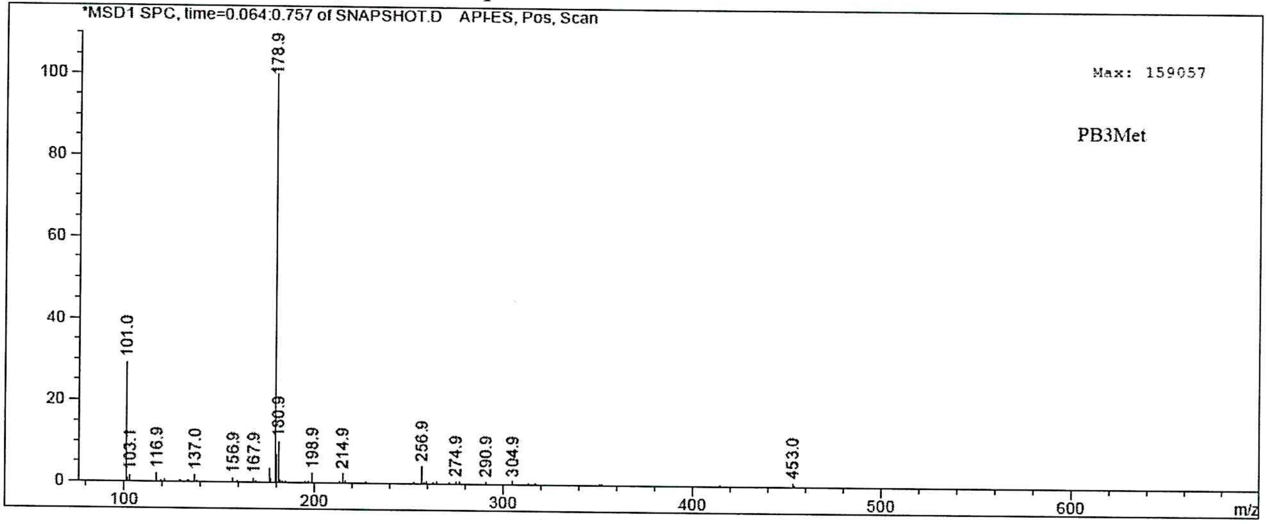

Data 10. ^{1}H-NMR spectral analysis of compound **4**

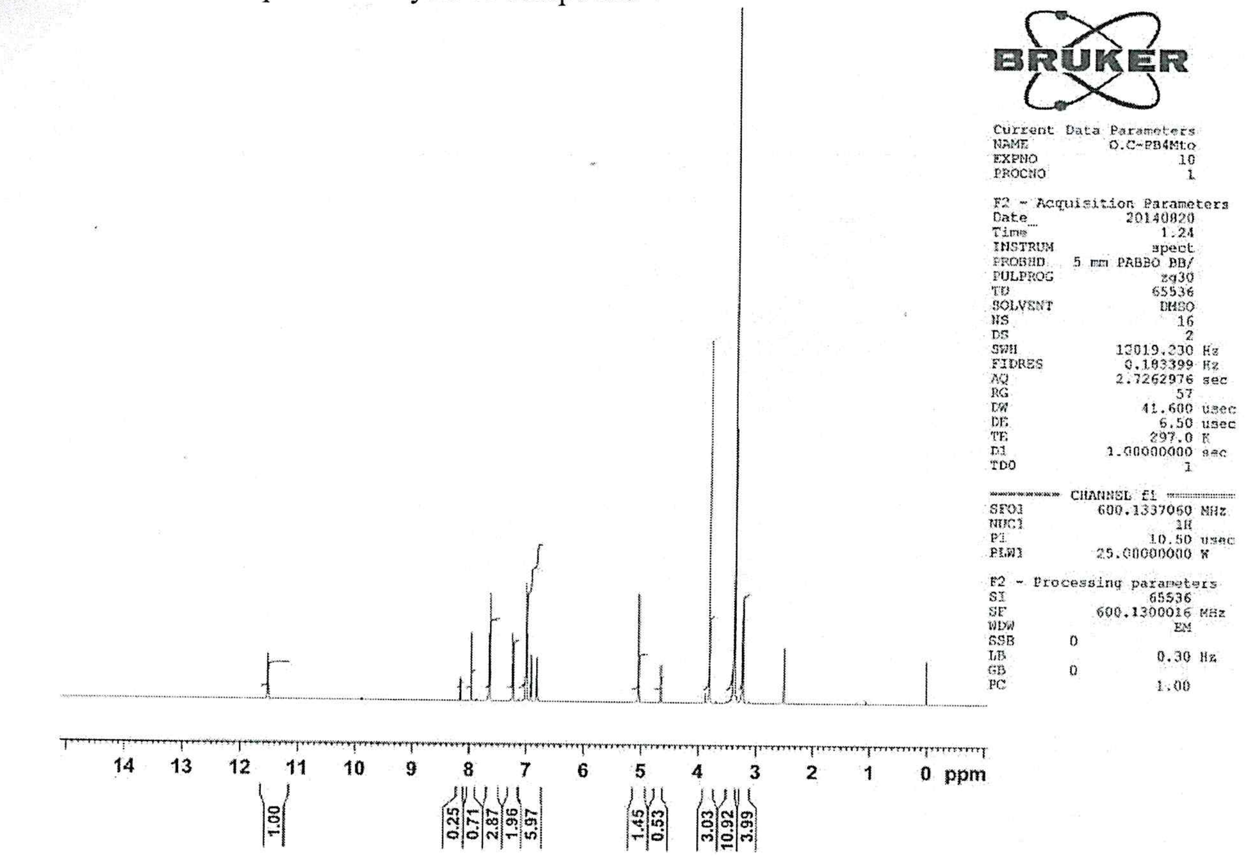

Data 11. ^{13}C-NMR spectral analysis of compound **4**

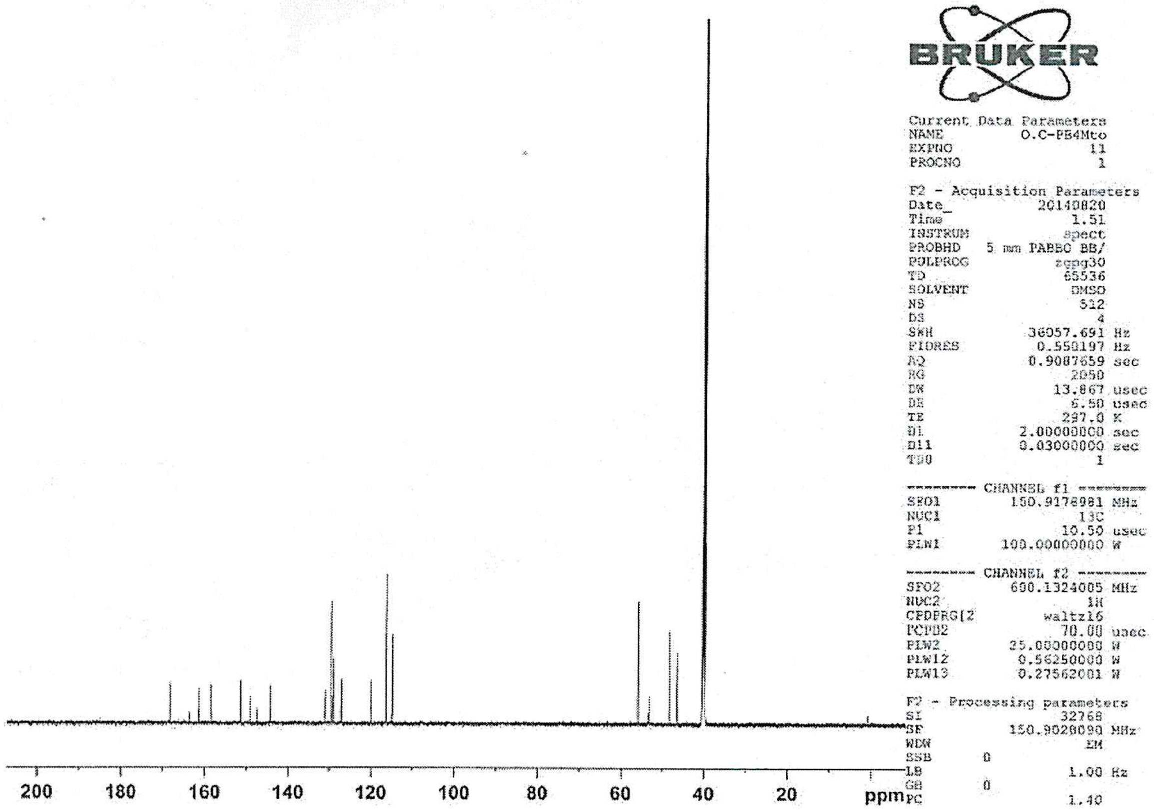

Data 13. Mass spectral analysis of compound **4**

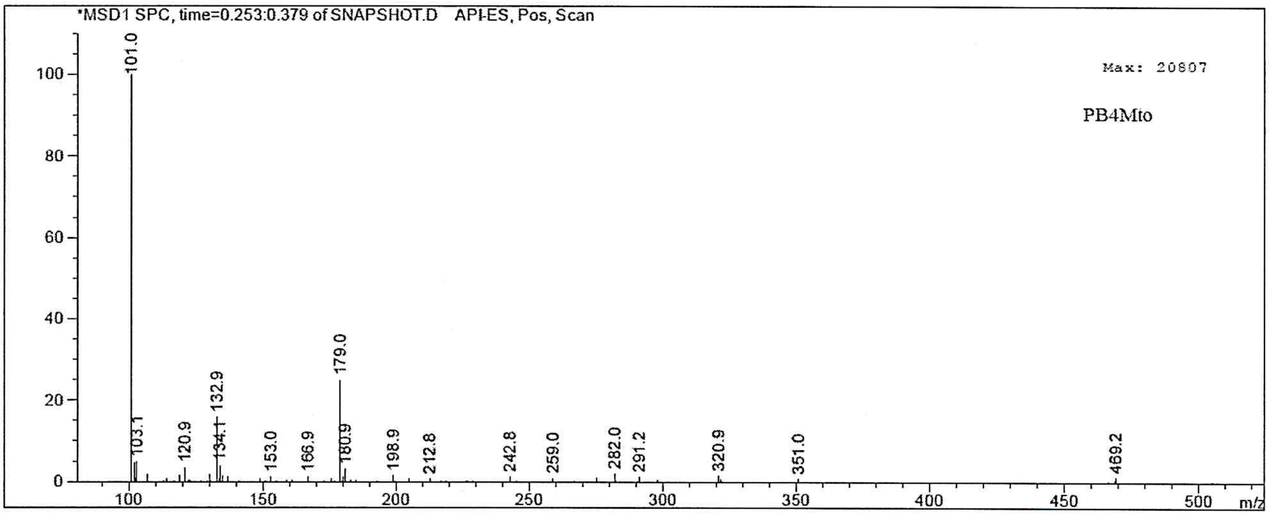

Data 14. ^{1}H-NMR spectral analysis of compound **5**

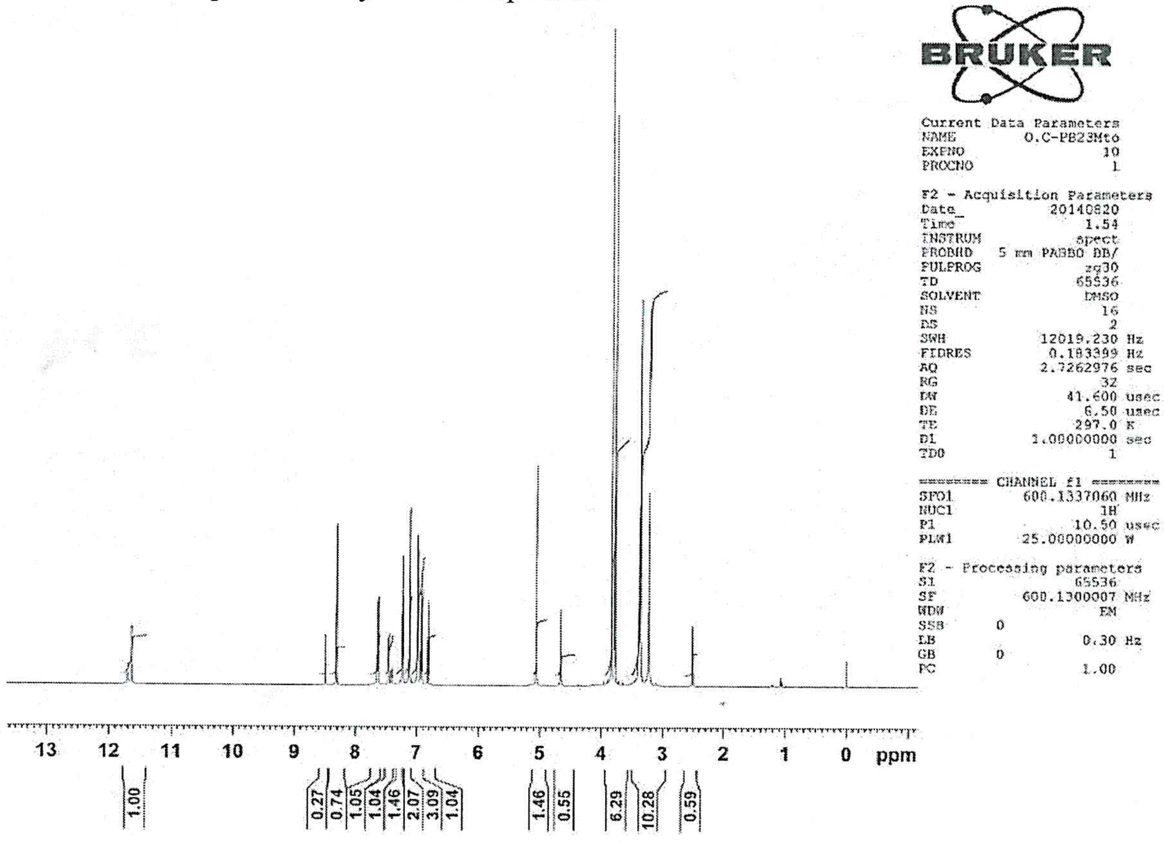

Data 15. ^{13}C-NMR spectral analysis of compound **5**

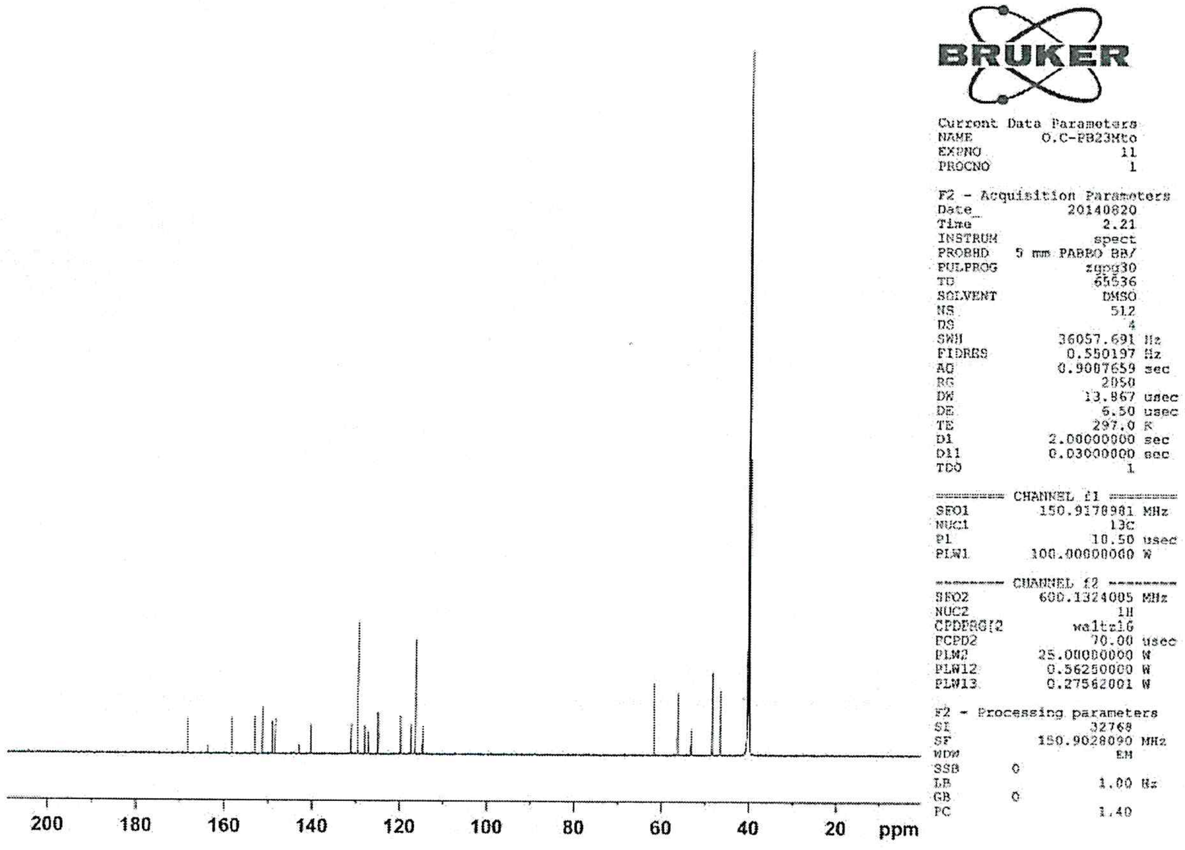

Data 16. Mass spectral analysis of compound **5**

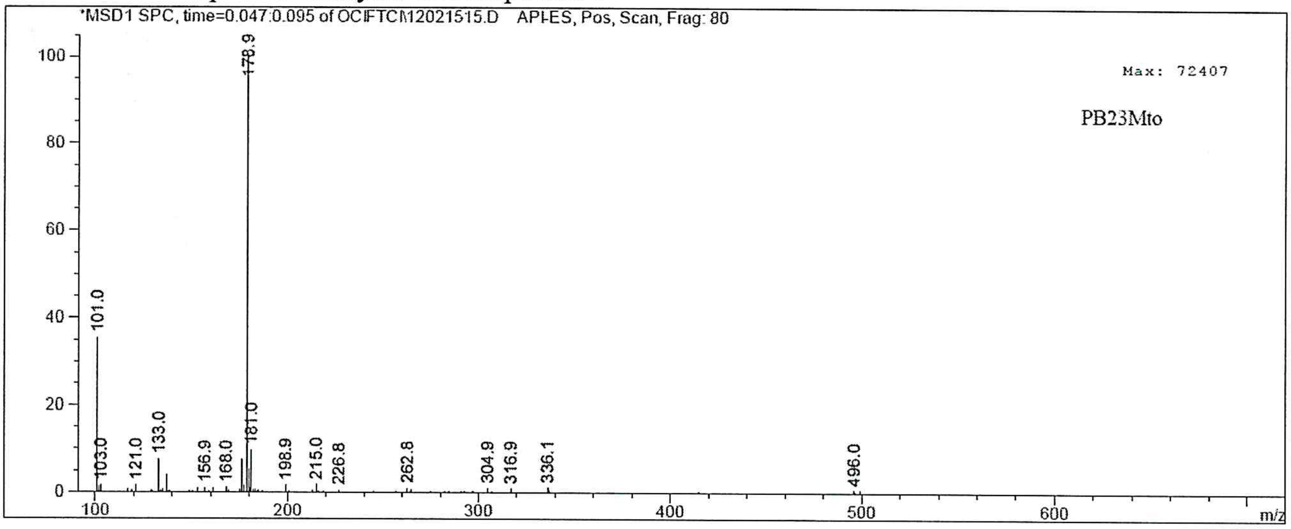

Data 17. ^{1}H-NMR spectral analysis of compound **6**

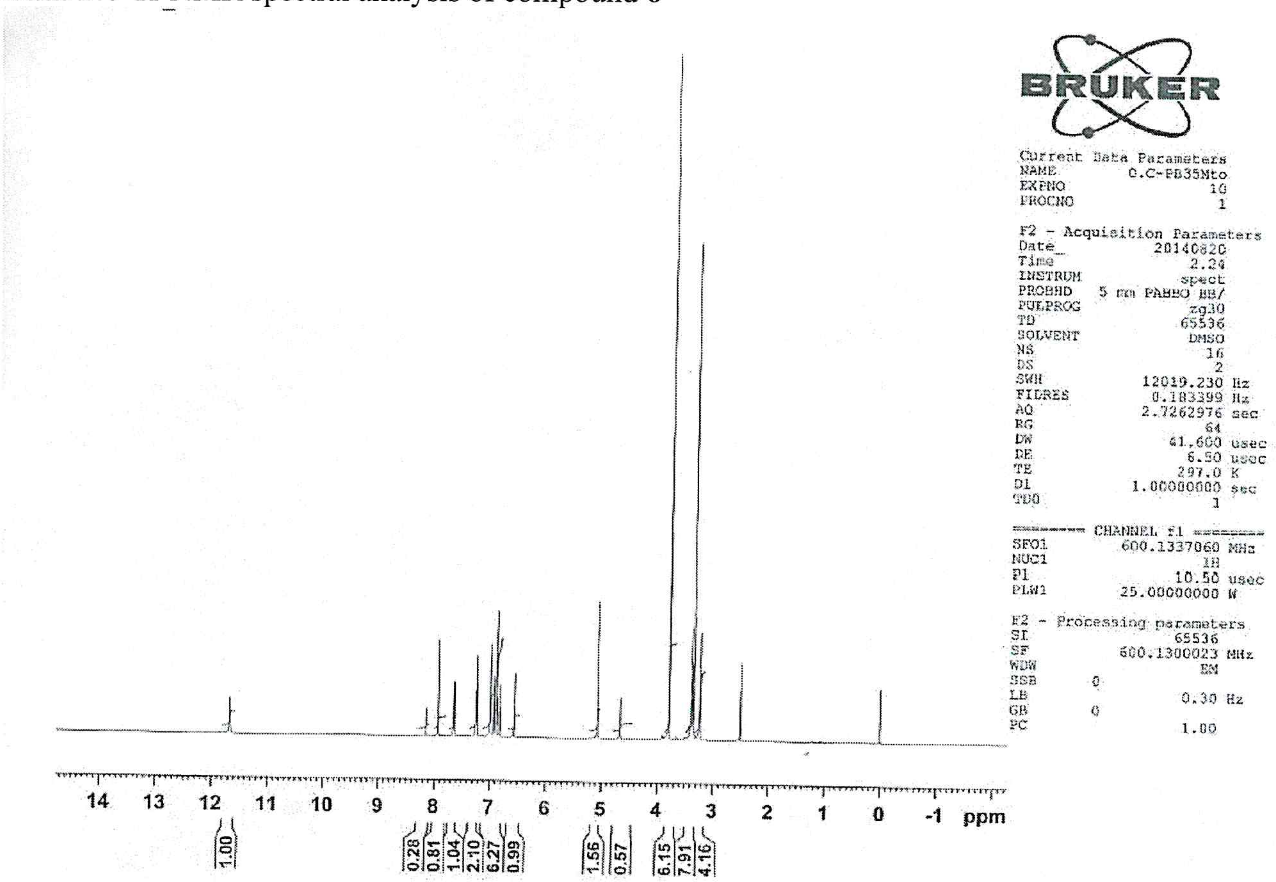

Data 18. ^{13}C-NMR spectral analysis of compound **6**

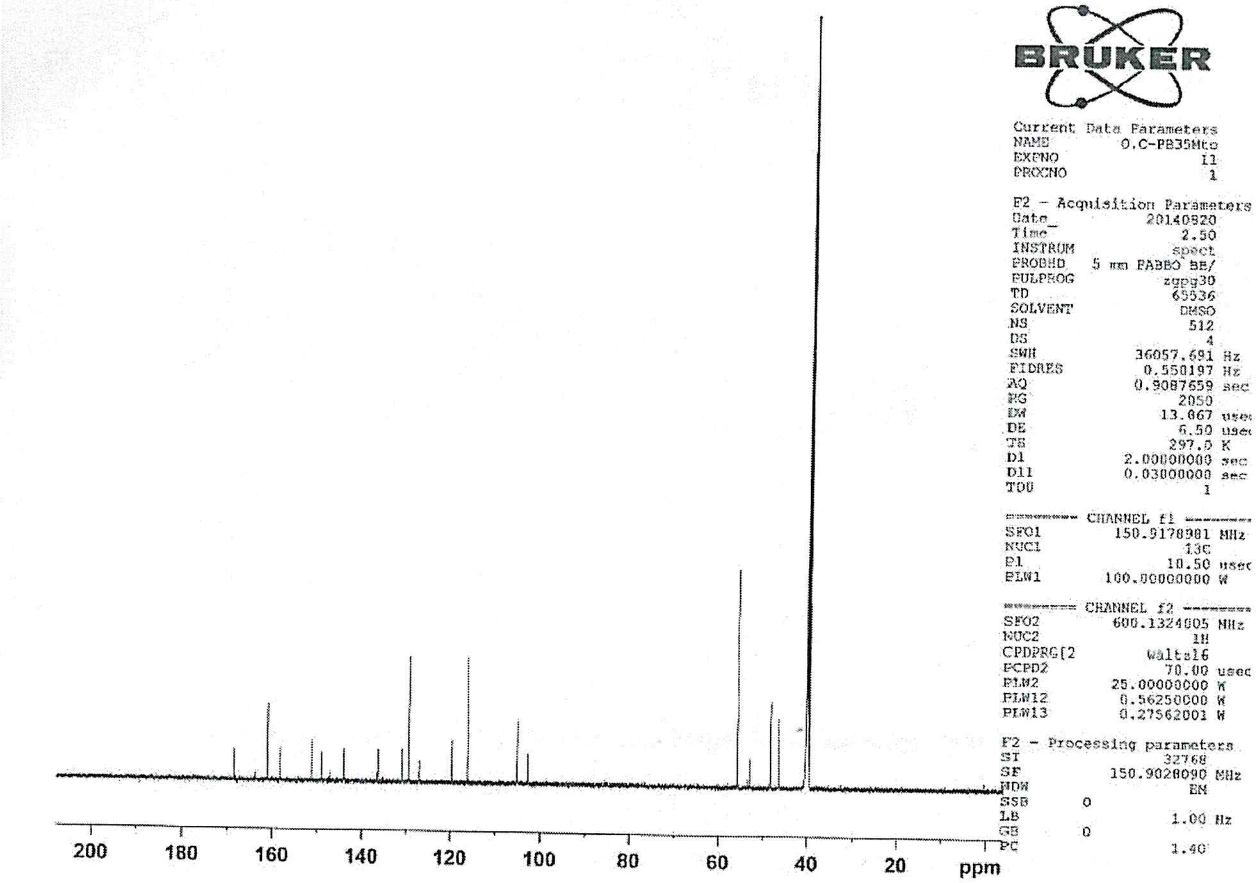

Data 19. Mass spectral analysis of compound **6**

Qualitative Analysis Report

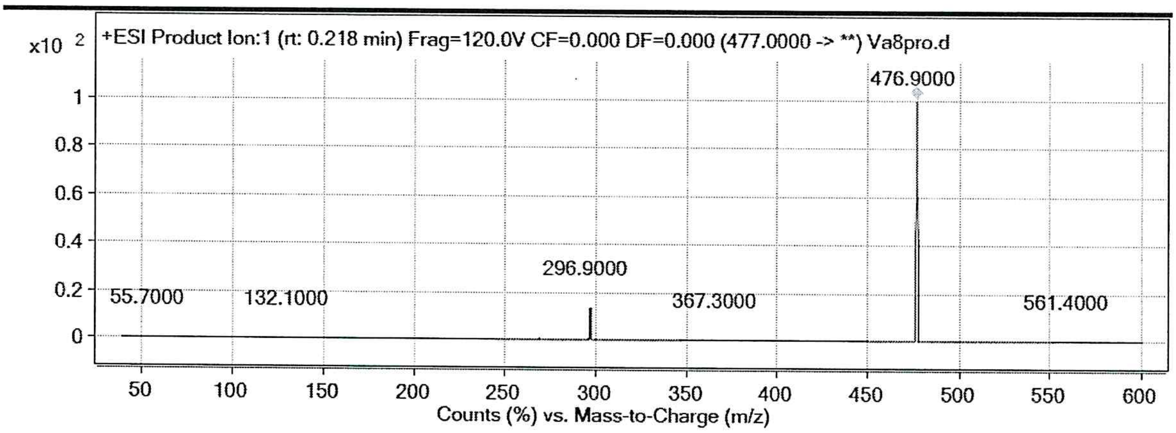

Peak List

m/z	z	Abund
90.5	2	56.5
119.7	2	55.8
132.1		82.58
149.5	2	73.86
241.6	2	66.2
268.9		1360.48
296.9		23633.5
458.8		59.6
475.6		278.38
476.9		174022.17

Data 20. ^{1}H-NMR spectral analysis of compound **7**

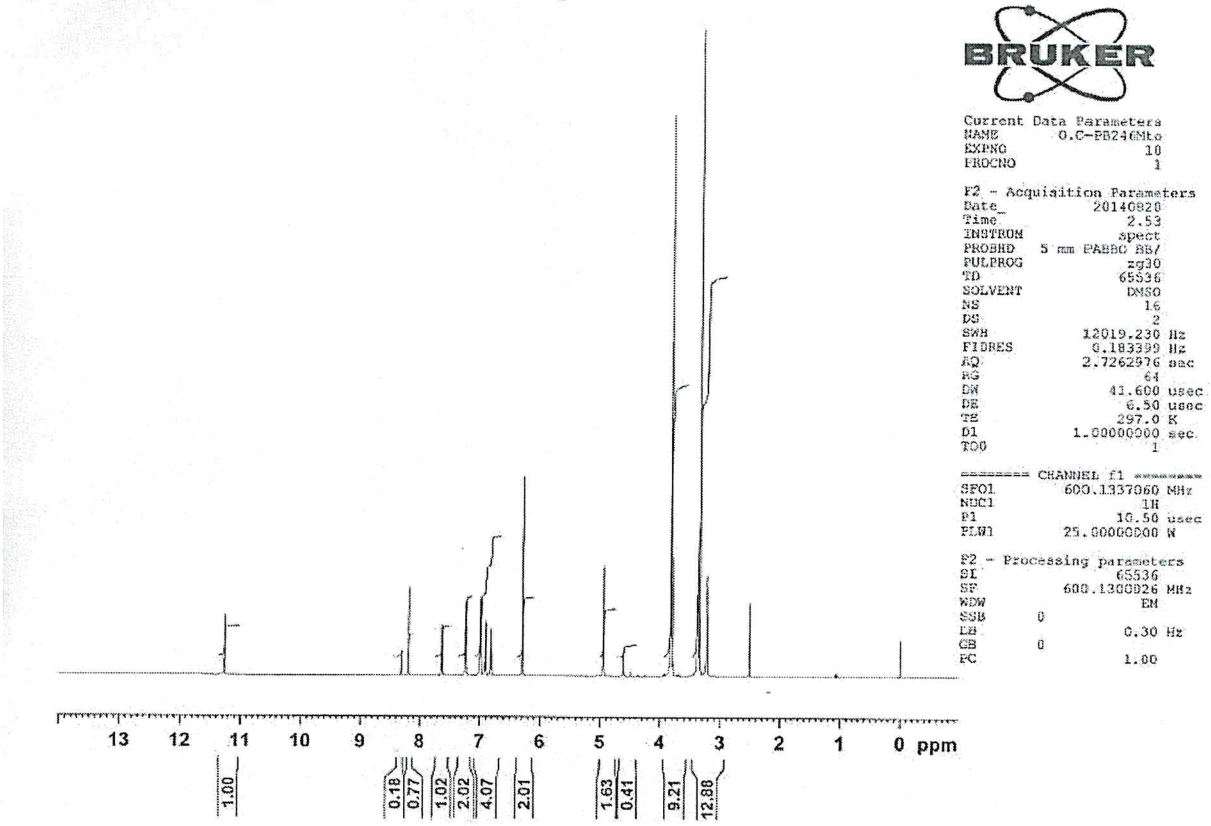

Data 21. ^{13}C-NMR spectral analysis of compound **7**

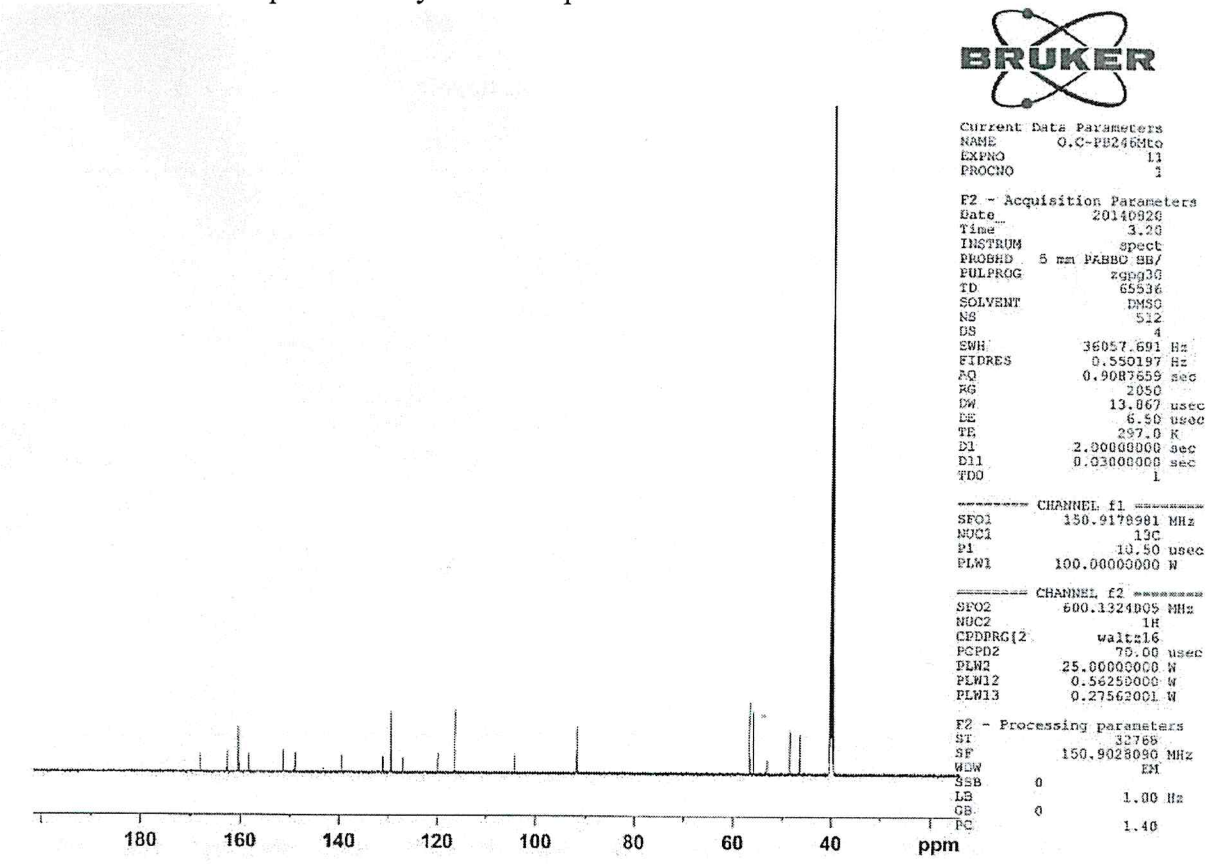

Data 22. Mass spectral analysis of compound **7**

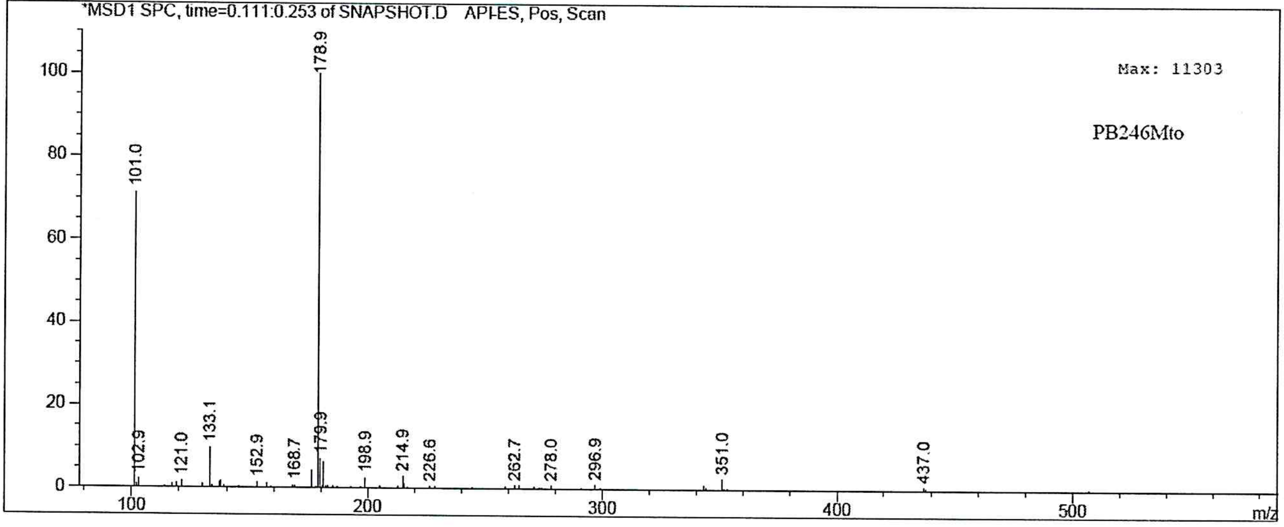

Data 23. ^{1}H-NMR spectral analysis of compound **8**

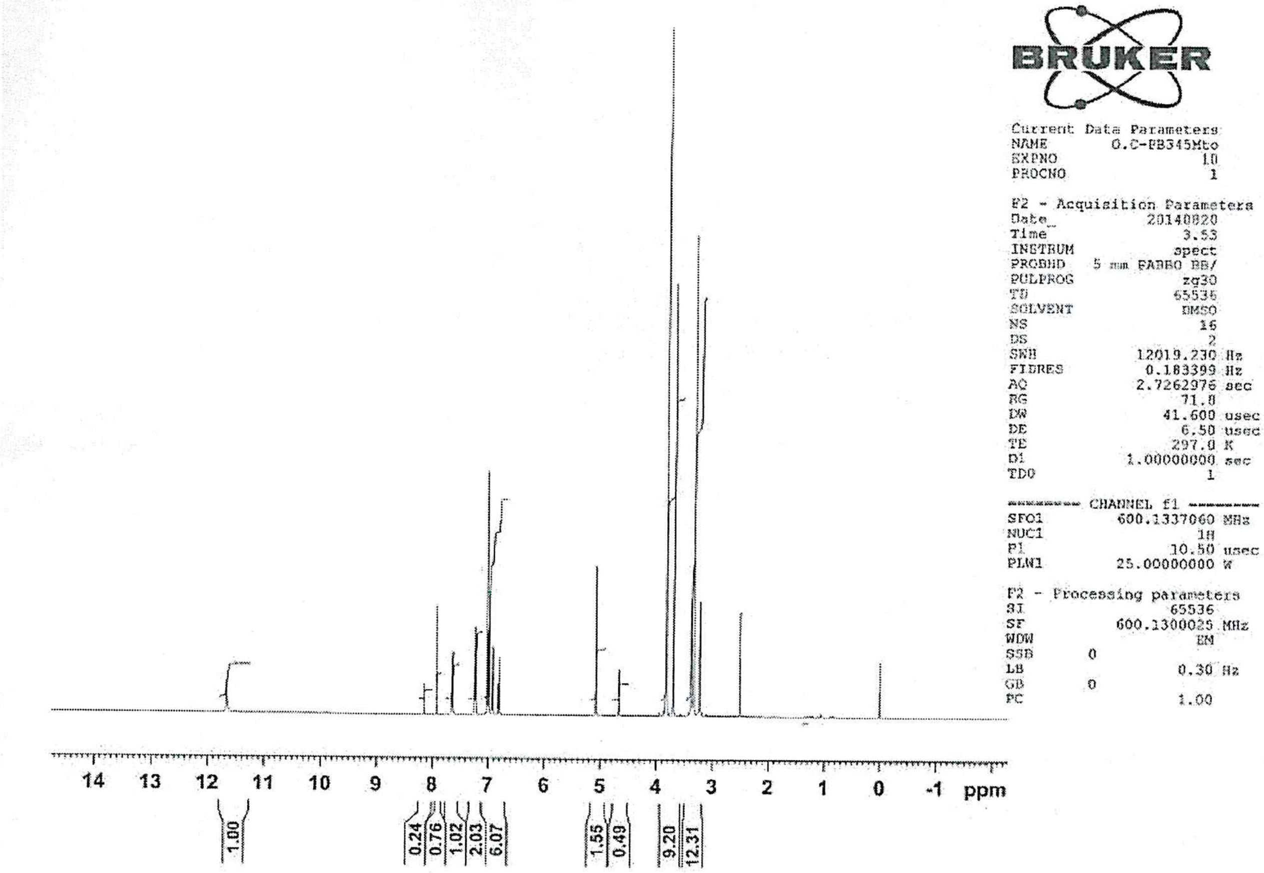

Data 24. ^{13}C-NMR spectral analysis of compound **8**

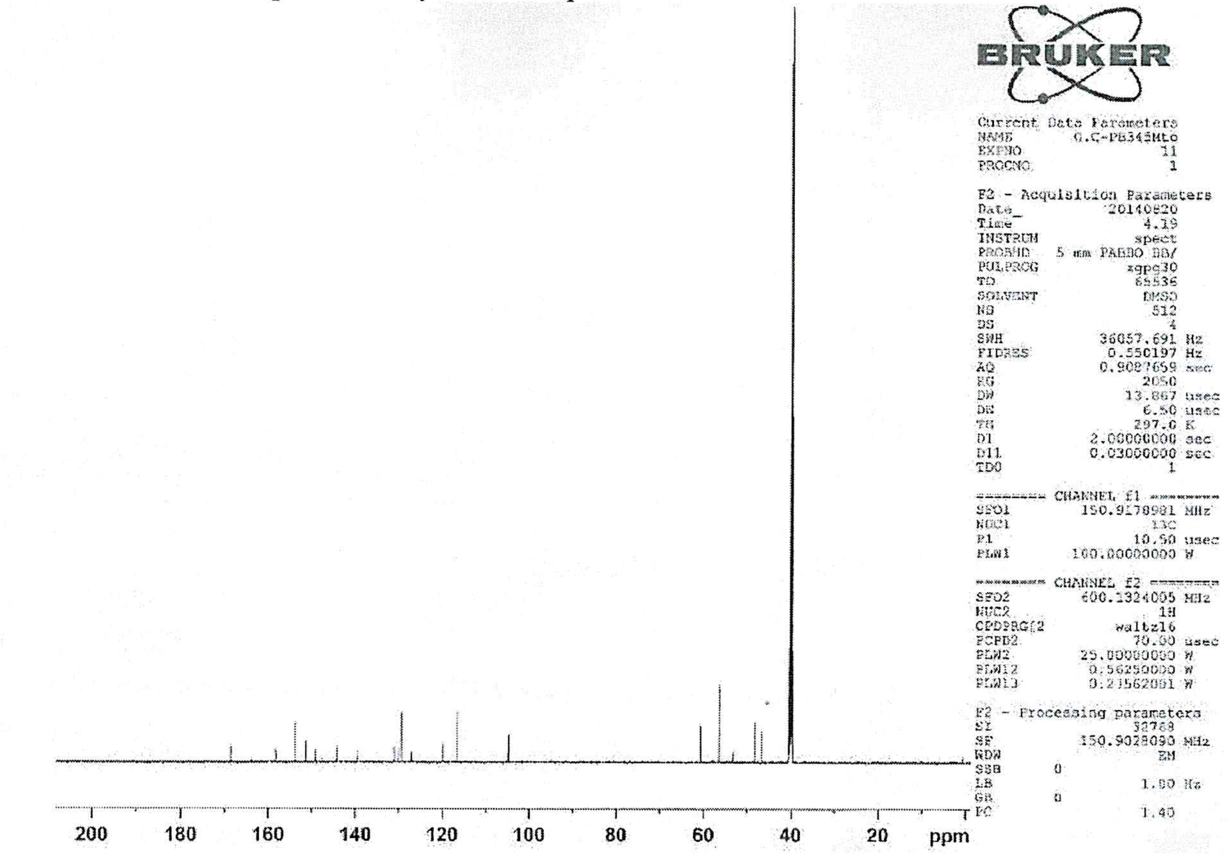

Data 25. Mass spectral analysis of compound **8**

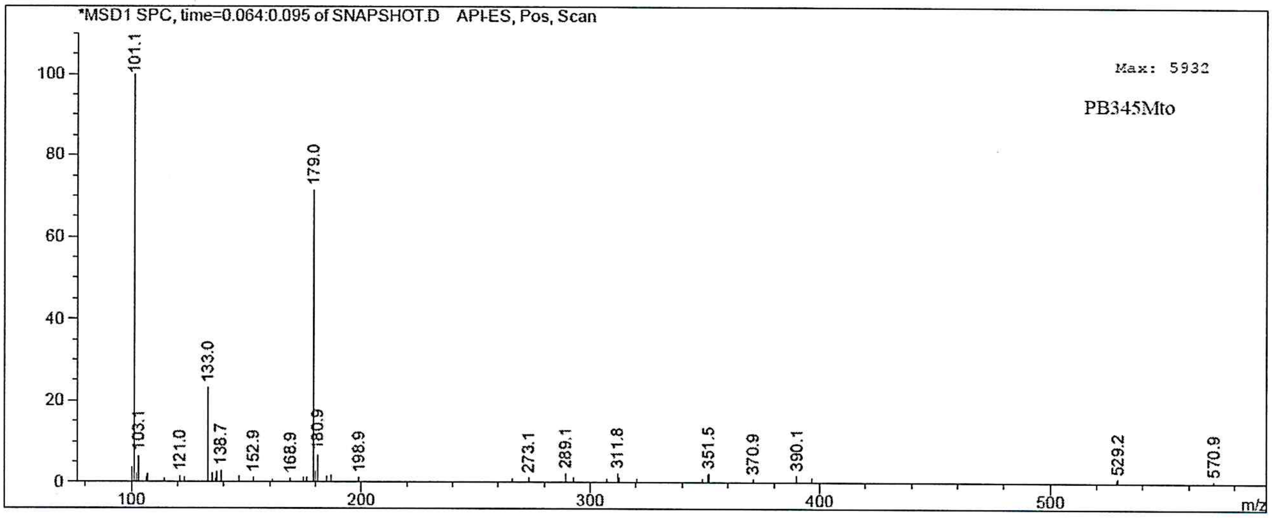

Data 26. ^{1}H-NMR spectral analysis of compound **9**

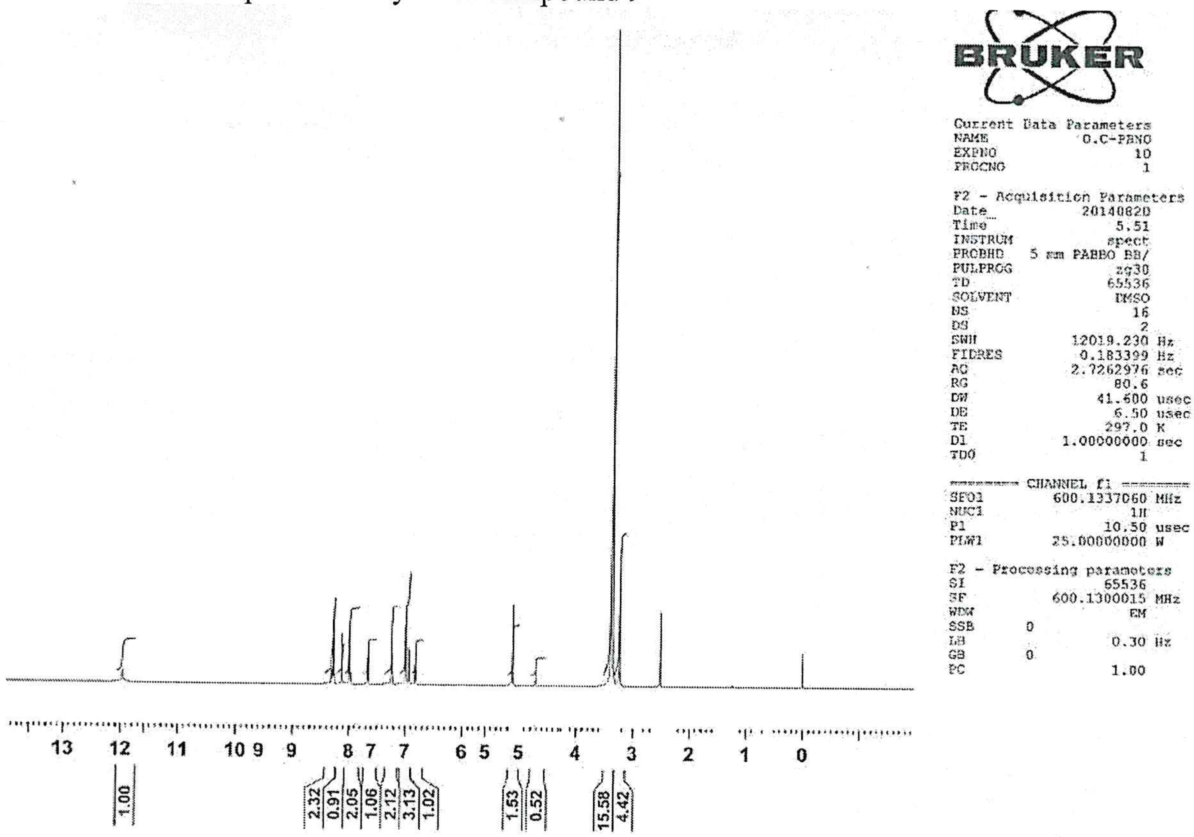

Data 27. ^{13}C-NMR spectral analysis of compound **9**

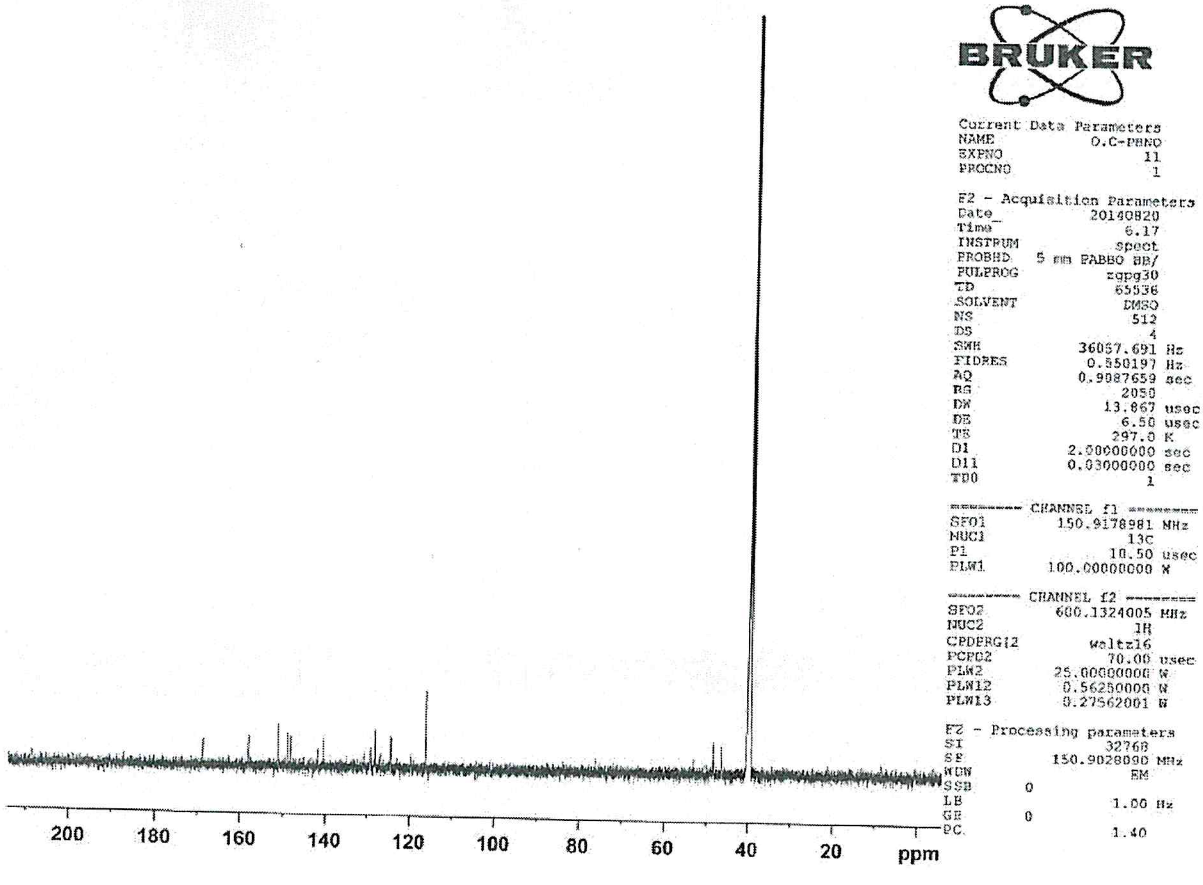

Data 28. Mass spectral analysis of compound **9**

Qualitative Analysis Report

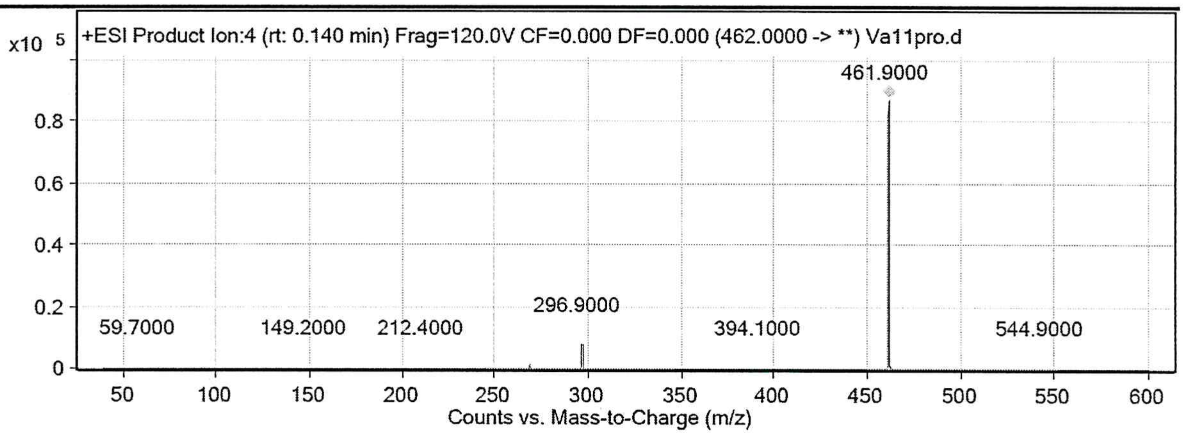

Peak List

m/z	z	Abund
43.7	2	54.98
59.7	2	58.34
109.2		54.48
118.6	2	54.78
149.2		59.66
212.4	2	54.12
268.9		884.76
296.9		7646.08
460.1		63.06
461.9		87040.38

Data 29. ^{1}H-NMR spectral analysis of compound **10**

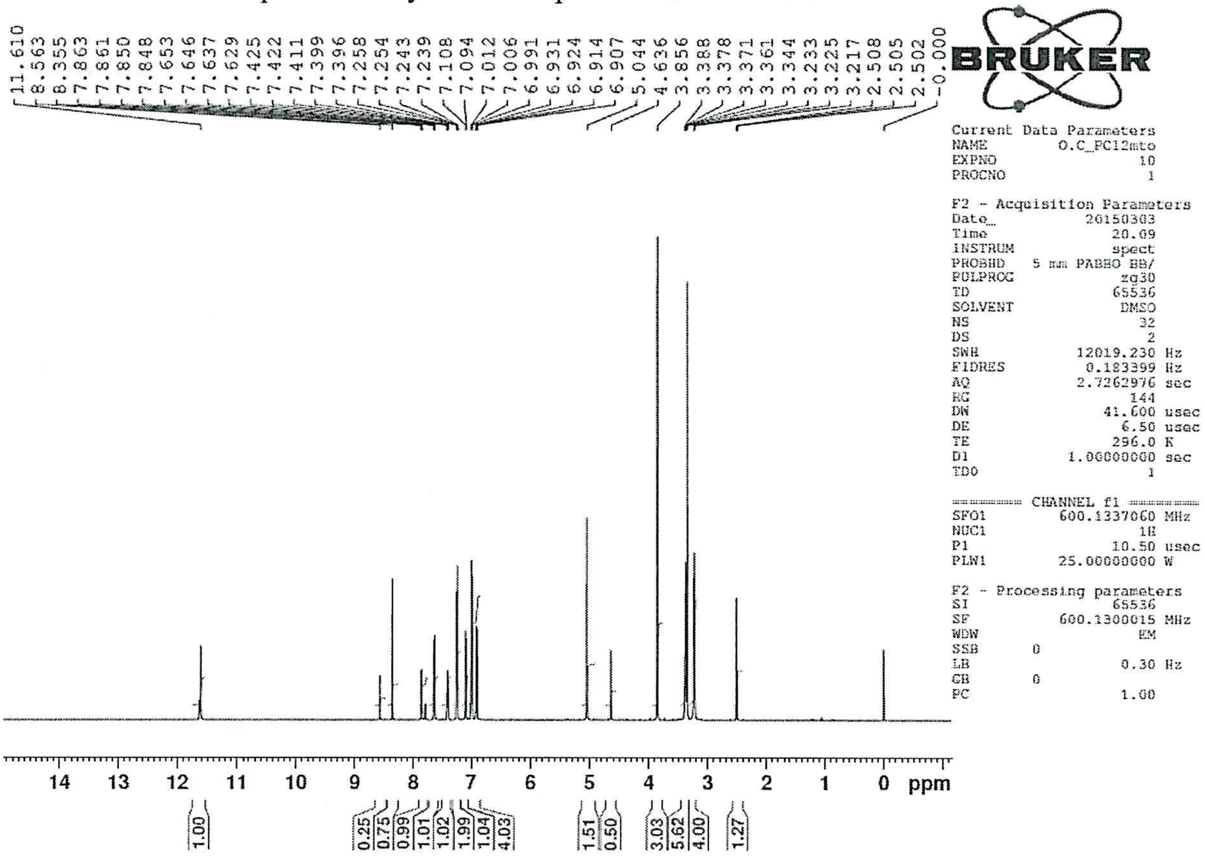

Data 30. ^{13}C-NMR spectral analysis of compound **10**

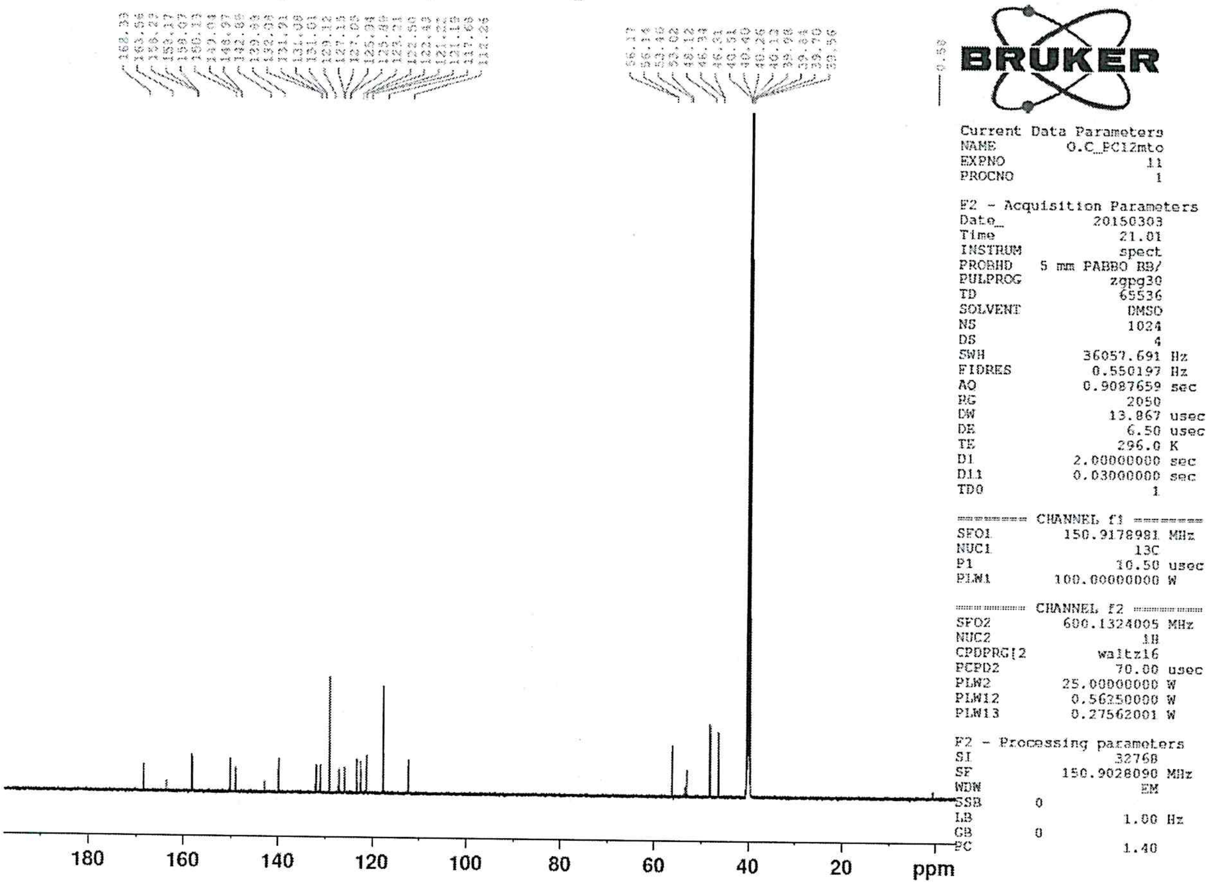

Data 31. Mass spectral analysis of compound **10**

Qualitative Analysis Report

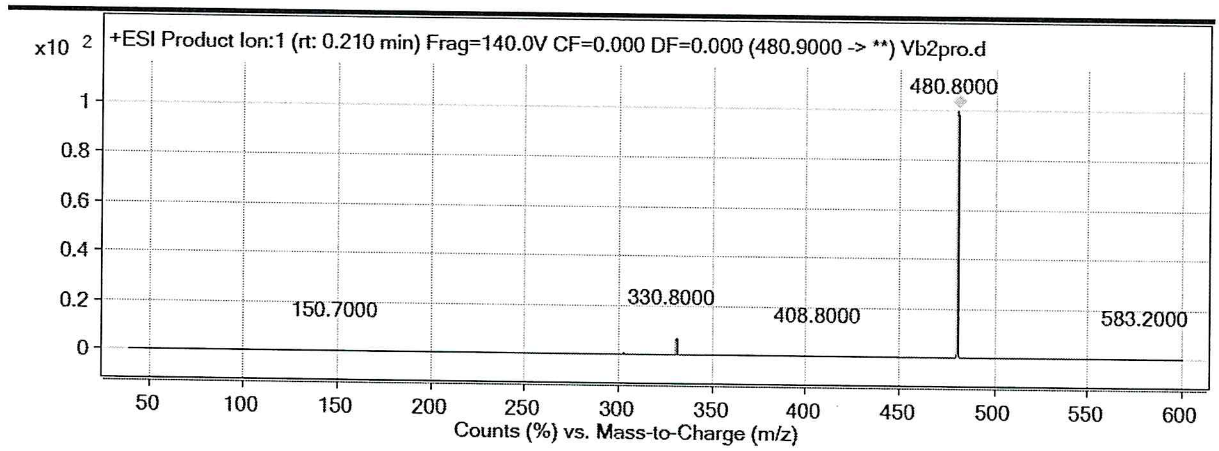

Peak List

m/z	z	Abund
149.5	2	54.76
150.7	2	94.6
179.9		54.5
180.6	2	54.62
190.7	2	93.92
290.4	2	56.7
302.7	2	446.1
328.6	2	54.6
330.8		5004.22
480.8		76619.34

Data 32. ^{1}H-NMR spectral analysis of compound **11**

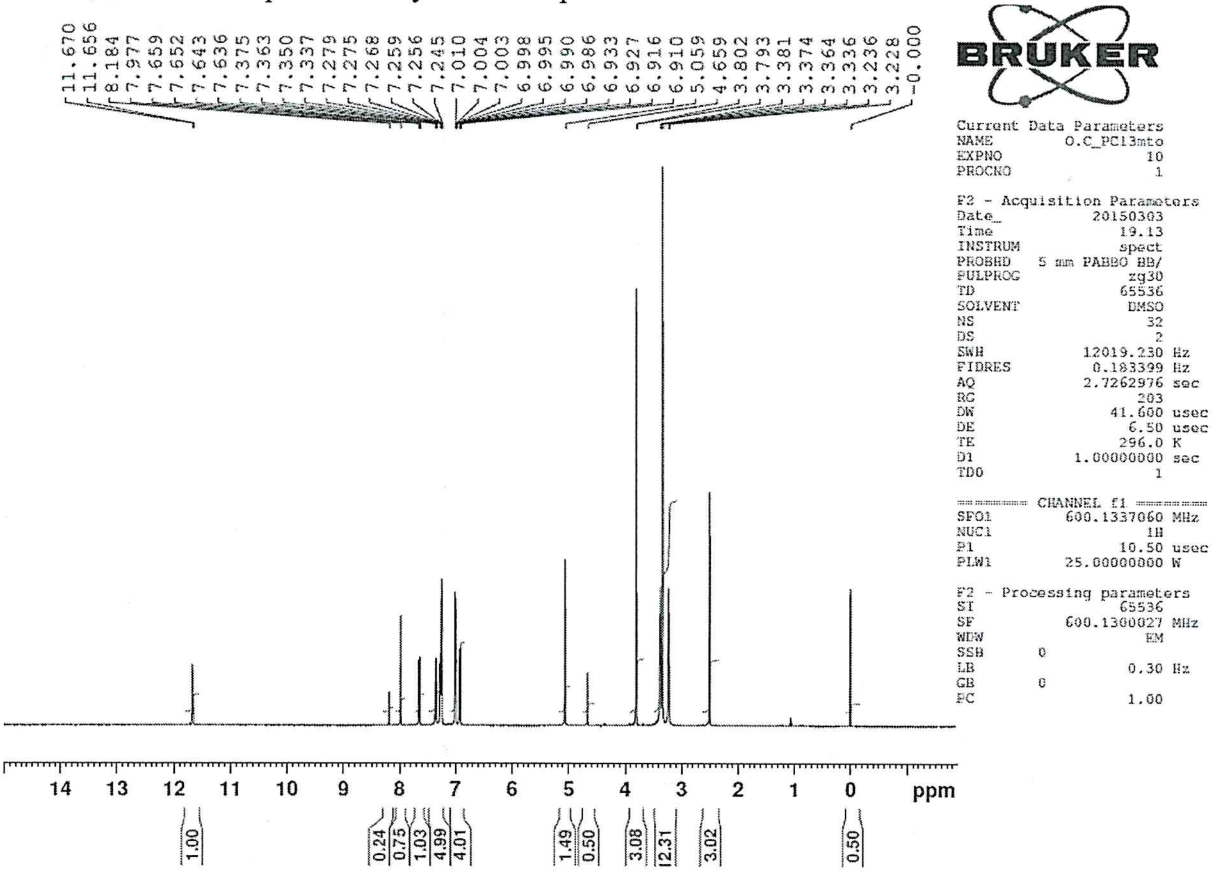

Data 33. ^{13}C-NMR spectral analysis of compound **11**

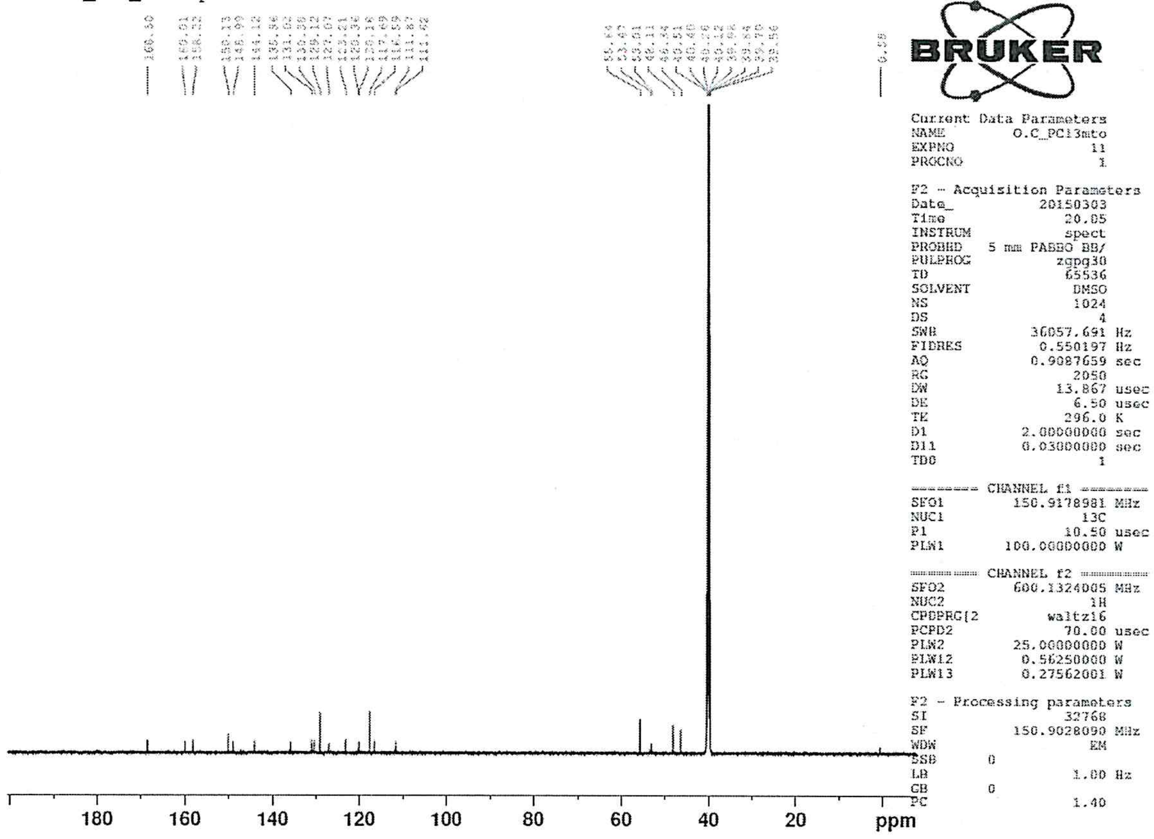

Data 34. Mass spectral analysis of compound **11**

Qualitative Analysis Report

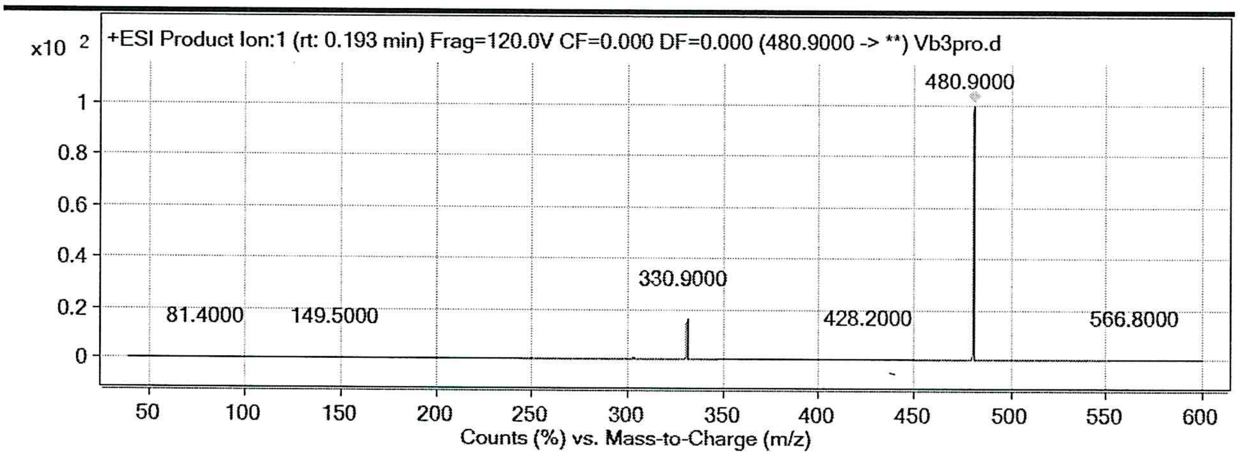

Peak List

m/z	z	Abund
81.4	2	59.4
131.8		53.64
138.3	2	60.9
149.5	2	63.62
165.5	2	58.1
189.9		54.7
274.2		56.72
302.7	2	540.44
330.9		10467.06
480.9		67117.24

Data 35. ^{1}H-NMR spectral analysis of compound **12**

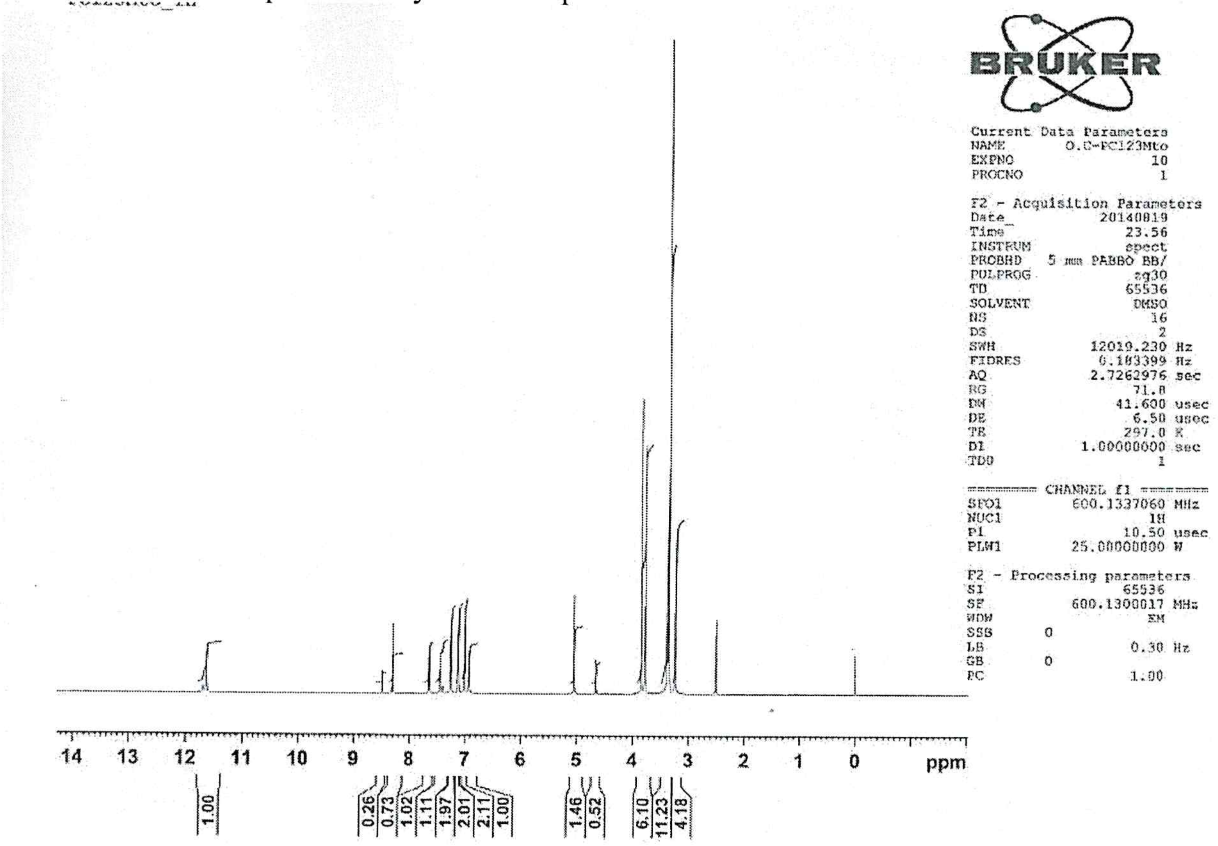

Data 36. ^{13}C-NMR spectral analysis of compound **12**

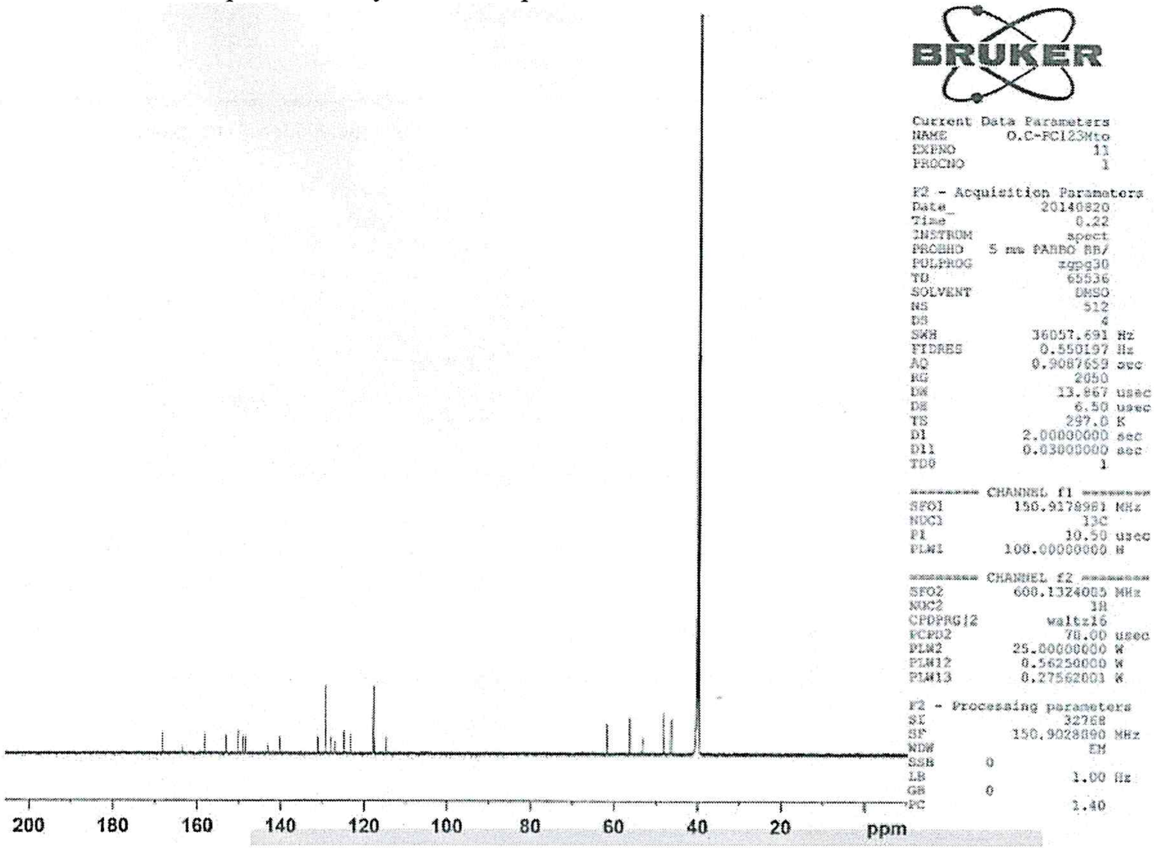

Data 37. Mass spectral analysis of compound **12**

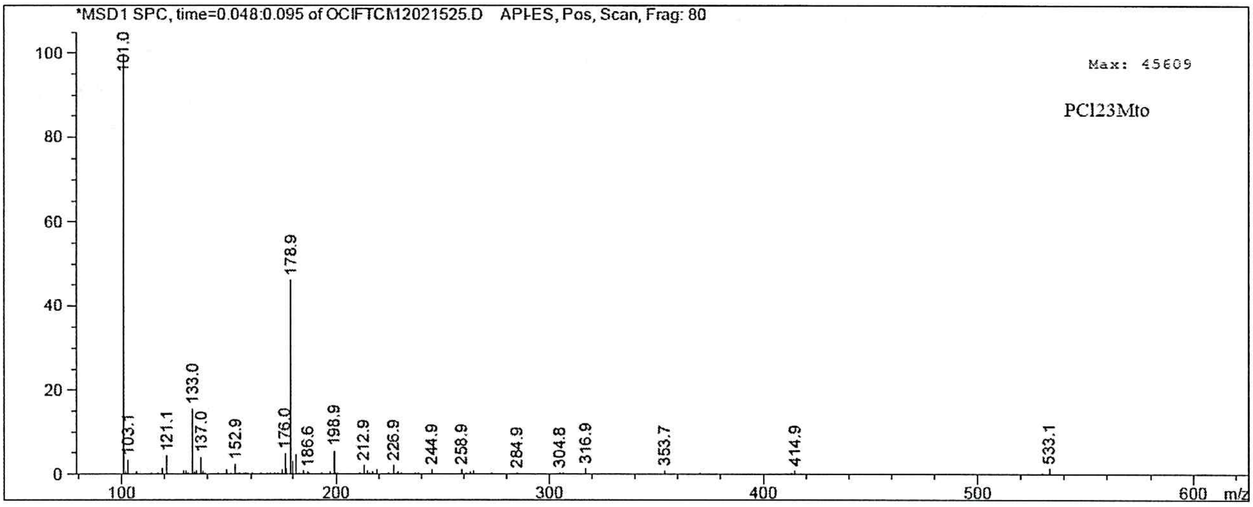

Data 38. ^{1}H-NMR spectral analysis of compound **13**

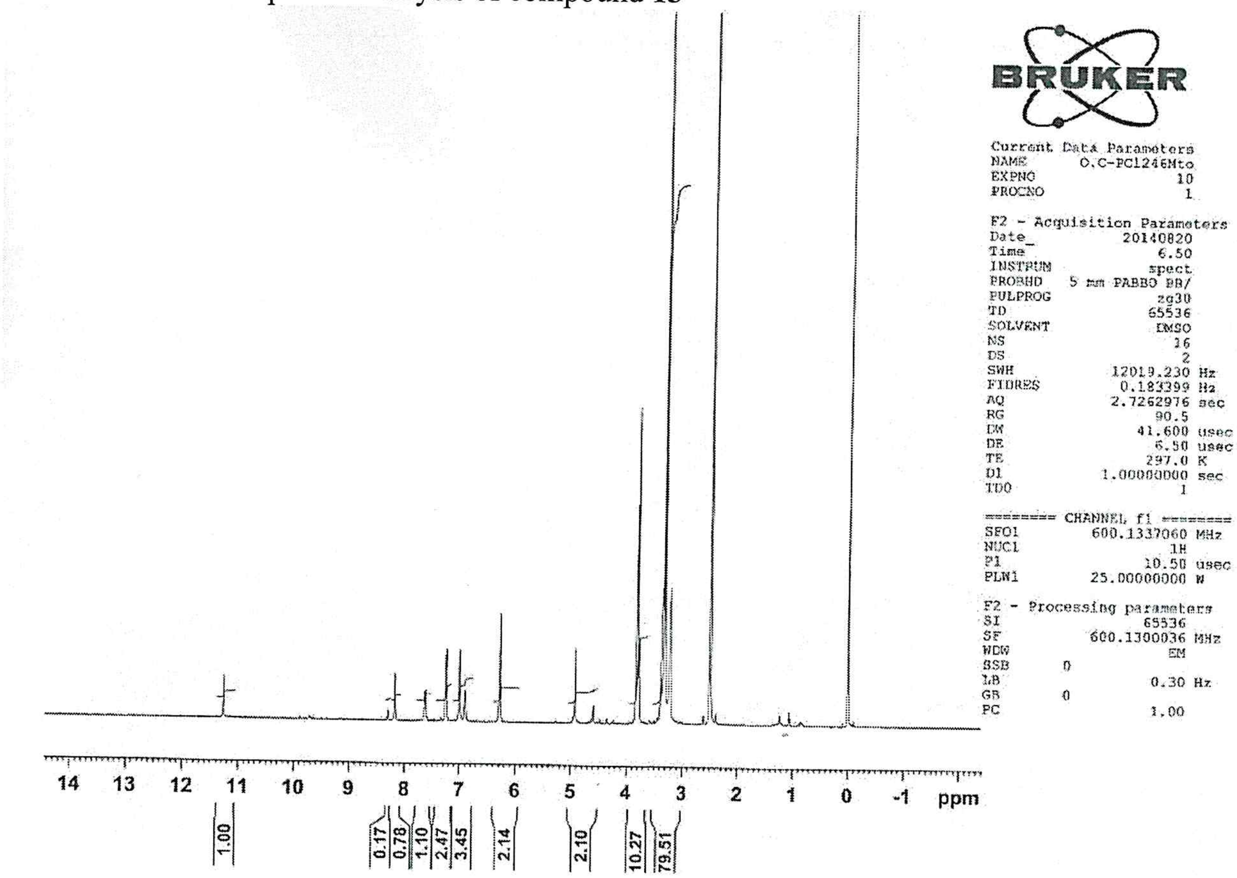

Data 39. ^{13}C-NMR spectral analysis of compound **13**

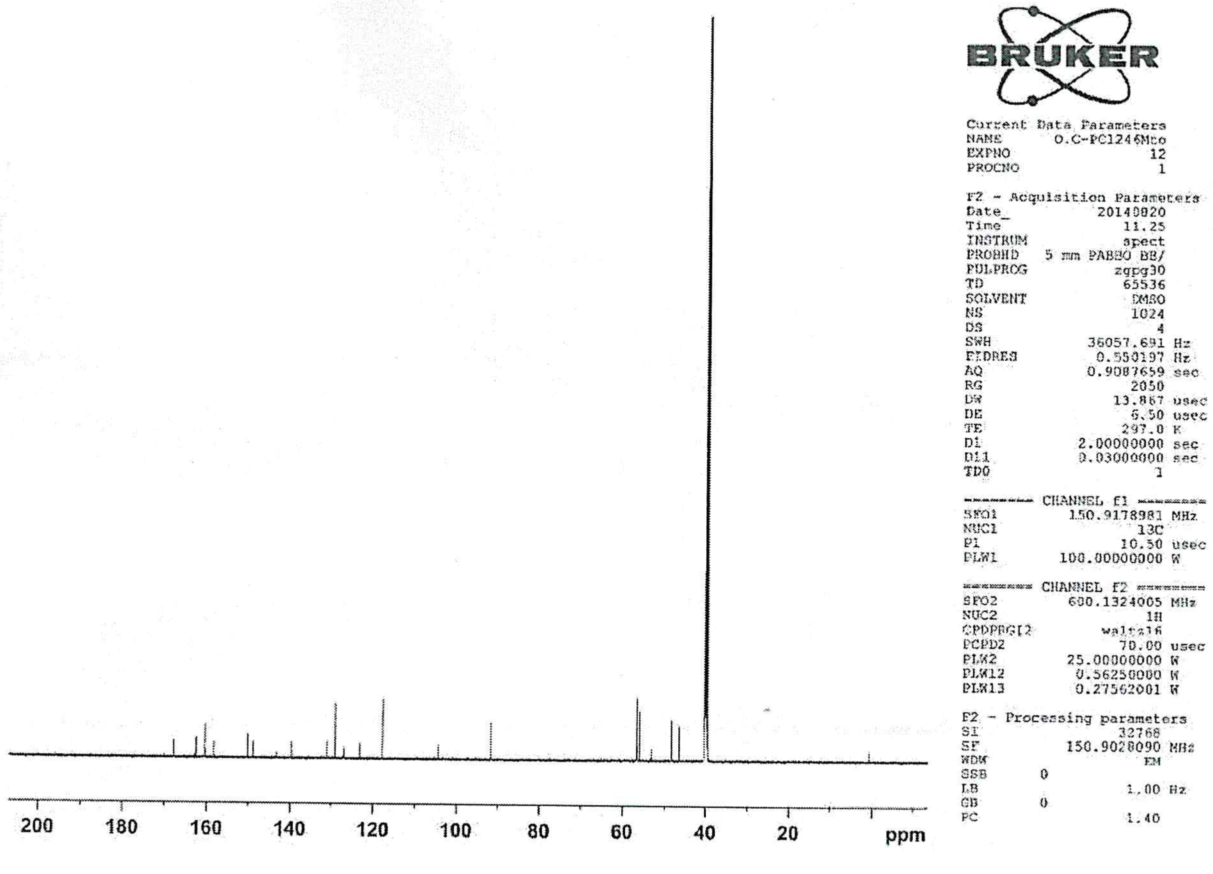

Data 40. Mass spectral analysis of compound **13**

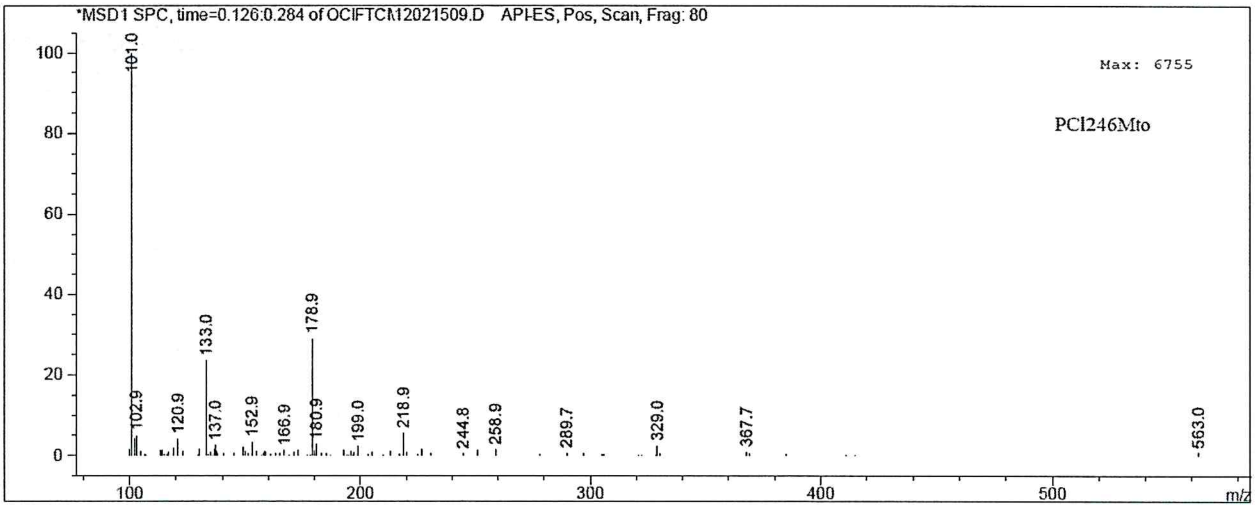

Data 41. ^{1}H-NMR spectral analysis of compound **14**

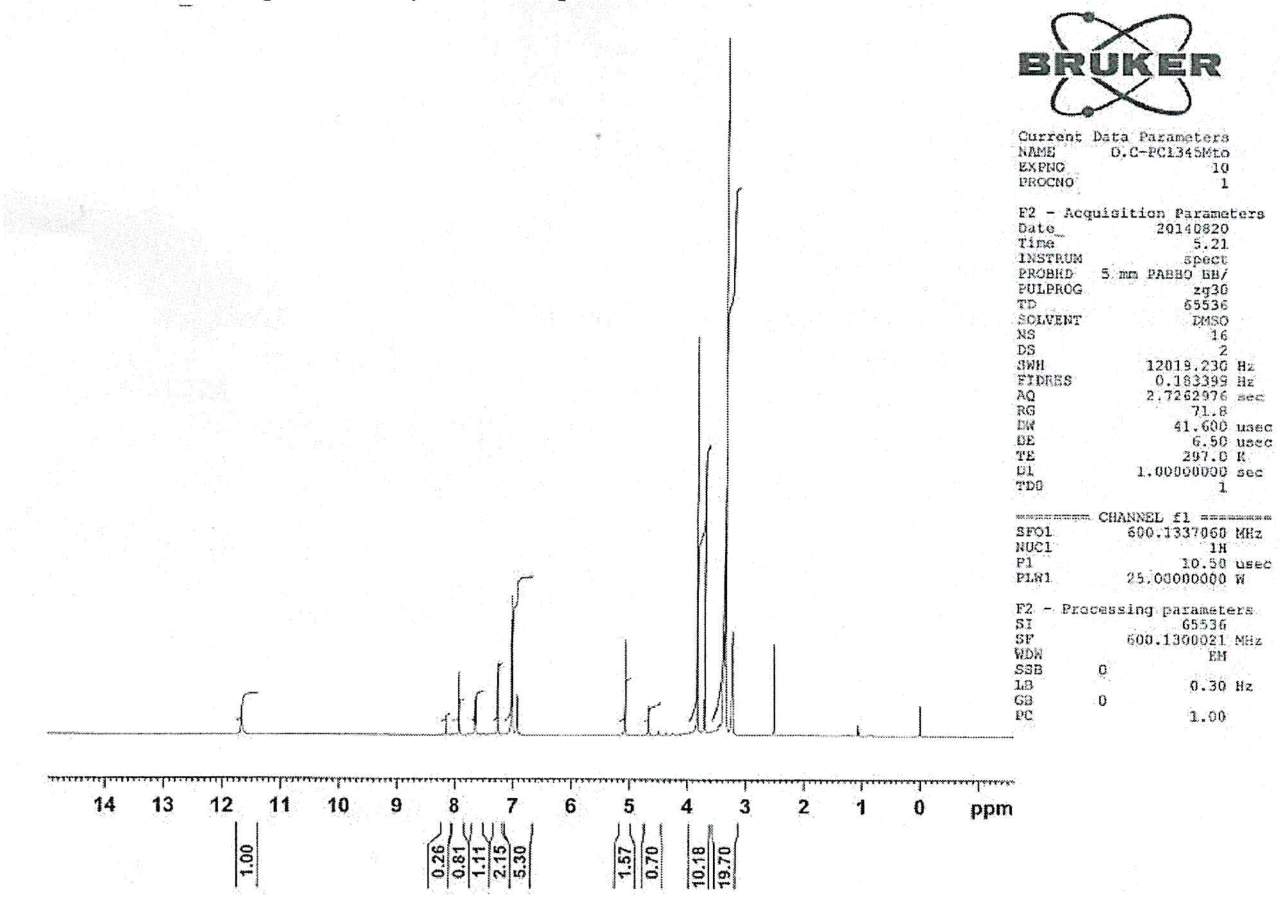

Data 42. ^{13}C-NMR spectral analysis of compound **14**

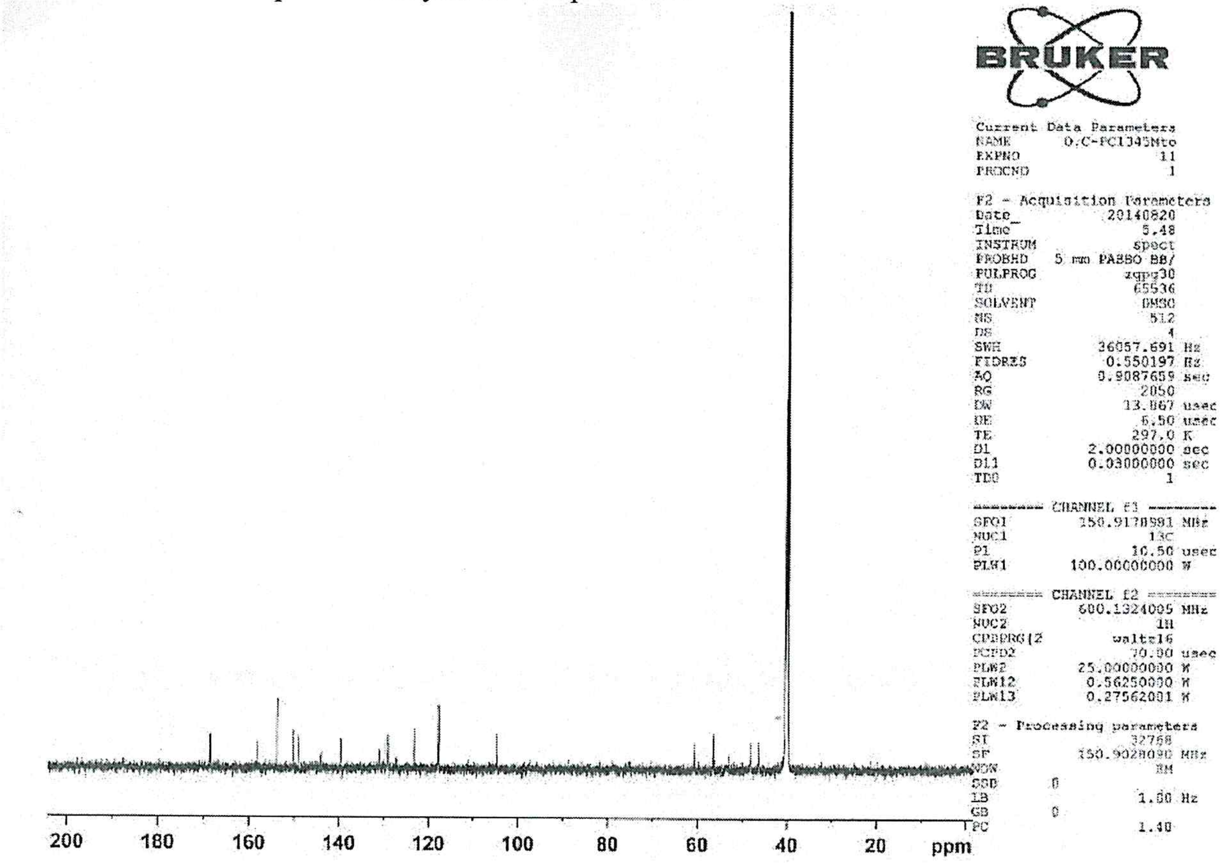

Data 43. Mass spectral analysis of compound **14**

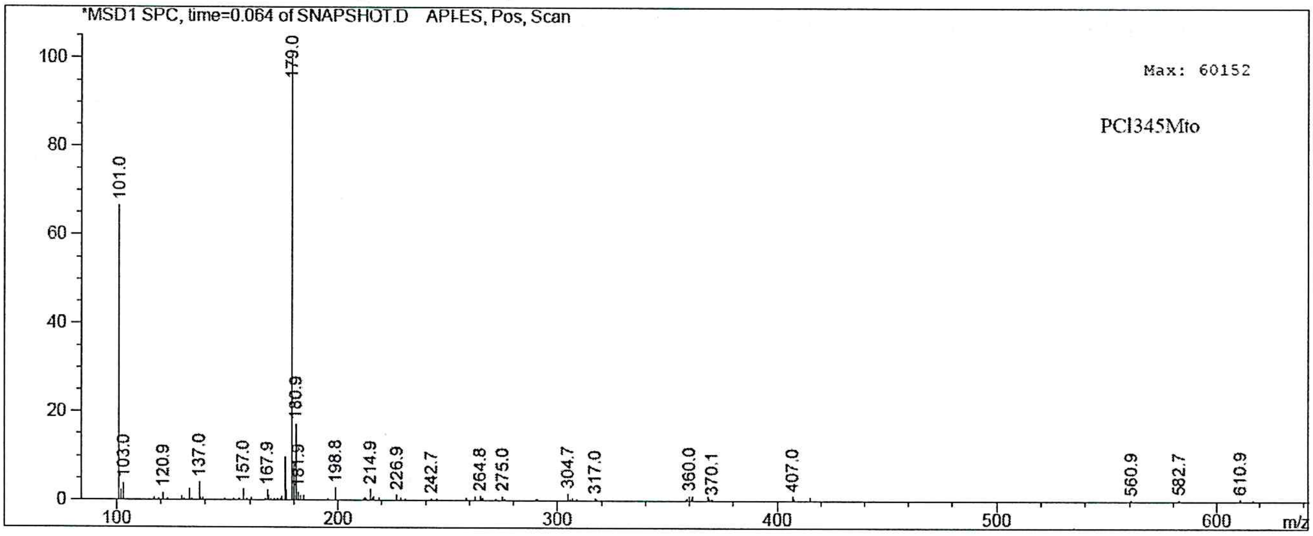

Data 44. ^{1}H-NMR spectral analysis of compound **15**

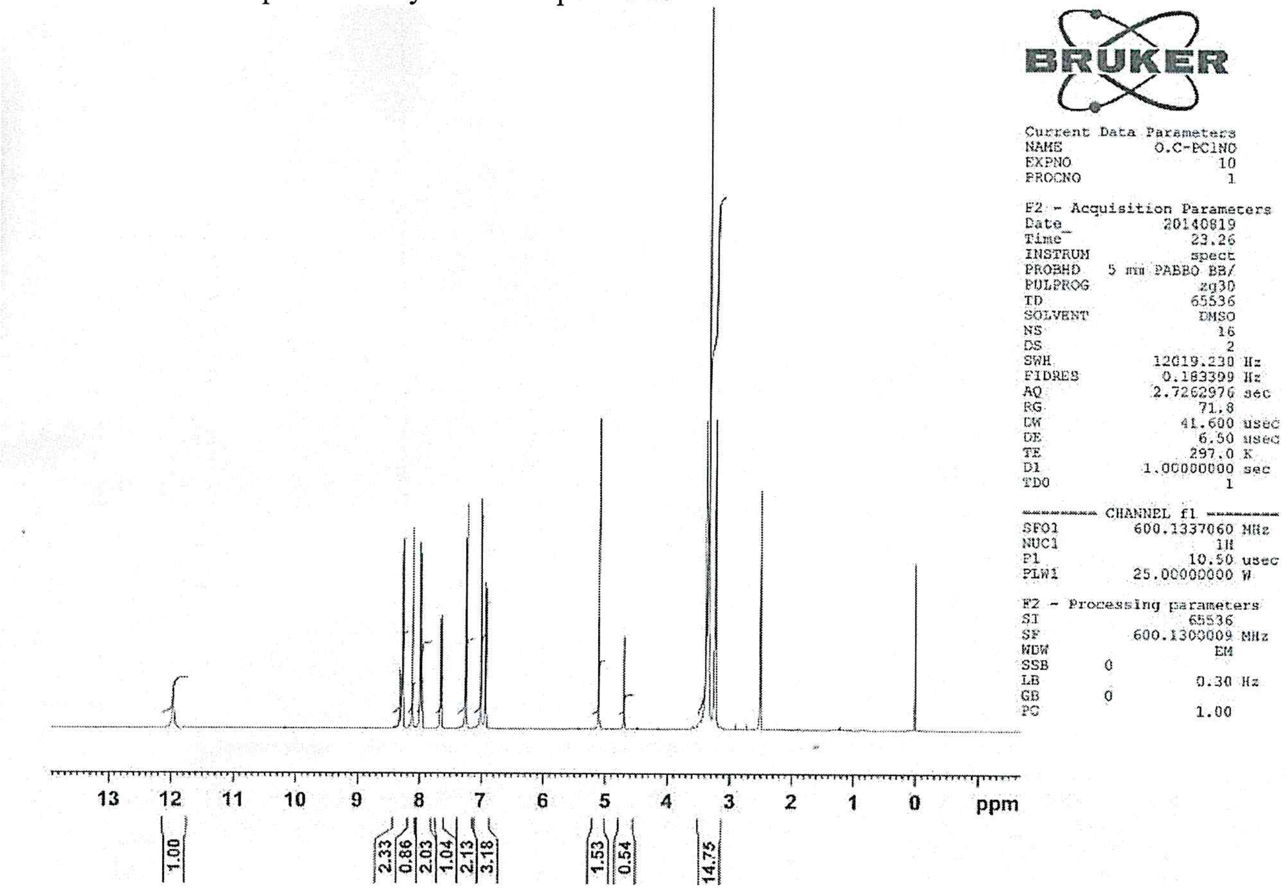

Data 45. ^{13}C-NMR spectral analysis of compound **15**

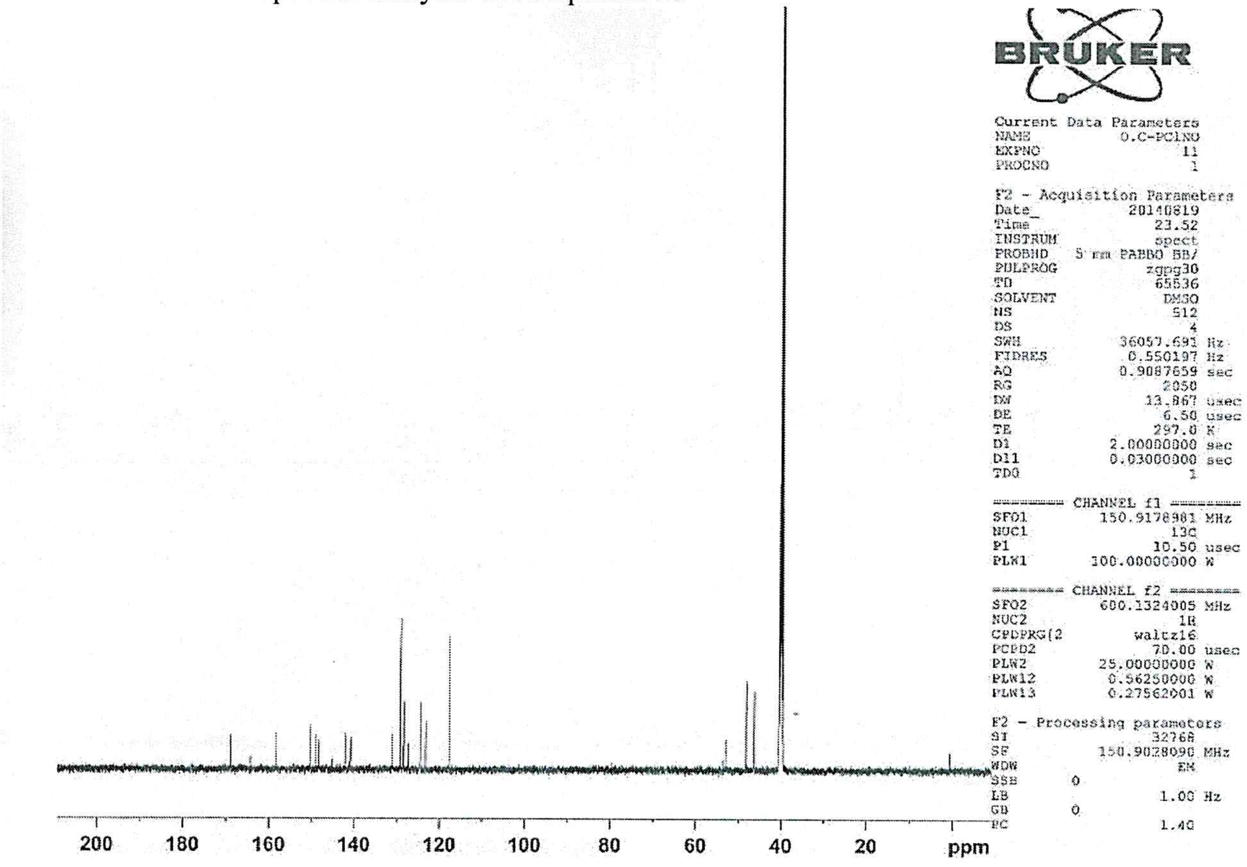

Data 46. Mass spectral analysis of compound **15**

Qualitative Analysis Report

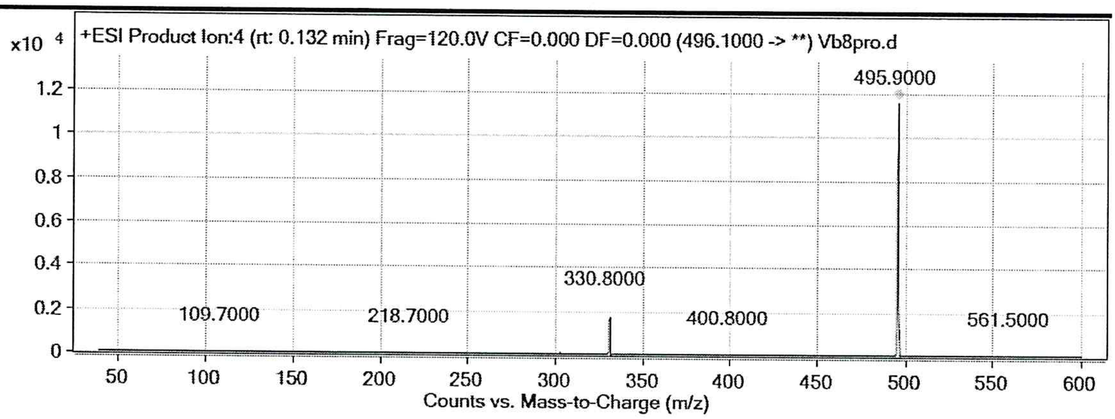

Peak List

m/z	z	Abund
109.7	2	49.88
150.1		49.12
275.5	2	50.56
302.6	2	127.04
327.5	2	53.7
330.8		1760.5
353.6	2	48.98
486		49.1
495.9		11614.72
497		71.86

Data 47. ^{1}H-NMR spectral analysis of compound **16**

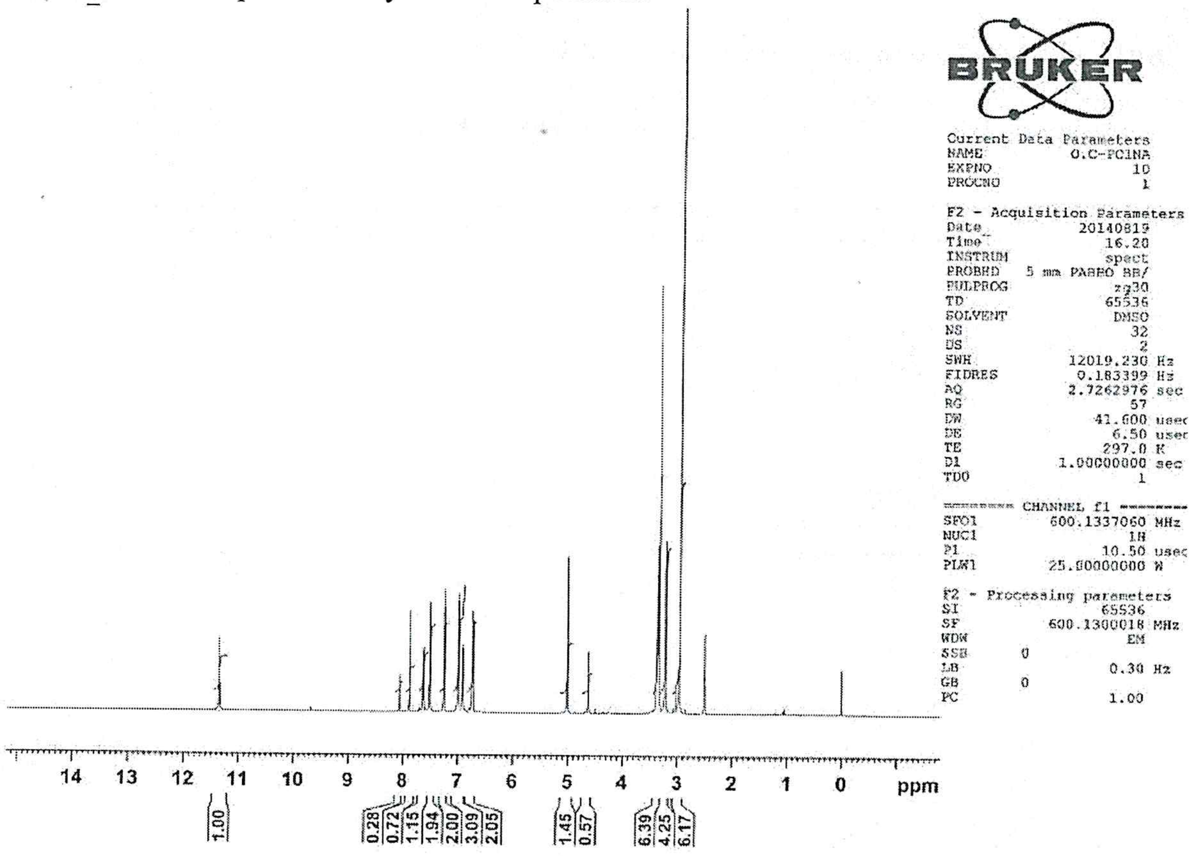

Data 48. ^{13}C-NMR spectral analysis of compound **16**

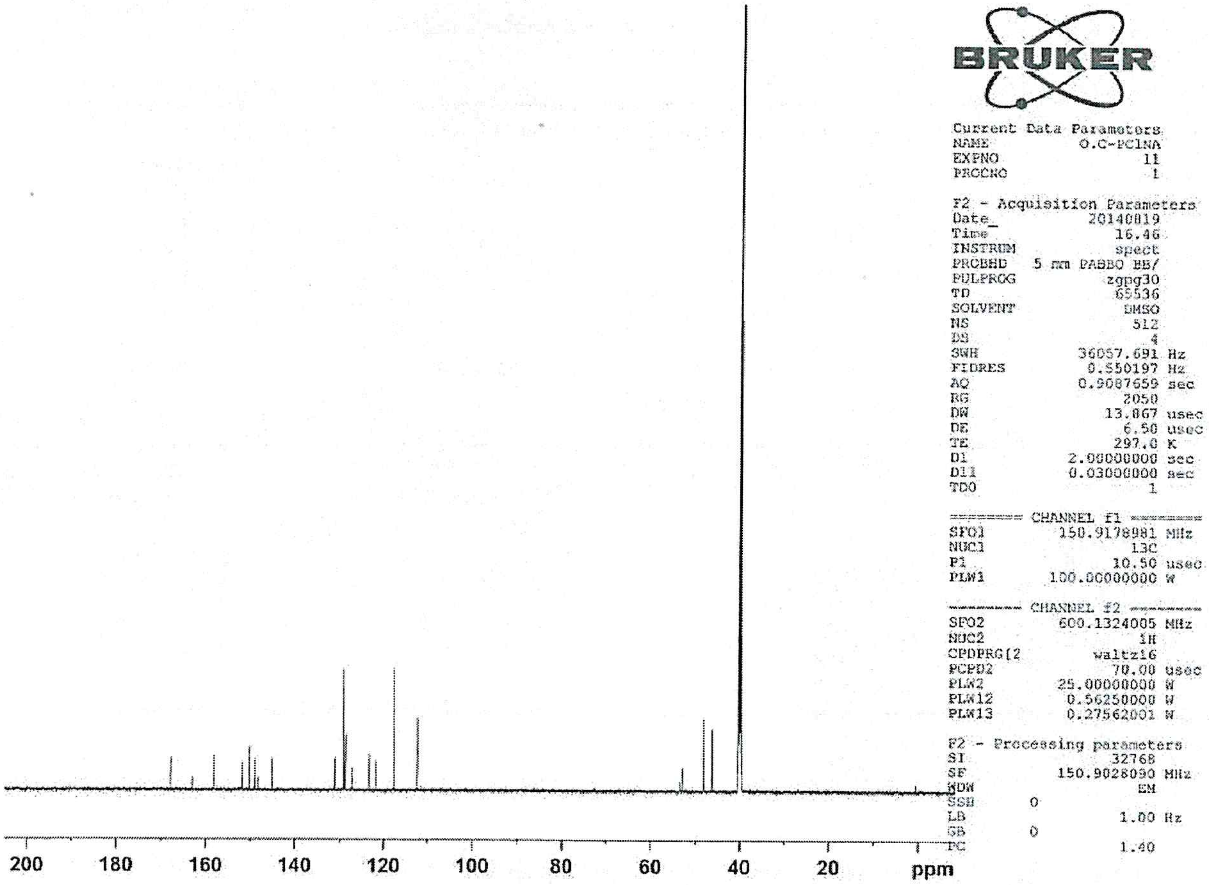

Data 49. Mass spectral analysis of compound **16**

Qualitative Analysis Report

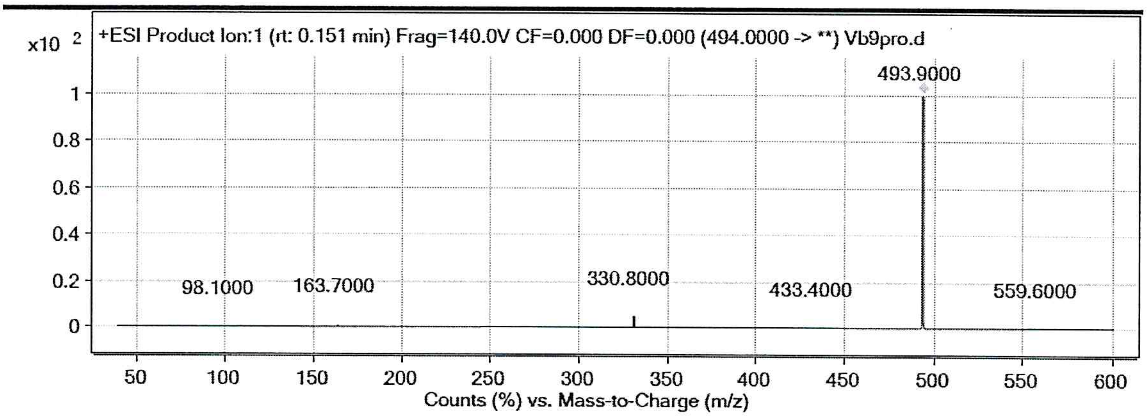

Peak List

m/z	z	Abund
152.9		53.56
163.7	2	509.82
175.5	2	76.96
203.2		56.4
204.2		75.58
221		58.16
302.6	2	289.62
330.8		3890.98
493.9		78139.02
495.4		54.96

Data 50. ^{1}H-NMR spectral analysis of compound **17**

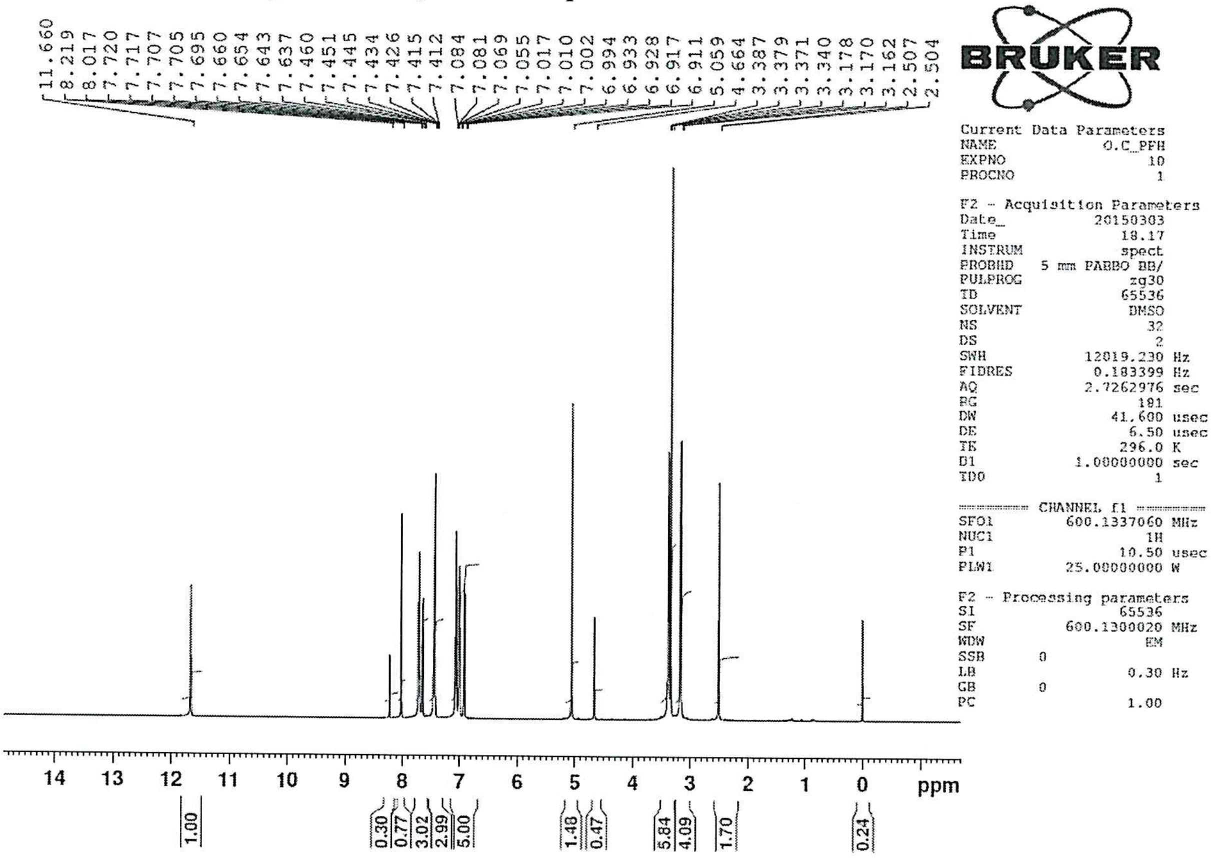

Data 51. ^{13}C-NMR spectral analysis of compound **17**

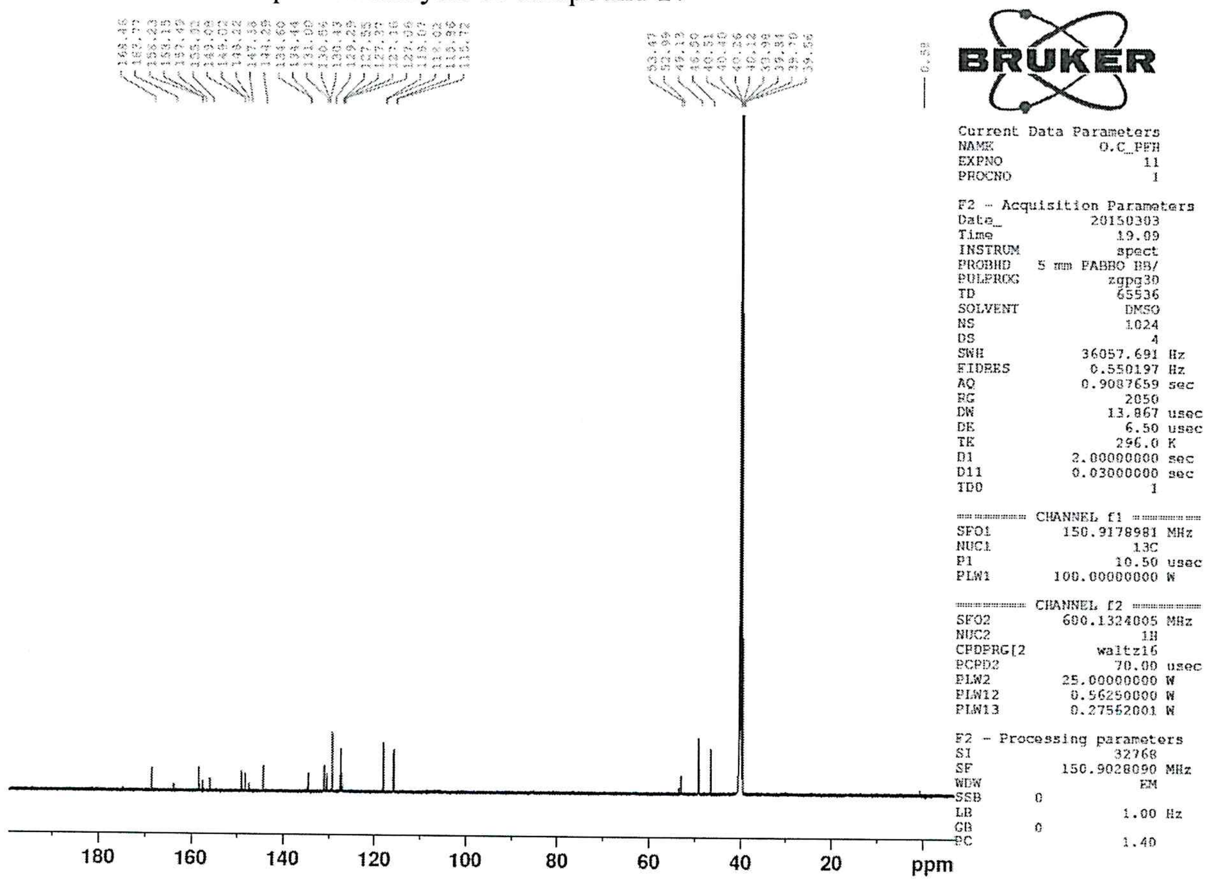

Data 52. Mass spectral analysis of compound **17**

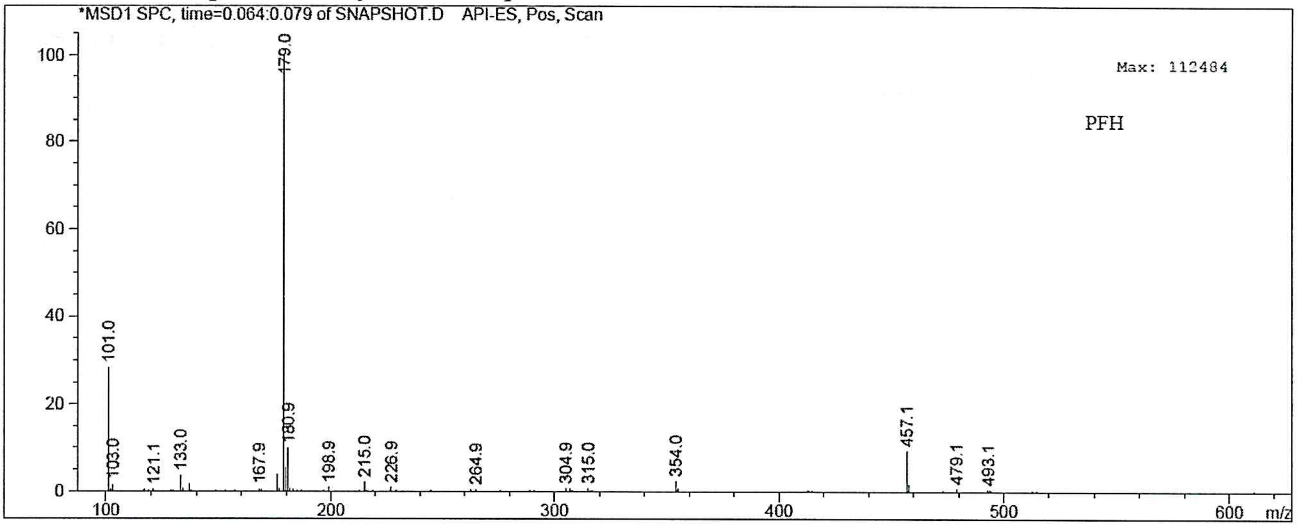

Data 53. ^{1}H-NMR spectral analysis of compound **18**

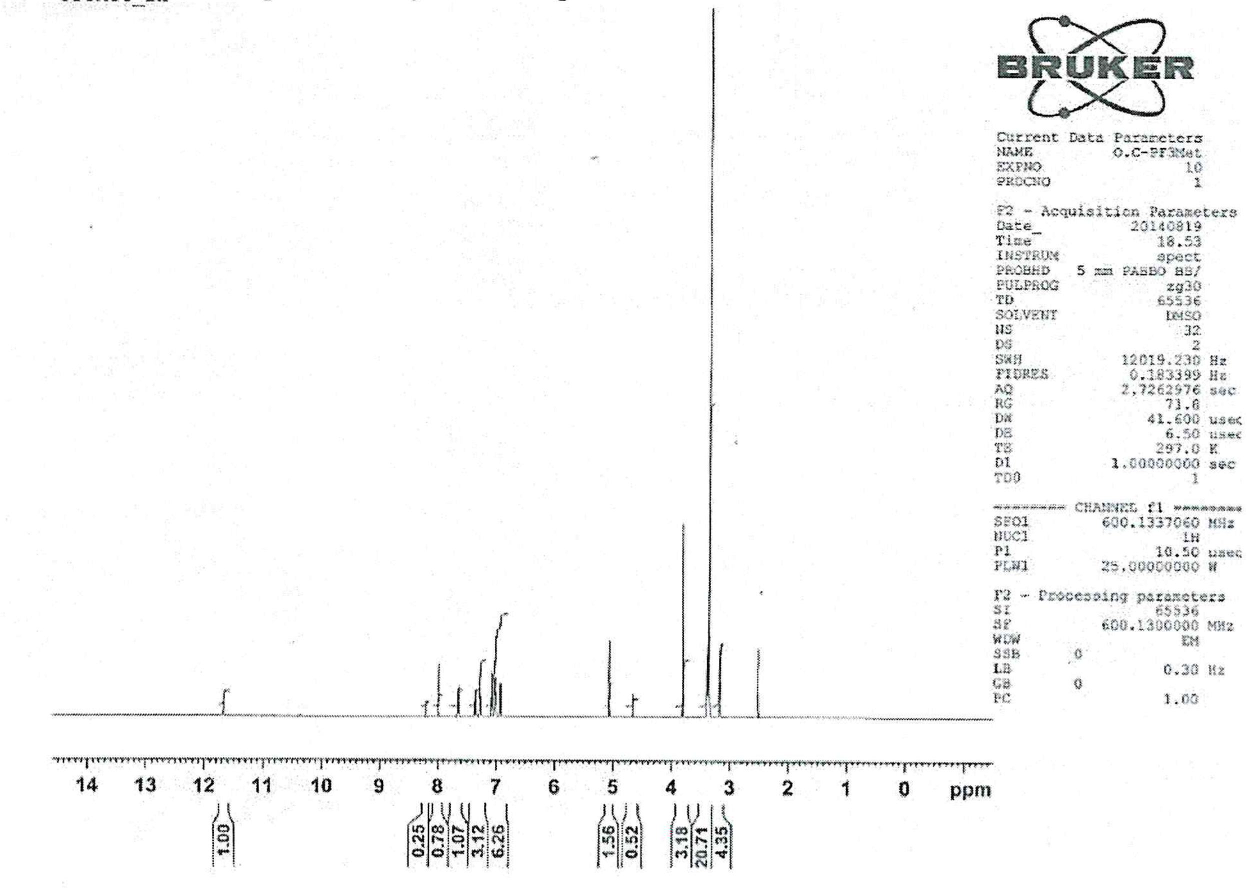

Data 54. ^{13}C-NMR spectral analysis of compound **18**

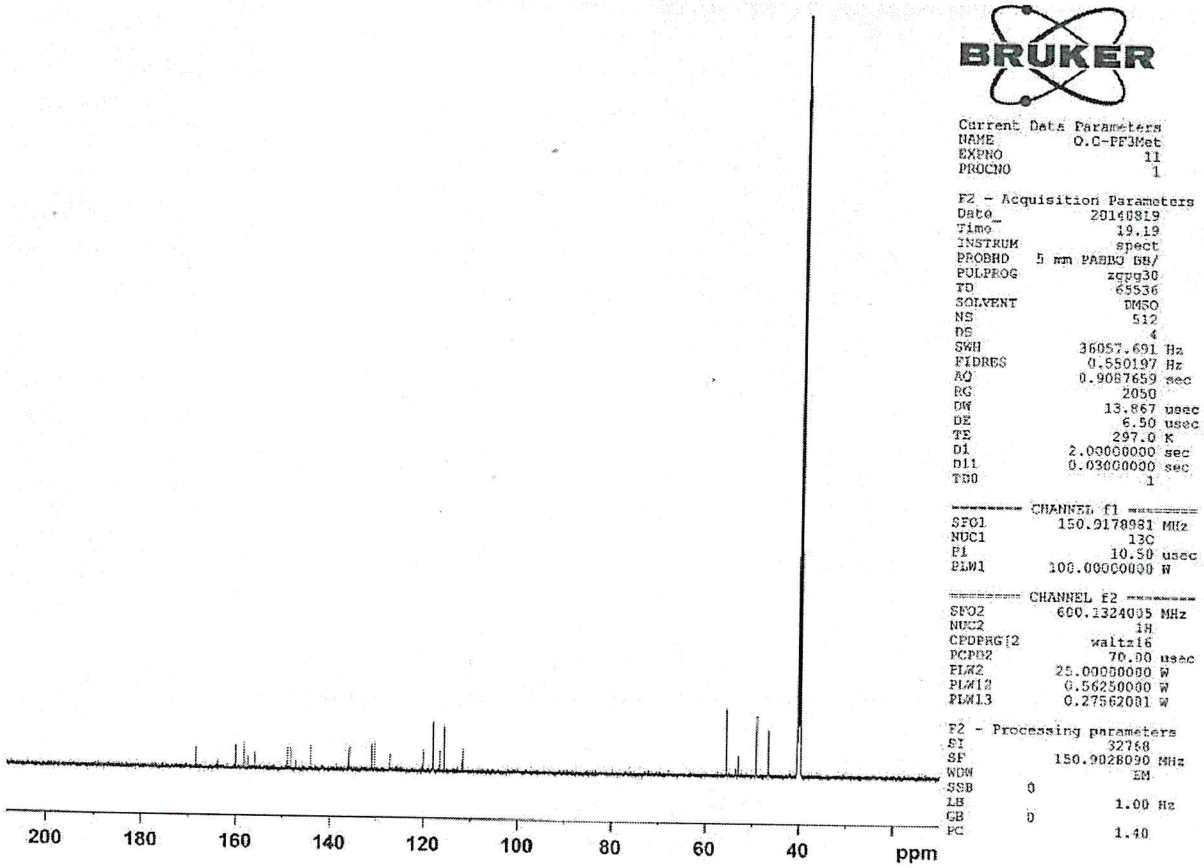

Data 55. Mass spectral analysis of compound **18**

Qualitative Analysis Report

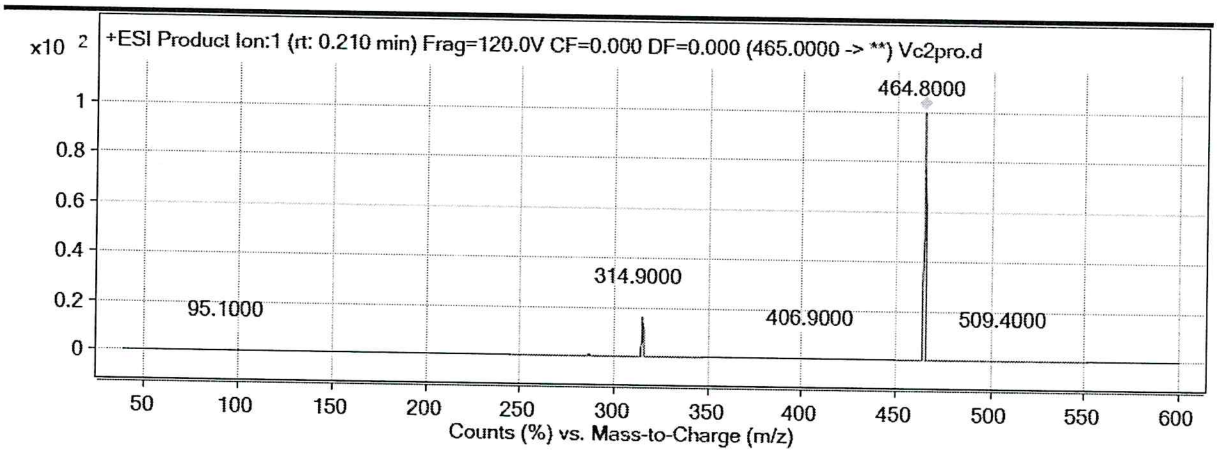

Peak List

m/z	z	Abund
94		56.28
95.1		75.34
96.9		56.44
121.6	2	60.74
137		72.66
149.8		68.82
162.2		54.92
286.6	2	1324.7
314.9		26129.38
464.8		161148.52

Data 56. ^{1}H-NMR spectral analysis of compound **19**

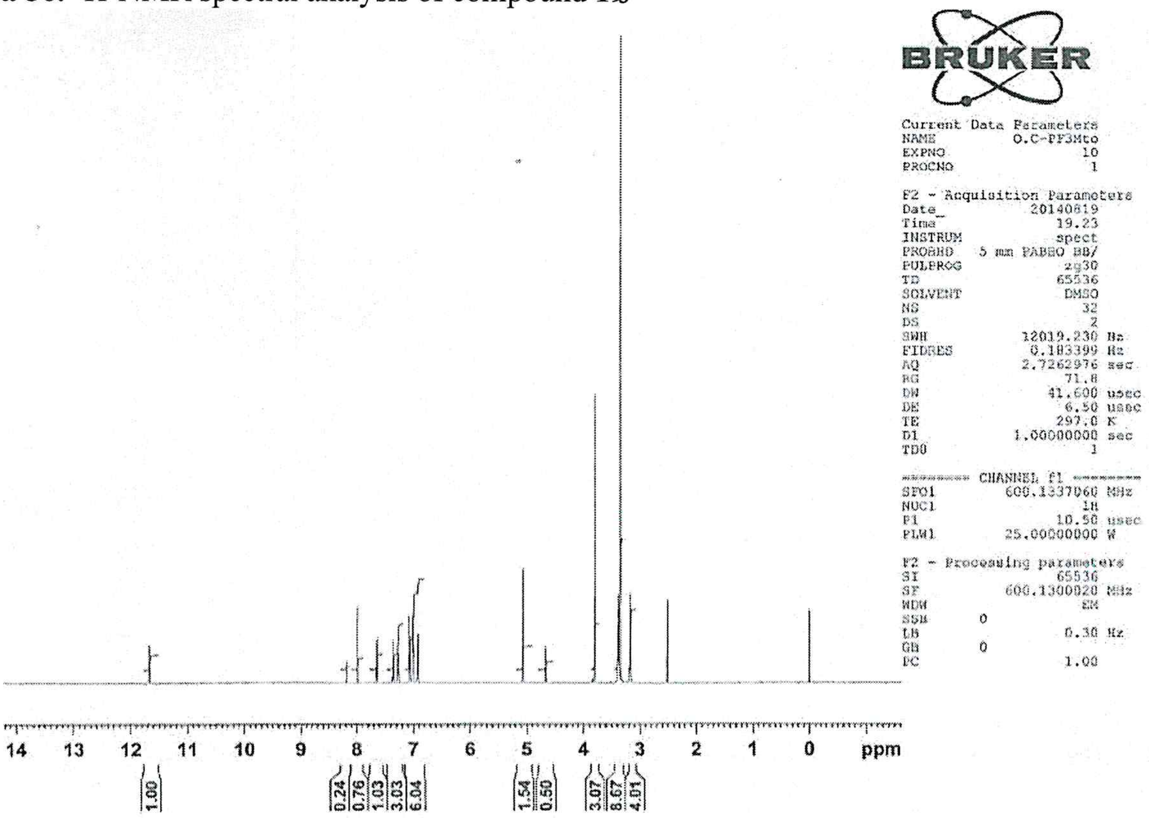

Data 57. ^{13}C-NMR spectral analysis of compound **19**

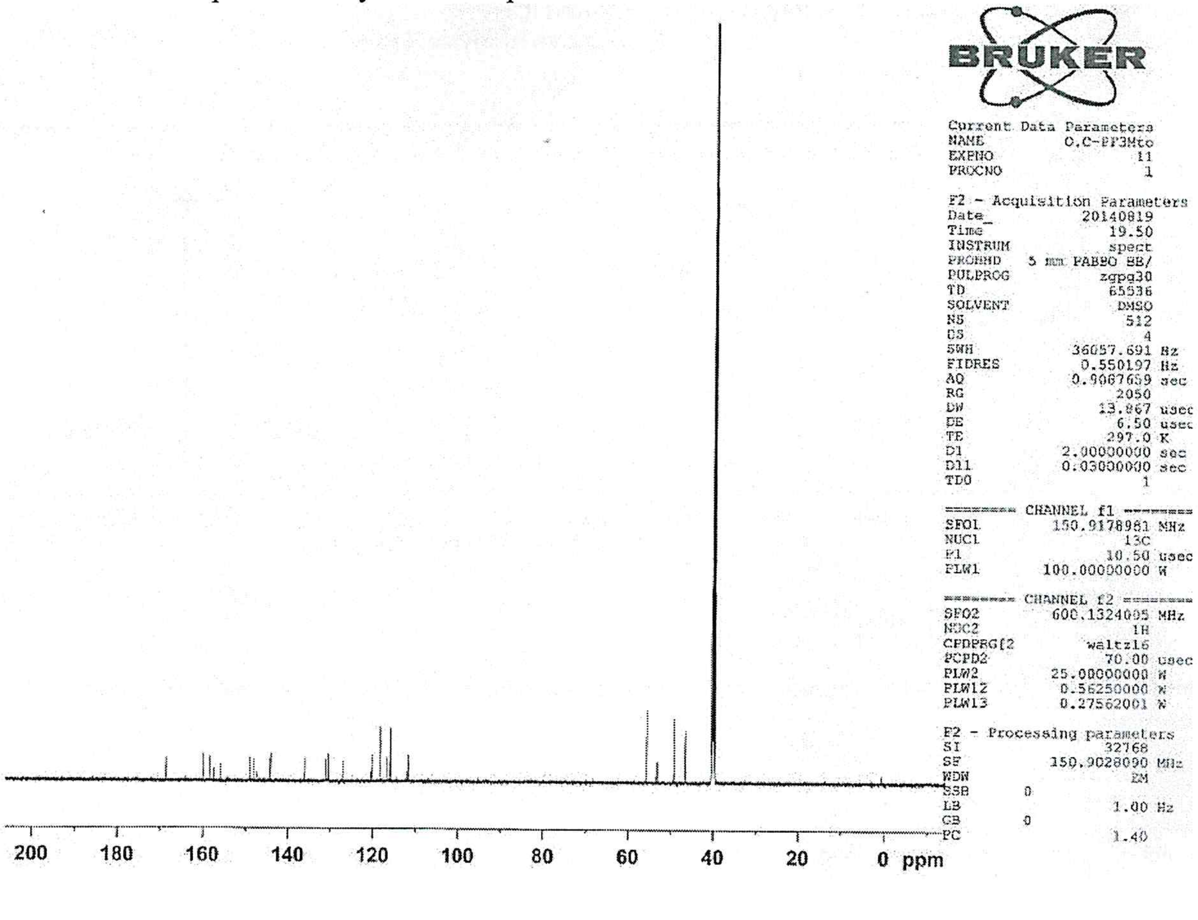

Data 58. Mass spectral analysis of compound **19**

Qualitative Analysis Report

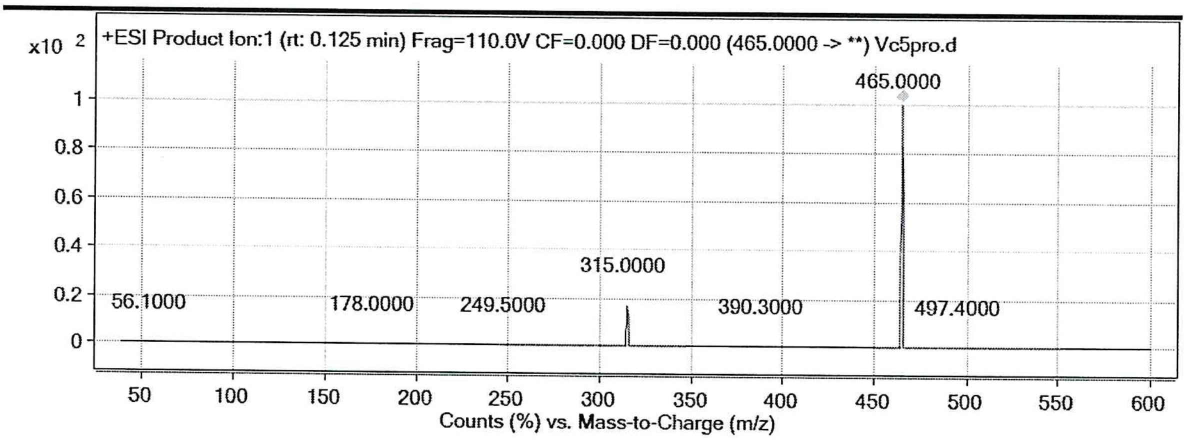

Peak List

m/z	z	Abund
56.1		56.24
94.1		51.08
122.2		51.12
178		58.2
249.5	2	59.02
272.2		54.22
286.5	2	70.4
315		3012.14
390.3		53.62
465		18277.52

Data 59. ¹H-NMR spectral analysis of compound **20**

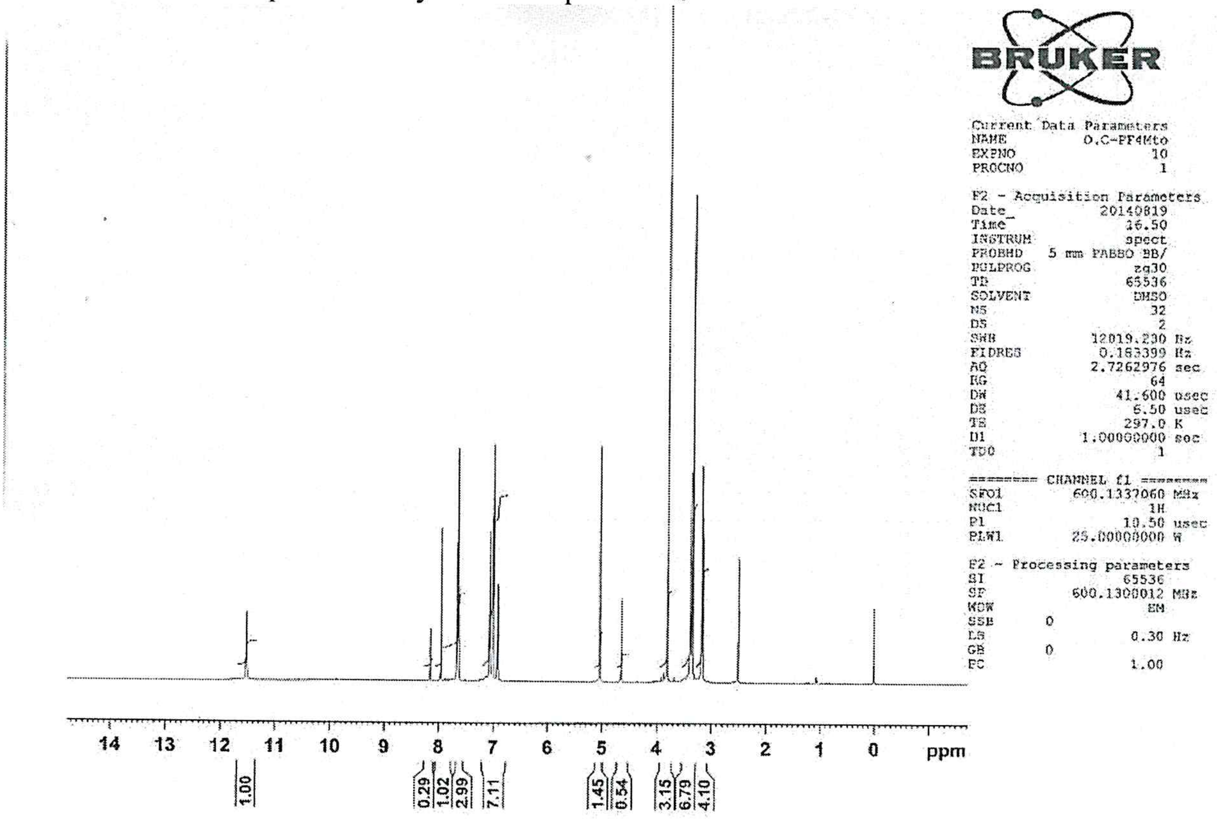

Data 60. ^{13}C-NMR spectral analysis of compound **20**

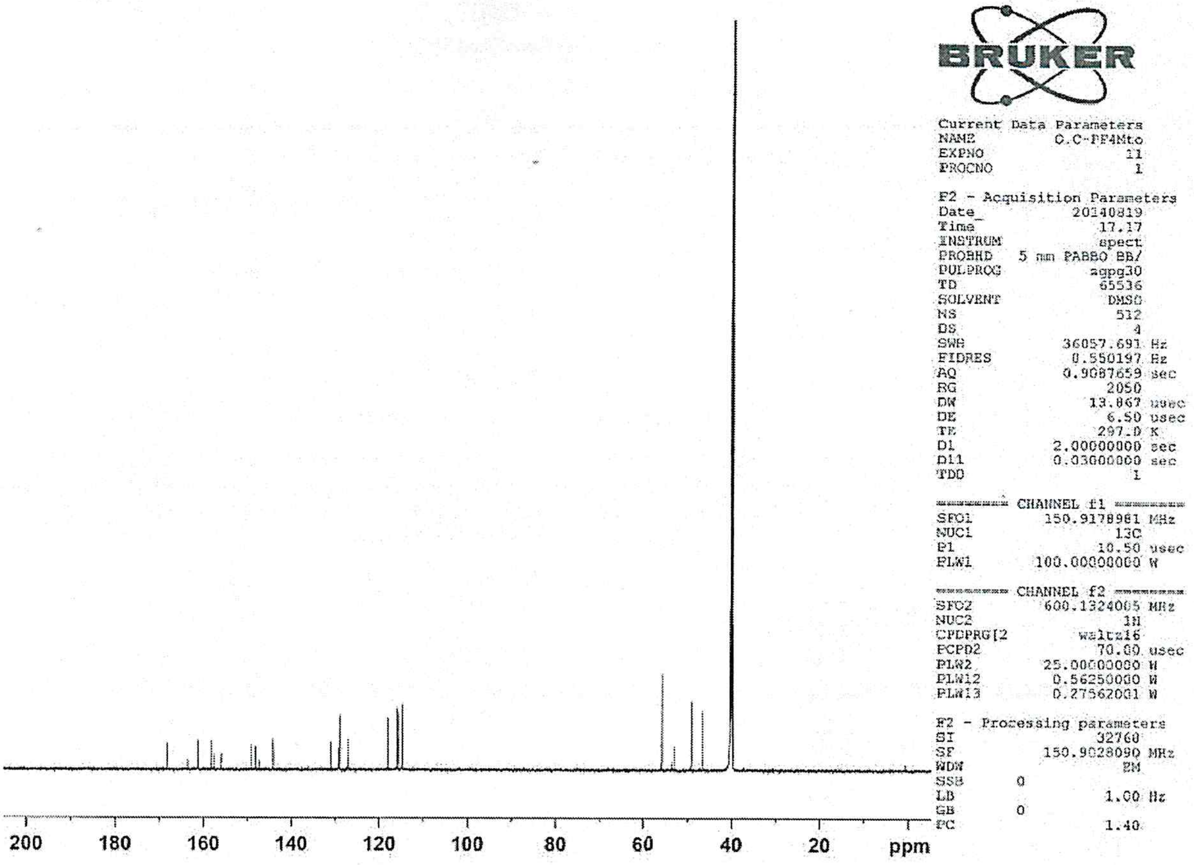

Data 61. Mass spectral analysis of compound **20**

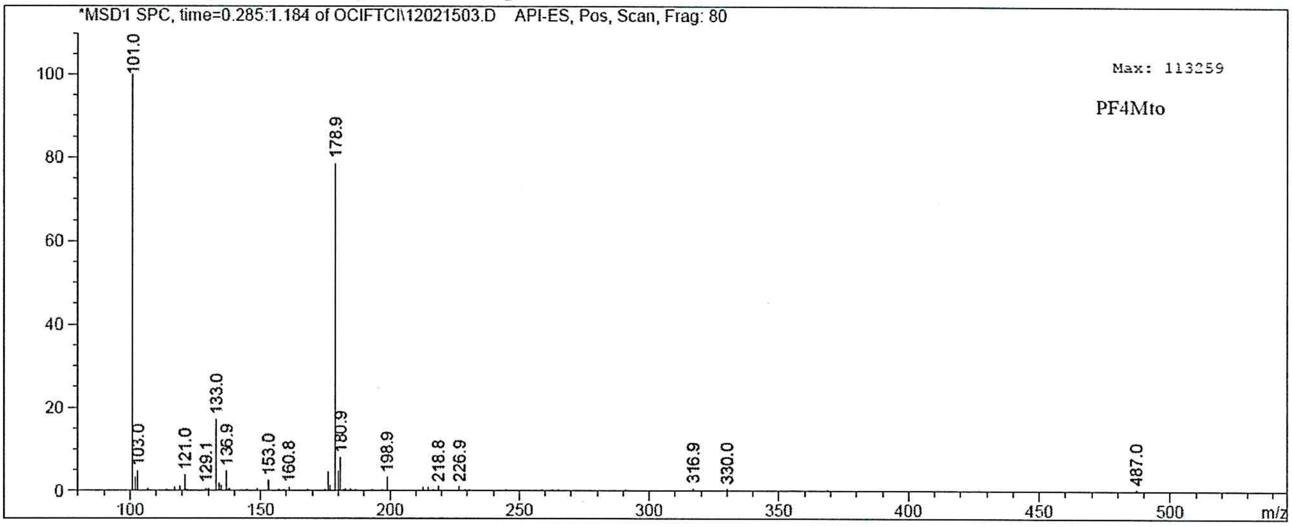

Data 62. ^{1}H-NMR spectral analysis of compound **21**

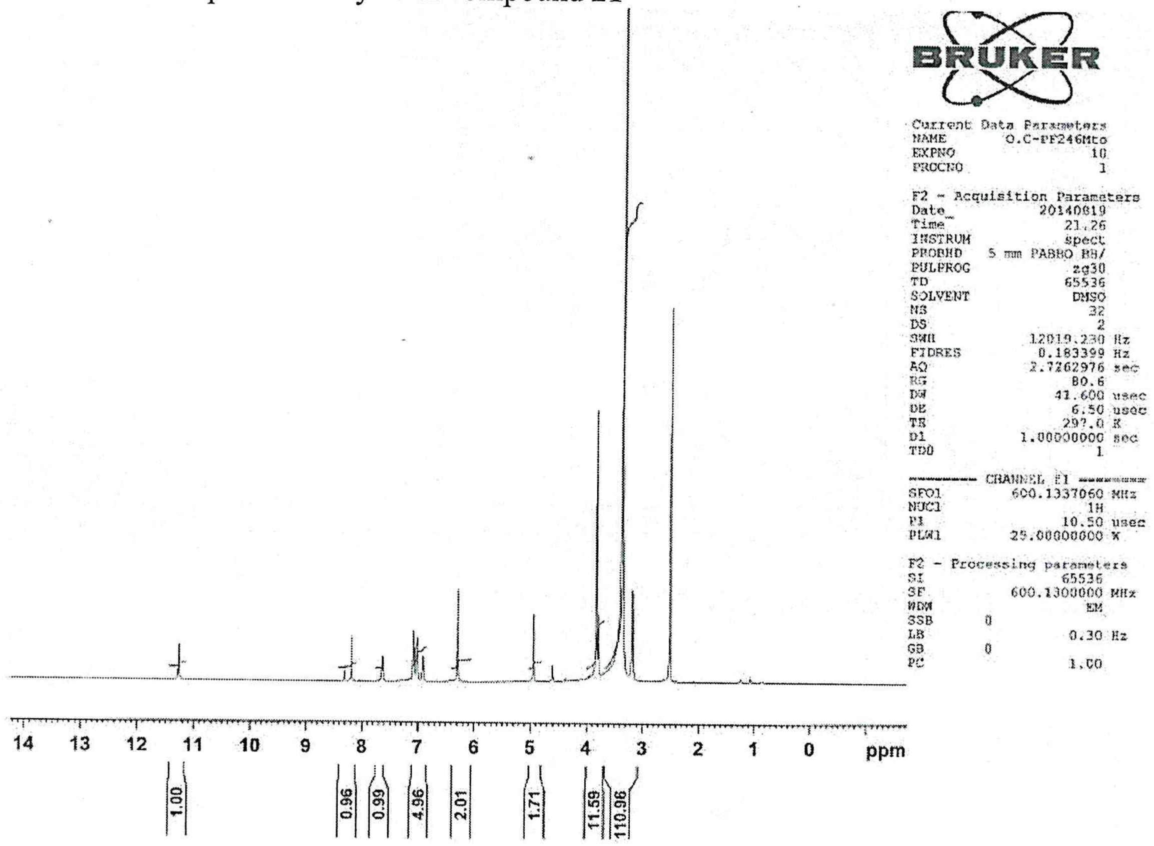

Data 63. ^{13}C-NMR spectral analysis of compound **21**

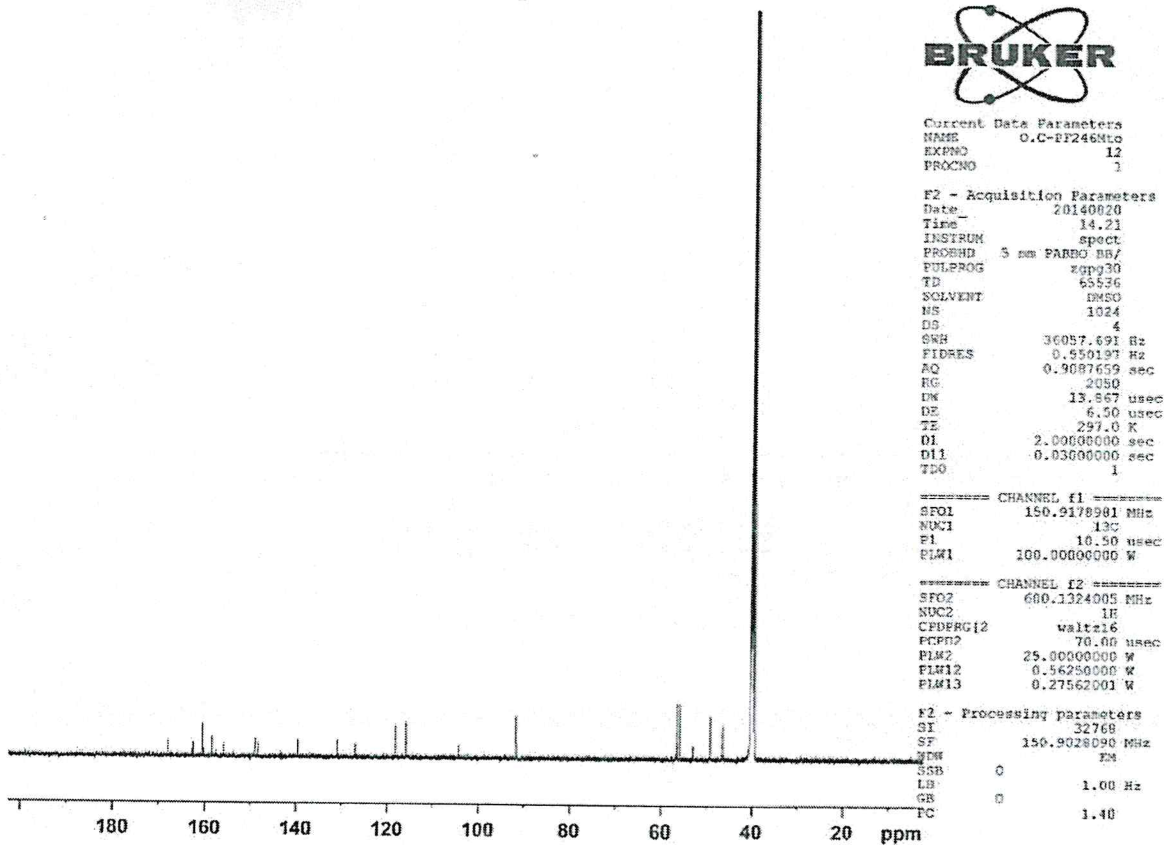

Data 64. Mass spectral analysis of compound **21**

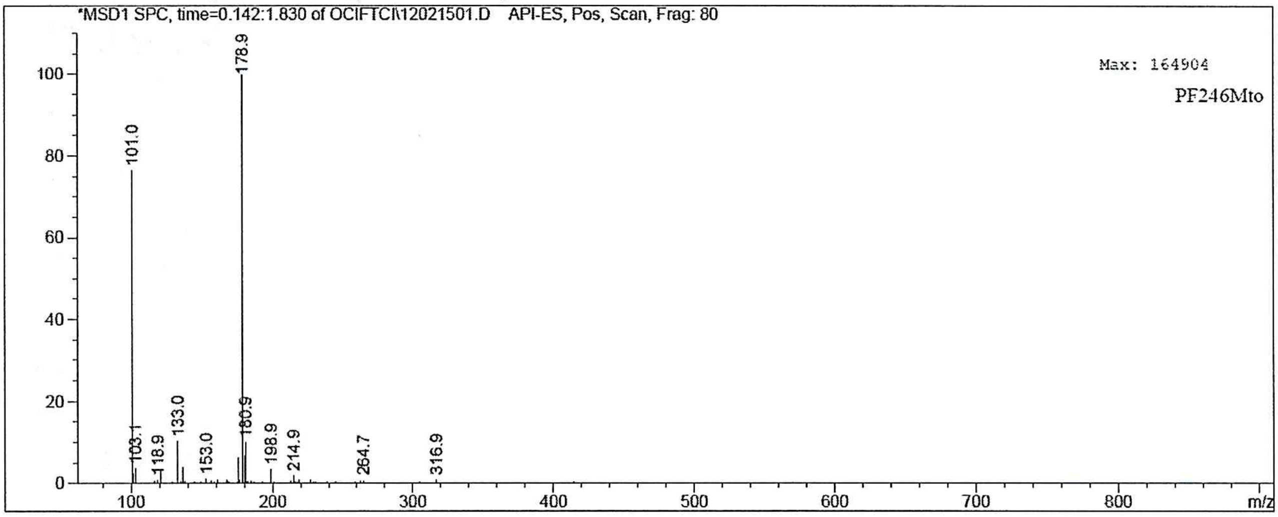

Data 65. ^{1}H-NMR spectral analysis of compound **22**

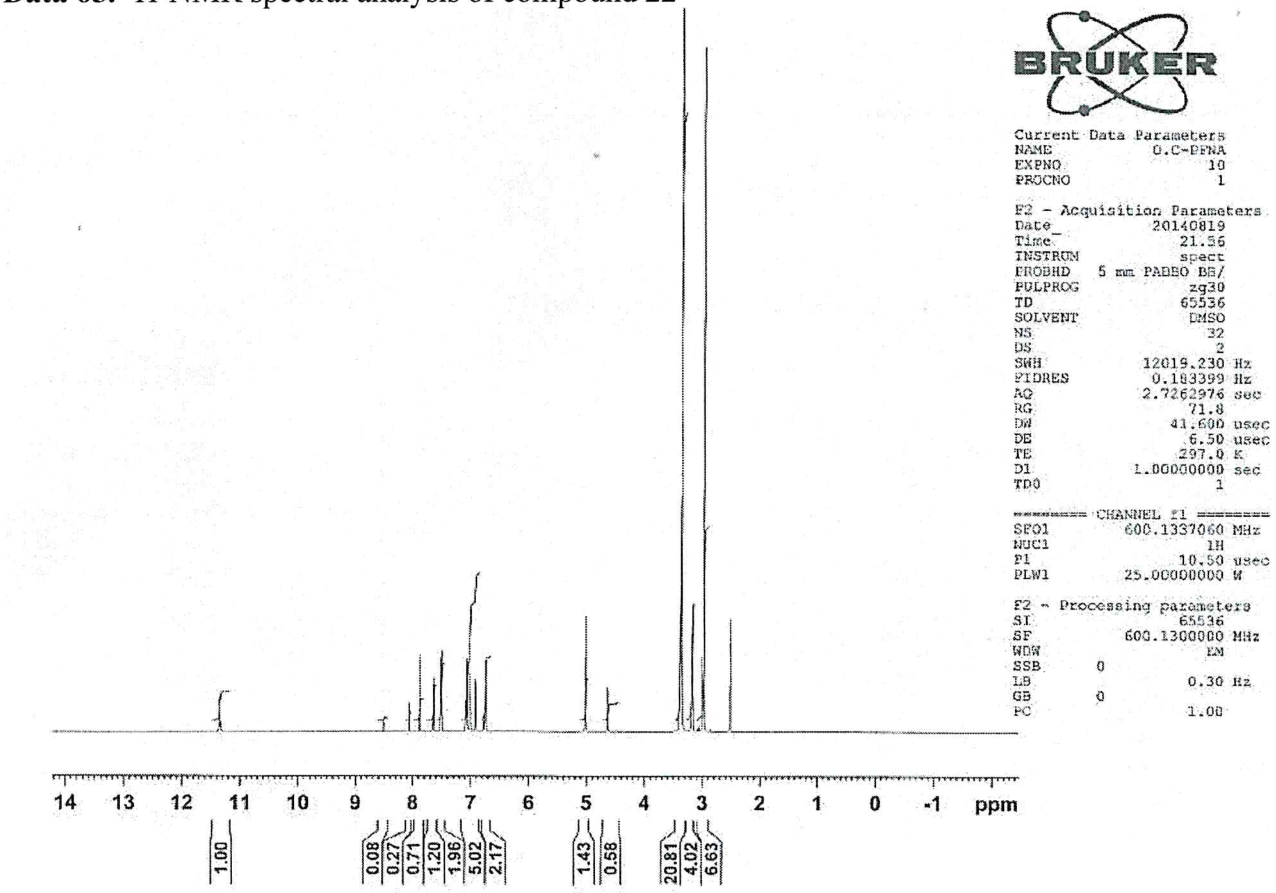

Data 66. ^{13}C-NMR spectral analysis of compound **22**

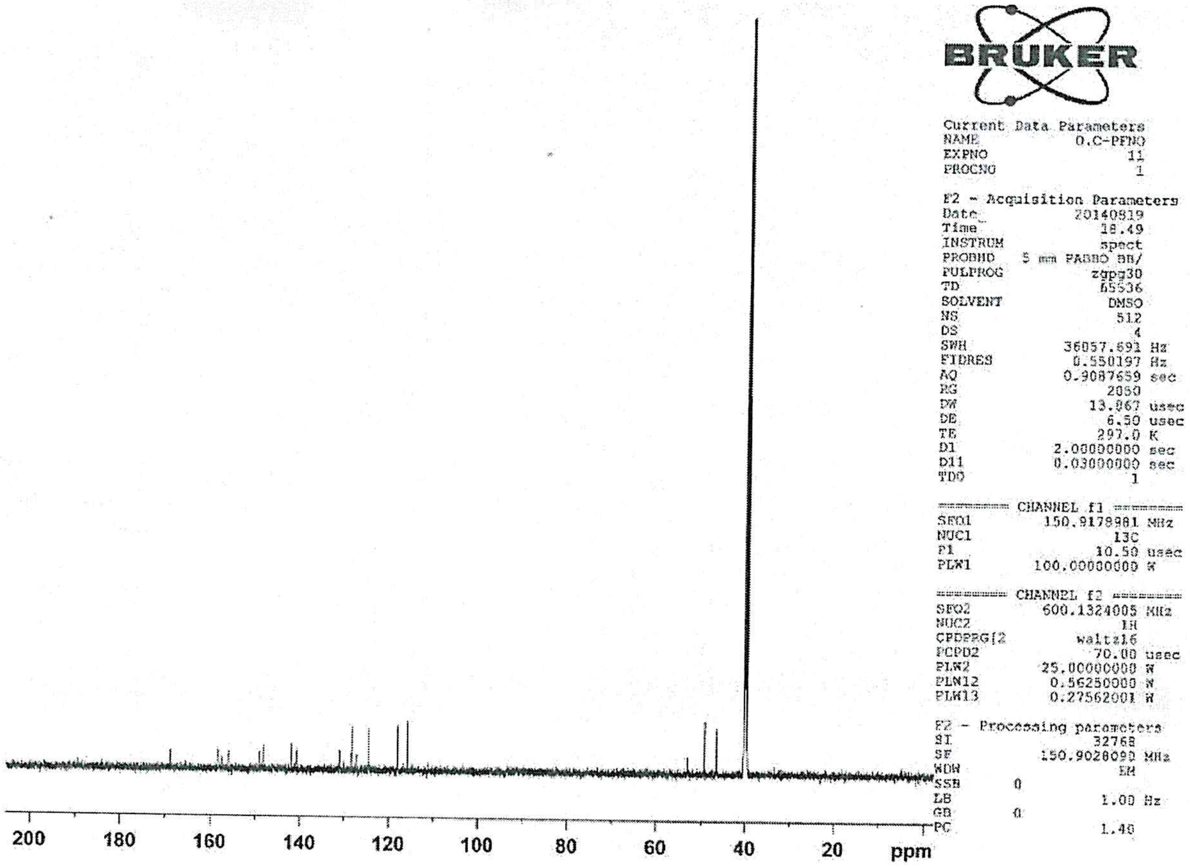

Data 67. Mass spectral analysis of compound **22**

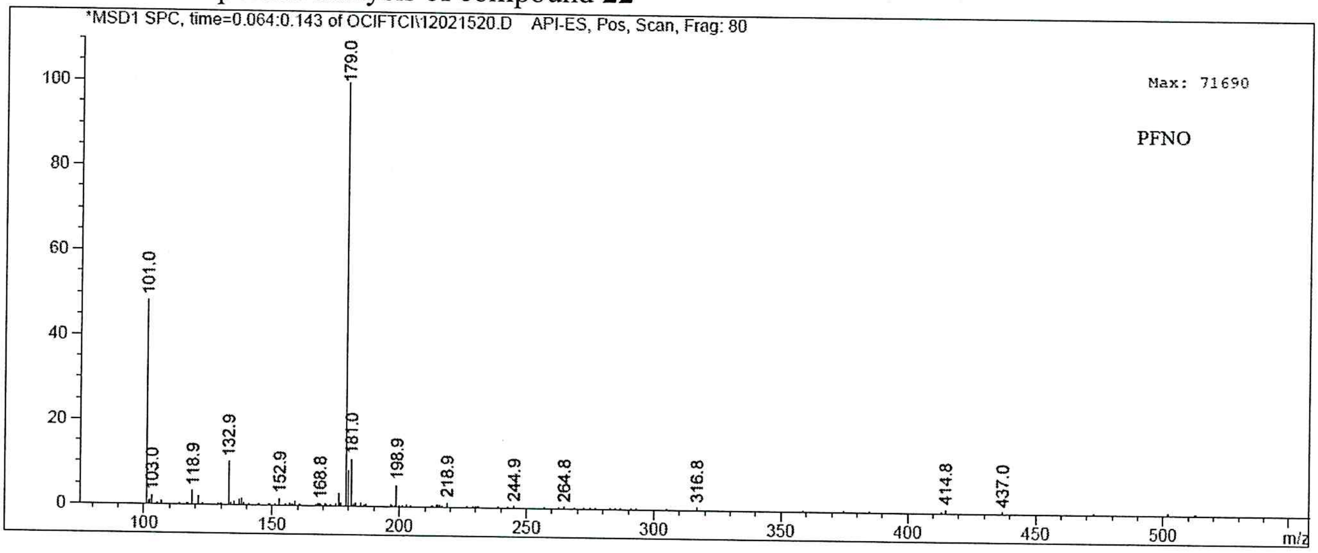

Data 68. ^{1}H-NMR spectral analysis of compound **23**

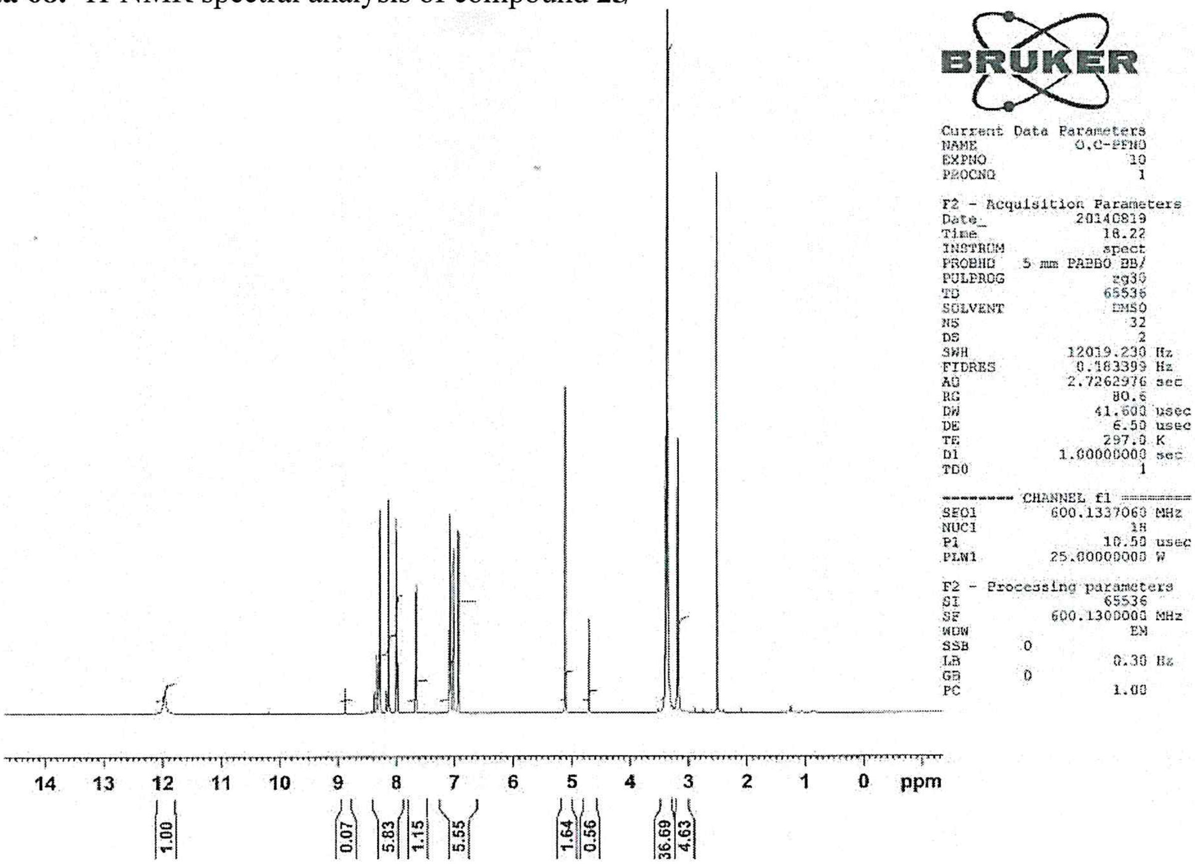

Data 69. ^{13}C-NMR spectral analysis of compound **23**

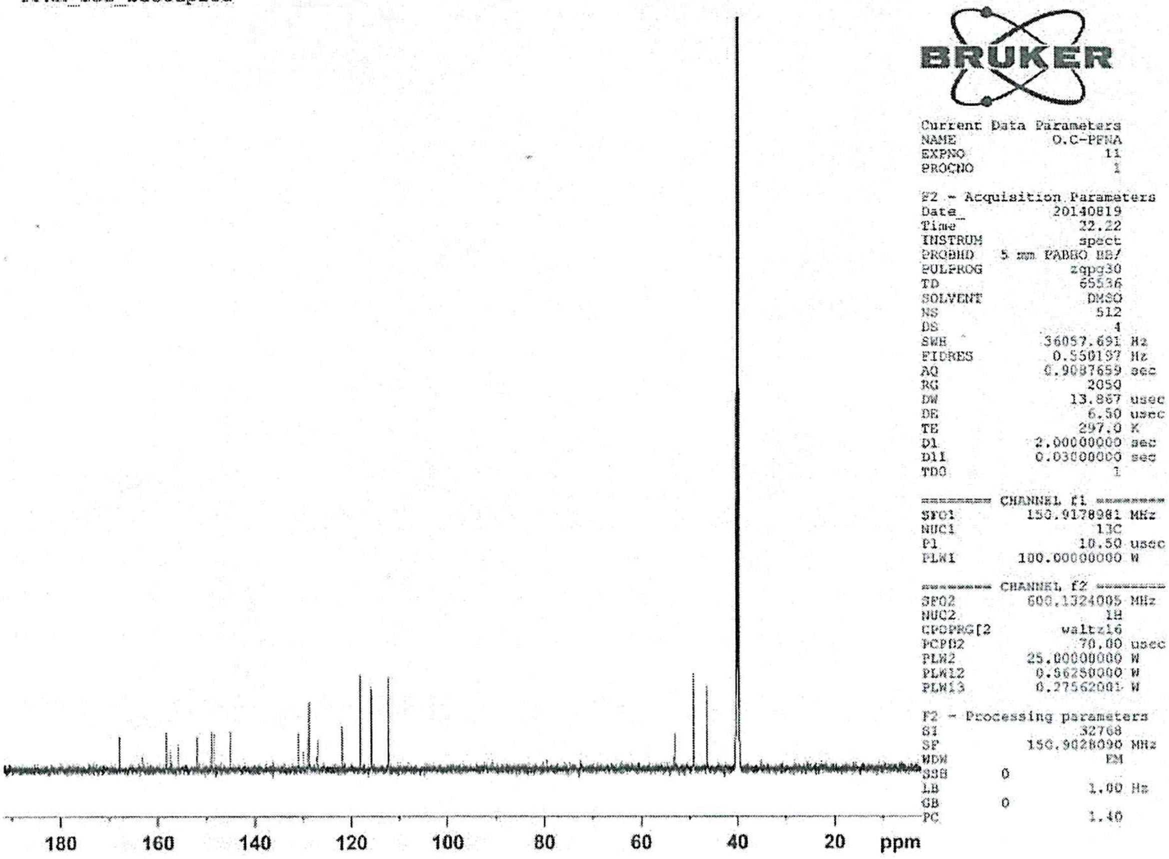

Data 70. Mass spectral analysis of compound **23**

Qualitative Analysis Report

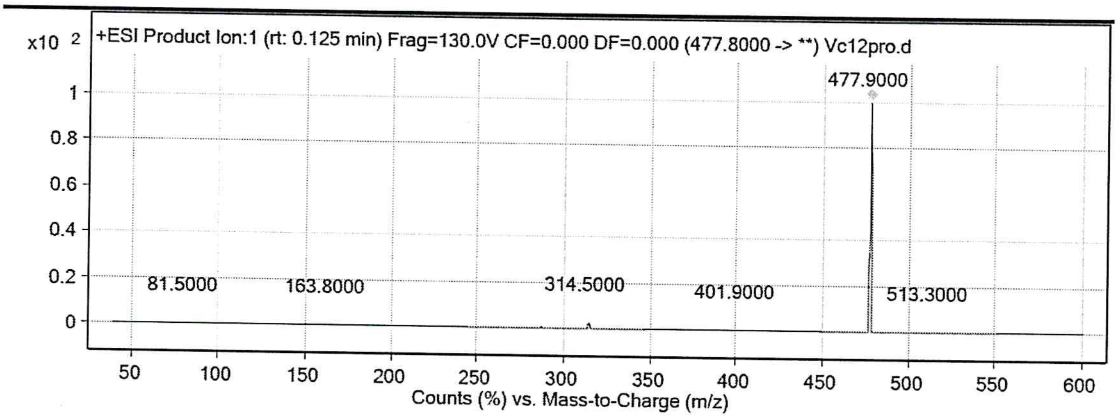

Peak List

m/z	z	Abund
163.8		166.08
176.5	2	54.06
259.5	2	53.76
286.8		206.02
314.5	2	980.46
332.1		50.7
472		51.46
476		64.92
477.9		36509.84
513.3		50.8
